# Tuning Eley–Rideal Mechanism in Electrochemical Acetylene Semi‐Hydrogenation

**DOI:** 10.1002/anie.202512218

**Published:** 2025-07-04

**Authors:** Chengyi Zhang, Jiguang Zhang, Zihao Jiao, Yanwei Lum, Ziyun Wang

**Affiliations:** ^1^ School of Chemical Sciences University of Auckland Auckland New Zealand; ^2^ Department of Chemical and Biomolecular Engineering National University of Singapore Singapore Republic of Singapore

**Keywords:** Catalyst design, ER mechanism, Experimental‐theoretical validation, Microkinetic modeling, Rate determining factor changing

## Abstract

The Eley–Rideal (ER) mechanism is pivotal in heterogeneous catalysis processes such as fuel cells and electrolyzers, which rely heavily on the interaction between solution‐phase and surface‐phase species. In this study, we examine the semi‐hydrogenation of acetylene to ethylene to explore the factors influencing the ER mechanism. We employed the density functional theory (DFT) to calculate the hydrogenation of acetylene on face‐centered cubic metals and copper‐based alloys. Microkinetic modeling identifies changes in the rate‐determining steps of different alloys as electronegativity decreases. We then constructed the volcano plot for the adsorption energy toward C_2_H_2_ and the reaction rate, which predicted that Cu_3_Au is the best candidate alloy for the C_2_H_2_ semi‐hydrogenation. Both extensive prior research and our experimental findings validated our volcano plot. Notably, our work points out the two key determinants of the ER mechanism: atomic activation and steric hindrance. For metals with weaker adsorption, steric hindrance primarily obstructs the ER mechanism, while for metals with stronger adsorption, the ER mechanism is hindered due to the challenge of atomic activation. Therefore, introducing weak adsorption sites into moderately adsorptive metals can improve the overall efficiency of the ER reaction by balancing these two factors.

## Graphical abstract

The ER mechanism plays a vital role in electrocatalytic hydrogenation, yet its rate‐limiting factors remain poorly understood. Using acetylene hydrogenation as a model, we uncover atomic activation and spatial hindrance as key ER bottlenecks. Cu_3_Au, with balanced adsorption strength, exemplifies how introducing weak‐binding atoms into moderate‐binding hosts can optimize ER activity.

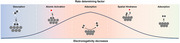

## Introduction

Catalysis plays an essential role in modern chemistry, powering key industrial processes such as energy conversion,^[^ ^1^ ^]^ chemical production,^[^ ^2^ ^]^ and environmental remediation.^[^ ^3^, ^4^ ^]^ Understanding catalysis requires uncovering the fundamental reaction mechanisms— how reactants interact with the catalyst to produce the desired products.^[^ ^5^ ^]^ These mechanisms are intimately connected with the nature and source of the reactants, which can either be surface‐bound or present in the surrounding environments. Therefore, a comprehensive understanding of the reaction mechanism and the source of reactants is crucial to improving catalytic efficiency and developing more sustainable processes.

Among the various catalytic processes, heterogeneous catalysis has become particularly significant due to its widespread applications in industrial processes such as petroleum refining, pollution control, and producing fine chemicals.^[^ ^6^, ^7^, ^8^ ^]^ In heterogeneous catalysis, the interaction between reactants, typically in the gas or liquid phase, and the solid catalyst surface is critical in determining the efficiency and selectivity of the reaction. These reactions occur at the catalyst surface and are governed by the surface's properties and the reactants' behavior. In electrocatalysis, the Eley–Rideal (ER) mechanism—also known as the solvent hydrogen transfer or proton‐coupled electron transfer (PCET) mechanism—is a crucial reaction pathway. However, it has received significantly less attention compared to the more extensively studied Langmuir–Hinshelwood (LH) mechanism, which involves surface hydrogen transfer. While the LH mechanism involves both reactants being adsorbed on the surface, the ER mechanism involves one reactant being absorbed on the surface and the other remaining in the solution phase. This unique interaction between the surface and the solution‐phase reactant introduces additional complexities that have yet to be fully understood. Given the growing importance of electrochemical applications, it is essential to delve deeper into the factors governing the ER mechanism to bridge the gap between surface‐bound and solution‐phase reactant interactions.

The semi‐hydrogenation of acetylene (C_2_H_2_) not only presents a substantial industrial value but also serves as an ideal model for understanding the ER mechanisms for several reasons.^[^ ^9^, ^10^, ^11^, ^12^, ^13^, ^14^, ^15^ ^]^ The first reason is the system's simplicity, which involves only two elements–carbon (C) and hydrogen (H), and one type of reaction, hydrogenation. It avoids the complexity of multi‐element systems and provides clear insights into how ER mechanisms operate. Besides, the limited set of intermediate species (*HCCH_,_ *HCCH_2,_ *H_2_CCH_2,_ *H) offers a transparent and controllable platform for experimental and theoretical studies.

Secondly, the hydrogenation of the acetylene toward ethylene proceeds through two distinct steps that provide insights into both symmetric and asymmetric catalytic processes. The hydrogenation of *HCCH involves two identical carbon atoms, where we can learn how adsorbed sites affect the reaction. The second step, hydrogenation of the *HCCH_2_, involves two carbon atoms with different saturation levels. The ability to study both symmetric and asymmetric reactions within the same system makes the semi‐hydrogenation of acetylene uniquely valuable for expanding our understanding of how ER behaves in a wide range of catalytic processes. Such a process will be especially valuable for heterogeneous catalysis. Although there is extensive literature on the semi‐hydrogenation of acetylene in catalysis,^[^ ^12^, ^13^, ^16^, ^17^, ^18^, ^19^, ^20^ ^]^ how the hydrogenation reaction happens and what affects the ER mechanism is rare, making detailed investigations into such reactions quite important.

We calculated the Gibbs energy change and the activation energy barrier of each step of the semi‐hydrogenation of acetylene on various close‐packed metals (Ag, Au, Cu, Ni, Fe) and their Cu alloys (CuAg_3_, CuAu_3_, CuNi_3_, CuFe_3_, Cu_3_Ag, Cu_3_Au, Cu_3_Ni, and Cu_3_Fe) then performed the microkinetic analysis. For simplicity, we labeled (CuAg_3_, CuAu_3,_ Cu_3_Ni, and Cu_3_Fe) as AB_3_ and (Cu_3_Ag, Cu_3_Au, CuNi_3_, CuFe_3_) as BA_3_ (A denotes the metal atom with a stronger affinity toward intermediates, B denotes the metal atom with a weaker affinity toward intermediates). The hydrogenation of acetylene on various metal surfaces typically proceeds via a PCET mechanism, in which the proton and electron are transferred simultaneously. This type of reaction is a prototypical case where the computational hydrogen electrode (CHE) model can be reliably applied. In PCET steps, the free energy of the transition state and final state varies approximately linearly with the applied electrode potential, allowing the CHE approach to capture potential‐dependent energy profiles without the need for explicit charge control.^[^ ^21^, ^22^, ^23^ ^]^ Therefore, in this work, we employ the CHE model to describe the potential‐dependent energetics of the hydrogenation of acetylene across different metal surfaces, including the changes in free energy of reactants, intermediates, and transition states as a function of applied potential. The reason we choose these metals is given in the Supporting Information in detail. These calculations show that the hydrogenation barrier of *HCCH is closely related to the bonding metals, and the solvent hydrogen tends to attack the C atom, which binds weakly. For the hydrogenation of *HCCH_2_, the CH_2_ group could prevent unsaturated atoms from the hydrogenation of solvent hydrogen, and the activation energy barrier is linearly related to the angle between the C═C bond and the x‐o‐y plane. The microkinetic modeling revealed a transition of the rate‐determining step with the increasing affinity toward intermediates of metals. We construct the volcano plot of the activity versus adsorption energy toward C_2_H_2,_ identifying the optimized catalysts for semi‐hydrogenation of C_2_H_2_. This volcano plot successfully explains the performance of all related works. We also make further experimental validation in different regions of the volcano plot. The excellent agreement between our experiment and calculation results powerfully proves the robustness of our approach. Beyond identifying the optimal catalysts for the semi‐hydrogenation of acetylene, we also give a clear picture of the ER mechanism and provide broad implications for heterogeneous catalysis.

## Results and Discussion

**Figure 1:**
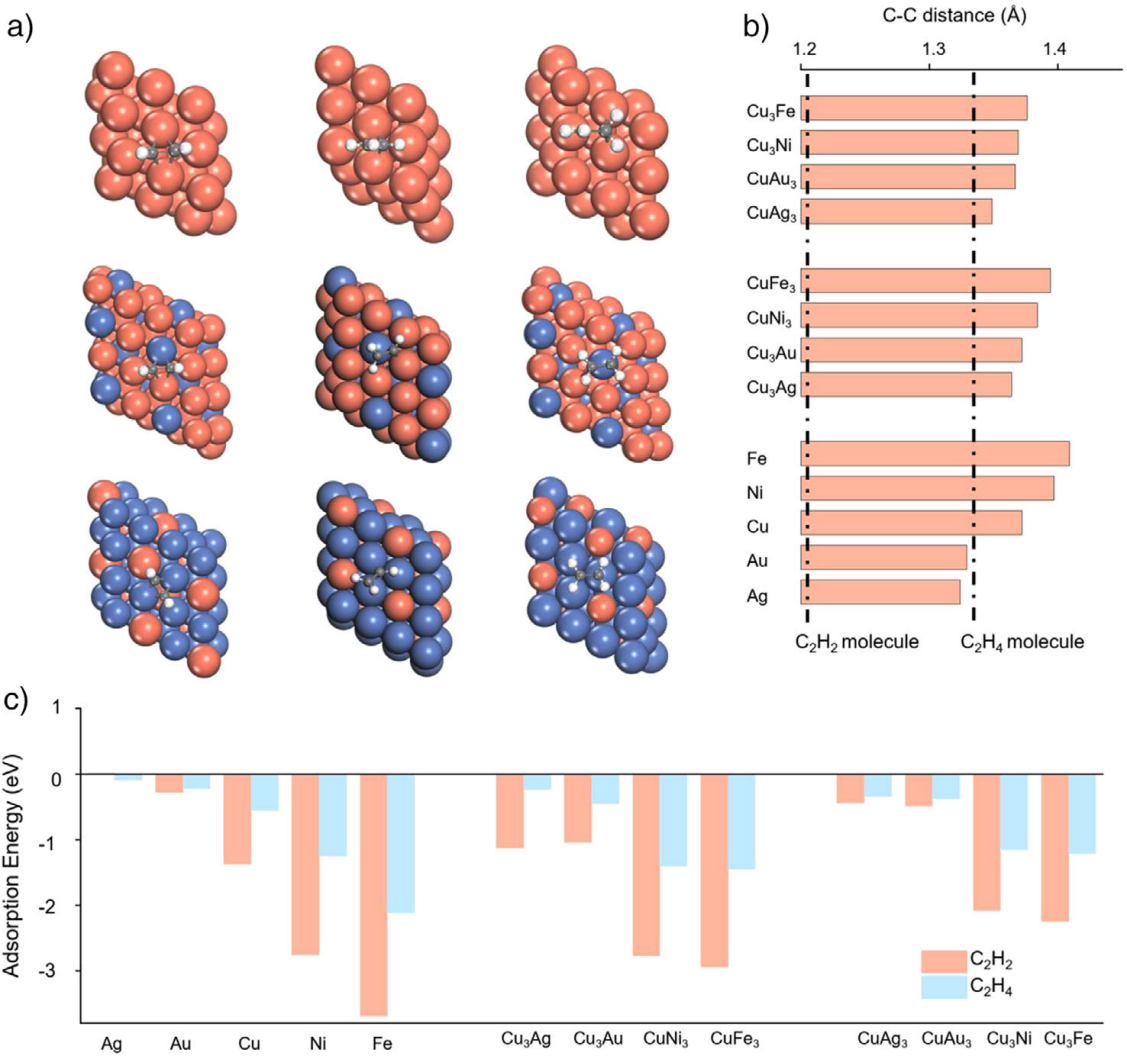
Molecular configuration and adsorption energy when C_2_H_2_ and C_2_H_4_ are absorbed on different slabs. a) Different adsorption configurations of *HCCH, *HCCH_2_, and *H_2_CCH_2_ on pure metal, AB_3_, A_3_B. Here, we take the adsorption configurations on the Cu, Cu_3_Ni, and CuNi_3_ as examples. b) C≡C bond length of HCCH* on different slabs. c) The adsorption energy of C_2_H_2_ and C_2_H_4_ on different slabs.

We first examine the adsorption behaviour of C_2_H_2_ on various metal and alloy surfaces. As shown in Figure 1a, three distinct adsorption configurations are observed across different materials. On pure metals, the two carbon atoms of *HCCH preferentially adsorb at adjacent hollow sites. In contrast, on BA_3_ alloys, one carbon atom tends to occupy the hollow site formed by three A atoms, while the other favours the hollow site involving A─A─B atoms, near the A─A bridge. For AB_3_ alloys, both carbon atoms are generally adsorbed on a single A atom, forming a more stable configuration.

In the case of *HCCH_2_, the adsorption configurations vary significantly. On Ag and Au surfaces, the interaction with *HCCH_2_ is weak; the unsaturated carbon atoms tend to locate near bridge sites adjacent to top sites. On CuAg_3_ and CuAu_3_, these carbon atoms are primarily adsorbed on Cu atoms, while the CH_2_ group remains nearly desorbed, as shown in Figures S66, S67. As the affinity toward intermediates increases (e.g., Cu_3_Ag and Cu_3_Au), the unsaturated carbon atoms settle at hollow sites, with partial adsorption of the CH_2_ group. On stronger‐binding surfaces such as Cu, Ni, Fe, CuNi_3_, and CuFe_3_, the carbon atoms occupy central hollow sites, with particularly strong adsorption observed on Ni and Fe, where the CH_2_ group is firmly anchored to the surface.

For *H_2_CCH_2_, adsorption is weak on Ag, Au, CuAg_3_, CuAu_3_, Cu_3_Ag, and Cu_3_Au, facilitating its smooth desorption. In contrast, on the remaining catalysts, both carbon atoms are strongly bound to the surface. We also analysed the C≡C bond of *HCCH on various substrates, comparing it with the C≡C bond in free C_2_H_2_ and the C═C bond in C_2_H_4_ (Figure 1b). A clear trend emerges that stronger adsorption correlates with greater C≡C bond elongation. For weakly interacting metals like Ag and Au, minimal C≡C bond stretching suggests poor activation upon adsorption, consistent with their negligible adsorption energies. The charge density difference plot (Figure S85) and the PDOS (projected density of states) of the C‐2p orbital (Figure S86) also confirm our results. The greater the electron transfer from the metal surface to *HCCH, the stronger the adsorption. This indicates that electron donation from the metal facilitates acetylene activation. In addition, we analysed the projected density of states (PDOS) of the C 2p orbitals in *HCCH. Relative to the gas‐phase acetylene molecule, the adsorbed species exhibits greater delocalization of electronic states, indicating stronger interaction and activation by the metal surface. Among the metals examined, only Au and Ag display two minor peaks near the Fermi level, pointing to weaker orbital hybridization and limited activation of adsorbed acetylene. These findings align well with the trends shown in Figure 1b and reinforce our conclusion that most metals—except Au and Ag—can effectively activate *HCCH through pronounced electronic interactions.

### Configuration and Energy Barrier Relationships

**Figure 2:**
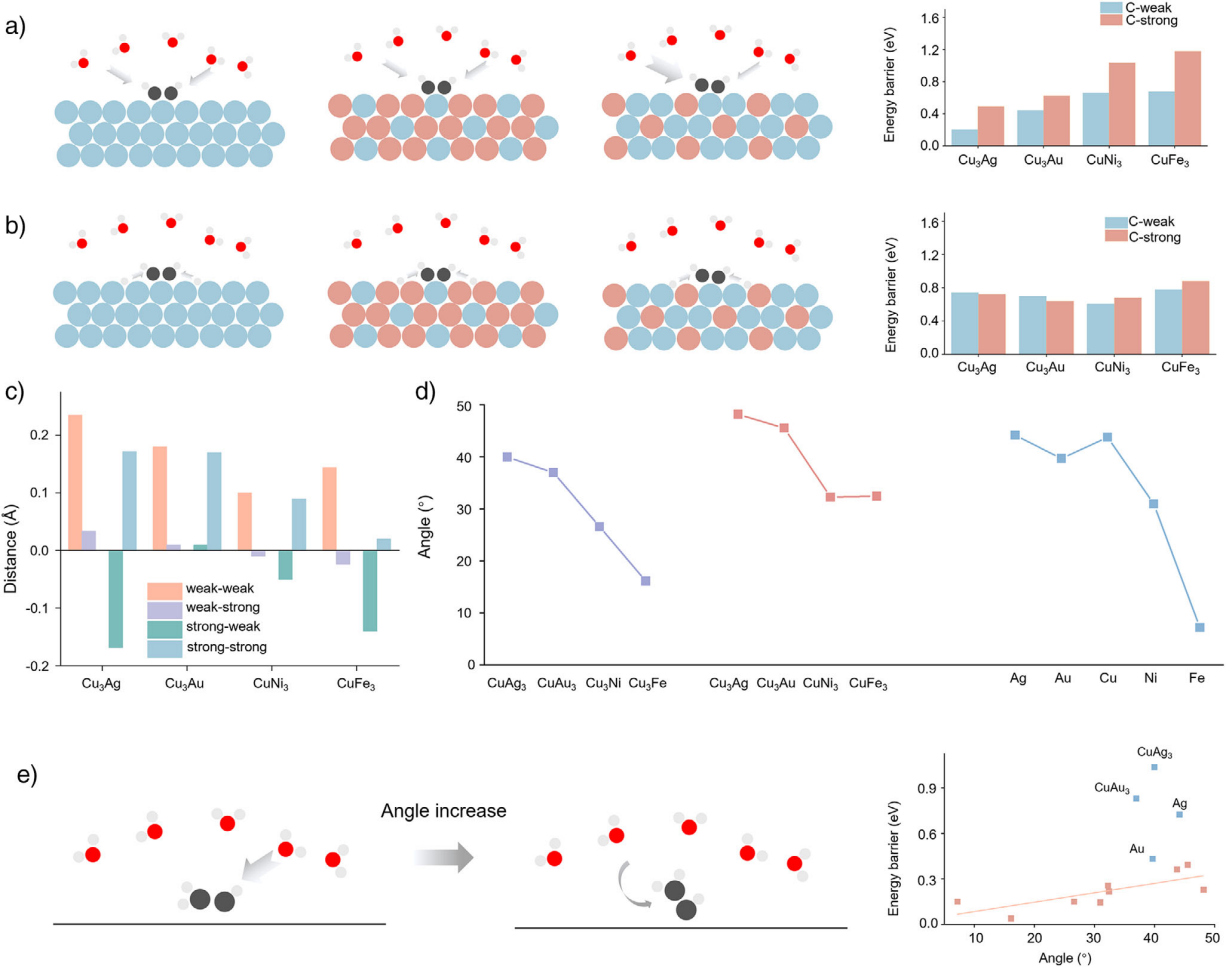
Activation energy barrier and structural change of the hydrogenation reaction at 0 V versus RHE. a) The energy barrier of the ER mechanism on different C in *HCCH. b) The energy barrier of the LH mechanism on different C in *HCCH. Blue atom indicates the metal atom with a stronger affinity toward intermediates, and red atoms indicate the metal atom with a lower affinity toward intermediates. c) The distance change of C between the initial and transition states during the hydrogenation of *HCCH along the z‐axis. (weak‐strong indicates that when the hydrogenation occurs on the C‐weak, the elevated distance for the C‐strong, the rest (weak–weak, strong–weak, and strong–strong) follow in the same manner) d) The angle between the C═C bond of *HCCH_2_ and the x‐o‐y plane on different surfaces. e) The energy barrier of hydrogenation of *HCCH_2_ by the ER mechanism on various surfaces with the change of angle between the two carbon atoms and the x‐o‐y plane.

We then conducted detailed investigations into the activation energy barrier. The second step is the hydrogenation of *HCCH; as described in Figure 1a, the carbon atoms of *HCCH share the same chemical environment in the pure metal and the AB_3_ alloys. We thus emphasize the hydrogenation of *HCCH on A_3_B alloys. The carbon on the hollow site with three strong‐affinity atoms is labeled C‐strong, while the other is labeled C‐weak. The activation energy barrier of two carbon atoms is investigated separately in Figure 2a; we found that the activation energy barrier of the C‐weak is generally lower than that of the C‐strong; with the same solvent environment and coordination number, the factors could be attributed to the coordination of metals. The structures of the initial and transition states of the hydrogenation of *HCCH on A_3_B alloys are presented in Figures S7–S10. The carbon atom tends to elevate and be activated for hydrogenation during the ER mechanism. Figure 2c provides numerical evidence. We compare the z coordinates of the transition state with the initial states; the carbon atoms to be attacked by the hydrogen atoms will be elevated. The carbon atom on the Cu_3_Ag rises most, followed by Cu_3_Au, CuNi_3_, and CuFe_3._ Interestingly, the activation energy barrier for carbon atoms also generally follows this trend. The easier the carbon atoms rise from the slab, the easier they are activated, which leads to a lower activation energy barrier. Regarding the hydrogenation of *HCCH_2_, we divided *HCCH_2_ into three categories according to the previous adsorption pattern. With the increase in affinity, the angle between the C═C bond and the x‐o‐y plane in all three adsorption modes shows a decreasing trend. We then investigate the relationship between the activation energy barrier and this angle. Surprisingly, apart from several outliers, the activation energy barrier follows the linear relationship with the angle in Figure 2e. For these outliers (Ag, Au, CuAg_3_, CuAu_3_), we found that the CH_2_ group of *HCCH_2_ on these metals is nearly free from the adsorption of the slab (presented in Figure S1). Such a free CH_2_ group could act as an umbrella to prevent the solvent hydrogen from attacking the unsaturated carbon atoms. Based on the ER mechanism, we derive two general principles. First, surface atoms tend to elevate during activation in the ER process. However, stronger adsorption energies make it more difficult for these atoms to rise and participate in hydrogenation. Second, when reactive intermediates possess free functional groups, these groups can behave like umbrellas, shielding the surface atoms and hindering hydrogenation. In such cases, metals with stronger adsorption capabilities can anchor these free groups to the surface, thereby facilitating effective hydrogenation of the reactive center. For the LH mechanism, we find no distinct difference in the activation energy barrier in the hydrogenation of *HCCH in Figure 2b (the detailed structures are presented in Figures S15–S27).

### Rate‐Determining Steps Transition

**Figure 3:**
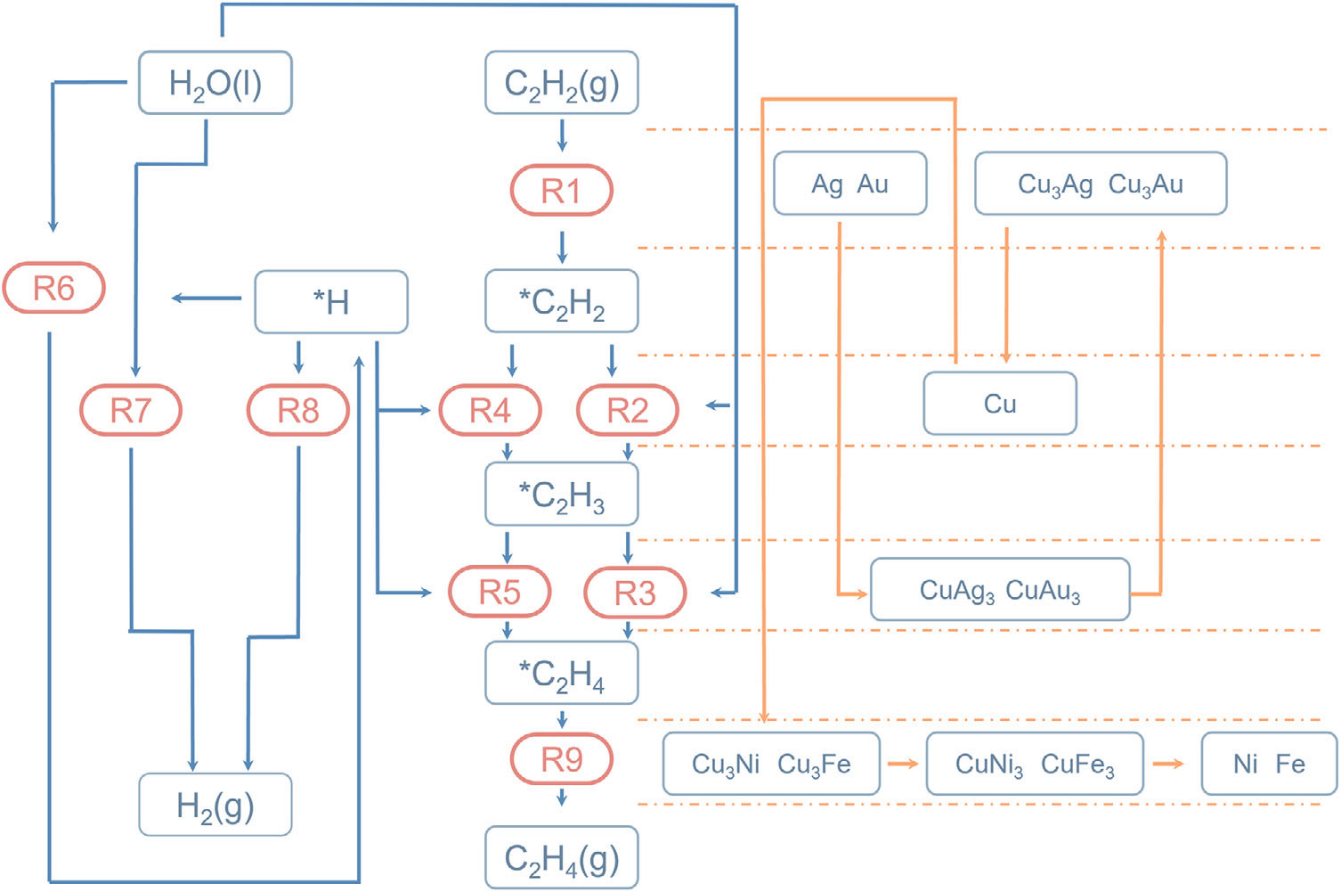
The full flow chart of the reaction pathways and the rate‐determining step of each alloy. All elementary reactions are listed in the Table S1.

Microkinetic modeling provides a detailed mechanistic insight into the semi‐hydrogenation of acetylene, as depicted in Figure 3. The overall reaction comprises four fundamental steps: (1) acetylene adsorption, (2) the first hydrogenation to form *HCCH_2_, (3) further hydrogenation of *HCCH_2_ to form *H_2_CCH_2_, and (4) ethylene desorption.

In the first step, acetylene adsorption is significantly influenced by the metal's affinity toward the adsorbate. For noble metals such as Ag and Au, which exhibit low adsorption energies, acetylene binds weakly to the surface. As a result, this step becomes kinetically unfavourable and emerges as the rate‐determining step (RDS) for these metals. The inability to effectively adsorb and activate acetylene restricts subsequent transformations.

Upon alloying with Cu to form CuAg_3_ and CuAu_3_, the presence of isolated Cu atoms introduces moderately active sites that facilitate acetylene adsorption. However, the geometry of the resulting *HCCH_2_ intermediate on these alloys presents a challenge. Typically, one unsaturated carbon atom of the intermediate adsorbs on top of the Cu atom, while the CH_2_ group, oriented toward adjacent Au or Ag atoms, remains poorly stabilized due to their weak binding interactions. This results in a spatial configuration where the loosely anchored CH_2_ group hinders access to hydrogen atoms, thereby impeding the subsequent hydrogenation step. Consequently, the third step—hydrogenation of *HCCH_2_—becomes the RDS for these alloy systems.

As the Cu content increases further in alloys like Cu_3_Ag and Cu_3_Au, the surface exhibits a more balanced adsorption environment. The increased number of Cu sites enhances the adsorption and activation of the unsaturated carbon atom, while the moderate binding energy of the surrounding atoms provides sufficient anchoring of the CH_2_ group. This configuration reduces the energetic barrier for the hydrogenation of *HCCH_2_, enabling smoother progression through this intermediate. In such systems, the surface can efficiently adsorb acetylene, carry out rapid sequential hydrogenation, and allow the desorption of ethylene, shifting the RDS back to the initial acetylene adsorption step, as it becomes the slowest among otherwise rapid steps.

On even more strongly adsorbing surfaces, such as pure Cu, the two carbon atoms of *HCCH bind tightly to the hollow sites of the surface. While this stabilizes the intermediate, it also makes structural rearrangement and hydrogenation of *HCCH energetically demanding. In these cases, the second step—hydrogenation of *HCCH—becomes kinetically limiting due to the difficulty in elevating and activating the tightly adsorbed carbon atoms.

Finally, for metals and alloys with very high adsorption affinity (e.g., Ni‐rich surfaces), the product ethylene binds too strongly to the surface. Although the reaction proceeds smoothly through the earlier hydrogenation steps, the desorption of ethylene becomes sluggish. The product accumulates on the surface, occupying active sites and leading to catalyst deactivation through surface poisoning. Under these conditions, ethylene desorption becomes the RDS, thereby limiting the overall catalytic turnover.

Taken together, this analysis reveals how the rate‐determining step evolves systematically with adsorption strength: from acetylene adsorption (weak‐binding metals), to *HCCH_2_ hydrogenation (moderate alloys), to *HCCH hydrogenation (strong‐binding metals), and finally to ethylene desorption (very strong‐binding systems). This sequence highlights the importance of tuning adsorption strength to achieve optimal catalytic performance in the semi‐hydrogenation of acetylene.

### Comprehensive Activity Analysis

**Figure 4:**
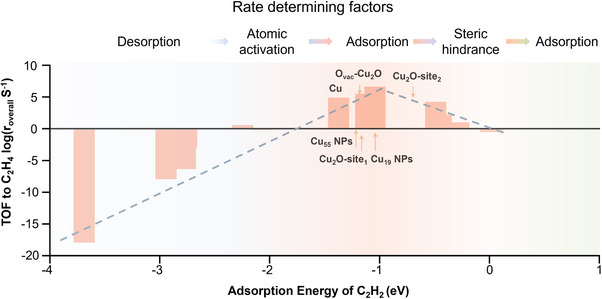
The volcano plot of turnover frequency (TOF) to adsorption energy toward C_2_H_2_ based on the close‐packed metals and Cu alloys under −0.3 V versus RHE. The previously reported materials used to convert C_2_H_2_ to C_2_H_4_ by electrochemistry are also labeled here. The column indicates the Ag, Au, CuAg_3_, CuAu_3_, Cu_3_Au, Cu_3_Ag, Cu, Cu_3_Ni, Cu_3_Fe, CuNi_3_, Ni, CuFe_3_, Fe with the adsorption energy toward C_2_H_2_ getting substantial.

To further elucidate the catalytic performance across different metals, we performed microkinetic modeling to calculate the semi‐hydrogenation activity of acetylene, as shown in Figure 4. The analysis reveals a volcano‐type trend in activity as a function of C_2_H_2_ adsorption energy. Initially, as the adsorption energy increases, the catalytic activity improves, reaching a maximum at an optimal range before declining with further increase in binding strength. Correspondingly, the RDS evolves sequentially: starting from R1 (acetylene adsorption), transitioning to R3 (hydrogenation of *HCCH_2_), reverting back to R1, then shifting to R2 (hydrogenation of *HCCH), and finally to R9 (ethylene desorption) as the adsorption becomes excessively strong. All reactions (R1‐R9) related are listed in Table S1 in detail.

The highest catalytic activity is observed in the intermediate regime, where R1 again becomes the RDS—a region occupied by Cu_3_Ag and Cu_3_Au alloys. This observation aligns well with our earlier mechanistic deductions based on the geometric and electronic characteristics of intermediates and their interaction with moderately binding surfaces.

We also explored the current literature on the electrochemical semi‐hydrogenation of acetylene. Yeo and co workers reported that Cu_2_O exhibits superior catalytic activity compared to metallic Cu. On Cu surfaces, the RDS is identified as the hydrogenation of *HCCH, primarily due to its strong adsorption energy, which impedes atomic activation.^[^ ^24^ ^]^ The presence of oxygen in Cu_2_O appears to modulate the surface electronic properties by withdrawing electron density from adjacent Cu atoms (as shown in Figure S54), thereby weakening the overall adsorption strength and improving hydrogenation kinetics. Building upon this, Wang et al. reported the superior performance of O_vac_‐Cu_2_O over pristine Cu_2_O.^[^ ^13^ ^]^ To understand this, we calculated the adsorption energy of Cu_2_O. The results show that the adsorption energy of Cu_2_O‐site1 is lower than that on metallic Cu and approaching the optimal range identified in our volcano plot. However, the majority of C_2_H_2_ molecules adsorb on weaker‐binding Cu_2_O sites, such as those with an adsorption energy of −0.79 eV (Cu_2_O‐site2, most available sites), which limits the overall catalytic efficiency (Figures S56–S57). Interestingly, such O_vac_‐Cu_2_O configurations resemble that of Cu_3_Au: one carbon atom of the *HCCH_2_ intermediate binds moderately, while the other, adjacent to the oxygen vacancy, experiences weaker binding (Figure S58). This asymmetry enables facile activation of the less‐anchored carbon atom during the first hydrogenation step, while the partially stabilized CH_2_ group remains accessible for subsequent reactions. These findings support the notion that incorporating weaker‐affinity sites on Cu surfaces can enhance ER‐type hydrogenation, a strategy also validated by Zhang and co‐workers in their studies on O_vac_‐Cu_2_O catalysts.^[^ ^13^ ^]^ This framework also rationalizes the outstanding activity of Cu nanoparticles (NPs) reported in prior studies.^[^ ^12^, ^20^ ^]^ The calculated C_2_H_2_ adsorption energies on Cu_19_ and Cu_55_ NPs are −1.07 and −1.29 eV, respectively, both situated near the apex of our activity volcano. Moreover, a unique structural feature of Cu NPs is the preferential adsorption of intermediates at the edge or corner sites, which are more exposed to the solvent environment.^[^ ^25^ ^]^ Exposing the intermediates to the solvent facilitates the hydrogenation(Figures S59–S62).

### Experimental Validation

To further validate our computational results, we conducted a series of acetylene reduction experiments on a range of alloy catalysts, including Cu_6_Ag_94_, Cu_11_Au_89_, Cu_40_Ag_60_, Cu_72_Ag_28_, Cu_79_Au_21_, pure Cu, and Cu_82_Ni_18_. In the HER, both the Volmer step (proton adsorption) and the Heyrovsky step are potential‐dependent electrochemical processes, and their rates increase rapidly with increasing applied potential. In contrast, the acetylene reduction reaction involves chemical adsorption and desorption steps that are relatively less sensitive to potential changes. Specifically, the initial adsorption of acetylene and the final desorption of ethylene are chemical in nature and thus not significantly accelerated by higher potentials.

As a result, the FE for hydrogen production increases markedly with increasing potential, while the FE for acetylene hydrogenation products decreases. Therefore, operating under lower current (or lower potential) conditions is more favourable for selectively producing ethylene, as it helps suppress HER and enhances the FE of acetylene reduction.

**Figure 5:**
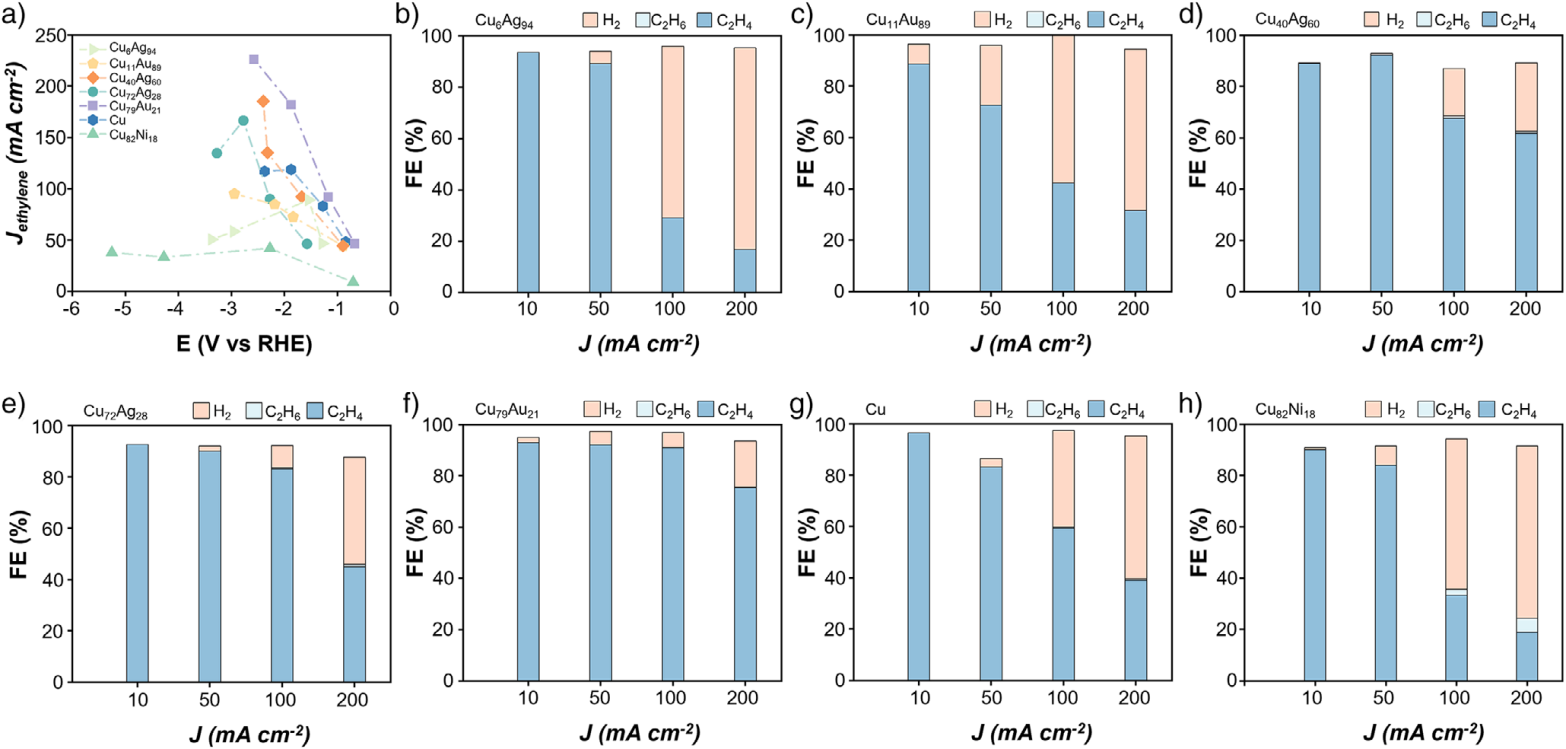
Experimental validations for volcano curves. a) Partial current densities of ethylene products of various alloys under different potentials. Product FE for b) Cu_6_Ag_94_, c) Cu_11_Au_89,_ d) Cu_40_Ag_60,_ e) Cu_72_Ag_28_, f) Cu_79_Au_21_, g) Cu, and h) Cu_82_Ni_18_ under different current densities.

Our experimental observations show that the catalytic activity of the alloys follows a volcano‐like trend: it increases in the order of Cu_6_Ag_94_, Cu_11_Au_89_, Cu_40_Ag_60_, and Cu_72_Ag_28_, reaching a peak at Cu_79_Au_21_, which corresponds closely to the Cu_3_Au model used in our DFT calculations(Figure 5a). The activity then declines for pure Cu and Cu_82_Ni_18_ as the adsorption strength toward intermediates becomes too strong. These experimental results are in excellent agreement with the theoretical volcano curve presented in Figure 4, reinforcing the predictive power of our simulations (Figure 5).

For weakly adsorbing alloys such as Cu_6_Ag_94_ and Cu_11_Au_89,_ the desorption of ethylene is facile, and the reaction is not hindered by surface accumulation of products. Due to insufficient adsorption strength, over‐hydrogenation to ethane (C_2_H_6_) is not observed. As the adsorption strength increases, ethane formation begins to emerge, with trace amounts detected on Cu_40_Ag_60_, Cu_72_Ag_28_, and pure Cu. In the case of Cu_82_Ni_18_, the adsorption is excessively strong, leading to noticeable ethane production and potential surface poisoning (Figure 5b–h). Consequently, the current density for ethylene formation on Cu_82_Ni_18_ shows minimal enhancement with increasing potential, corresponding with the results in Figure 3.

These experimental findings strongly support our theoretical prediction that acetylene can be effectively converted to ethylene and desorbed on Cu_3_Au alloy surfaces. Together, our experiments and simulations consistently demonstrate the validity of the ER mechanism, selectivity, and the reliability of the volcano relationship we proposed, offering valuable guidance for the rational design of selective catalysts in future studies.

## Conclusions

This work aims to gain deep insights into ER mechanisms based on the semi‐hydrogenation reaction of C_2_H_2_. We studied the stable *HCCH configuration across various metal catalysts, identifying three different configuration types on pure metal, AB_3_, and BA_3_ alloys. All investigated catalysts can effectively activate C_2_H_2,_ except Ag and Au. Then, for the first hydrogenation step, activation requires the carbon atom to lift from the surface, making *HCCH more easily activated on metals with weaker adsorption. During the hydrogenation of *HCCH_2_, the activation energy barrier increases as the angle between the two carbon atoms and the x‐o‐y plane rises. This could be attributed to the fact that the CH_2_ group becomes less constrained by the surface, hindering the hydrogen attack from the solution on the surface carbon atom. At this stage, more substantial adsorption energy could lock the CH_2_ on the surface and expose the unsaturated carbon atoms to the hydrogen from the solution. For the LH mechanism, the activation energy barrier does not differ significantly. Overall, we find that with affinity increases, the rate‐determining step shifts from the first step of acetylene adsorption to the hydrogenation of *HCCH_2,_ then back to the adsorption of acetylene, hydrogenation of *HCCH, and finally to the desorption of ethylene. Microkinetic modeling was then performed to construct the volcano plot relationship between the acetylene adsorption energy and reaction rate, explaining why Cu_2_O, O_vac_‐Cu_2_O, and copper nanoparticles perform well in related studies. Such profound ER mechanism insights can be extended to all heterogeneous reactions, providing valuable insights for catalyst design and pathway optimization.

## Supporting Information

The authors have cited additional references within the Supporting Information.^[^ ^22^, ^23^, ^26^, ^27^, ^28^, ^29^, ^30^, ^31^, ^32^, ^33^, ^34^, ^35^, ^36^, ^37^, ^38^, ^39^, ^40^, ^41^, ^42^, ^43^, ^44^, ^45^, ^46^, ^47^, ^48^, ^49^, ^50^, ^51^ ^]^

## Author Contributions

Z.W. and Y.L. supervised the project. C.Z. and Z.W. conceived the idea and designed the experiments. C.Z. performed the computational work. J.Z. carried out the experimental work. C.Z. and J.Z. contributed equally. All authors discussed the results and assisted during the manuscript preparation.

## Conflict of Interests

The authors declare no conflict of interest.

## Supporting information



Supporting Information

## Data Availability

The data that support the findings of this study are available from the corresponding author upon reasonable request.

## References

[1] L. Tang , X. Meng , D. Deng , X. Bao , Adv. Mater. 2019, 31, e1901996. 31390100 10.1002/adma.201901996

[2] T. J. Schwartz , B. J. O'Neill , B. H. Shanks , J. A. Dumesic , ACS Catal. 2014, 4, 2060–2069.

[3] Y. Shang , X. Xu , B. Gao , S. Wang , X. Duan , Chem. Soc. Rev. 2021, 50, 5281–5322. 33904567 10.1039/d0cs01032d

[4] Y. Fang , Y. Guo , J. Catal. 2018, 39, 566–582.

[5] G. J. Hutchings , Faraday Discuss. 2021, 229, 9–34. 34075992 10.1039/d1fd00023c

[6] X. Hu , A. C. K. Yip , Front. Catal. 2021, 1, 667675.

[7] C. M. Friend , B. Xu , Chem. Res. 2017, 50, 517–521. 10.1021/acs.accounts.6b00510 28945397

[8] S. N. Khadzhiev , Pet. Chem. 2011, 51, 1–15.

[9] H. Y. Fang , J. J. Jiang , D. S. Wang , X. W. Liu , D. R. Zhu , Y. D. Li , Acta. Phys. Chim. Sin. 2023, 39.

[10] Y. Q. Cao , H. Zhang , S. F. Ji , Z. J. Sui , Z. Jiang , D. S. Wang , F. Zaera , X. G. Zhou , X. H. Duan , Y. D. Li , Chem. Int. Ed. 2020, 59, 11647–11652. 10.1002/anie.202004966 32282112

[11] H. R. Zhou , X. F. Yang , A. Q. Wang , S. Miao , X. Y. Liu , X. L. Pan , Y. Su , L. Li , Y. Tan , T. Zhang , J. Catal. 2016, 37, 692–699.

[12] F. Chen , L. Li , C. Cheng , Y. Yu , B. H. Zhao , B. Zhang , Nat. Commun. 2024, 15, 5914. 39003284 10.1038/s41467-024-50335-8 PMC11246534

[13] Z. Wu , J. Zhang , Q. Guan , X. Liu , H. Xiong , S. Chen , W. Hong , D. Li , Y. Lei , S. Deng , J. Wang , G. Wang , Adv. Mater. 2024, 36, e2408681. 39155581 10.1002/adma.202408681

[14] B. Yang , R. Burch , C. Hardacre , P. Hu , P. Hughes , Surf. Sci. 2016, 646, 45–49.

[15] B. Yang , R. Burch , C. Hardacre , G. Headdock , P. Hu , J. Catal. 2013, 305, 264–276.

[16] J. Liu , J. Sun , T. Singh , S. Lin , L. Ma , Green Chem. Eng. 2022, 3, 395–404.

[17] X. Ge , Z. Ren , Y. Cao , X. Liu , J. Zhang , G. Qian , X. Gong , L. Chen , X. Zhou , W. Yuan , X. Duan , J. Mater. Chem. A 2022, 10, 19722–19731.

[18] G. Radivoy , E. Buxaderas , M. Volpe , Synthesis 2018, 51, 1466–1472.

[19] Q. Wang , J. Zhao , L. Xu , L. Yu , Z. Yao , G. Lan , L. Guo , J. Zhao , C. Lu , Z. Pan , J. Wang , Q. Zhang , X. Li , Appl. Surf. Sci. 2021, 562.

[20] B.‐H. Zhao , F. Chen , M. Wang , C. Cheng , Y. Wu , C. Liu , Y. Yu , B. Zhang , Sustain 2023, 6, 827–837.

[21] G. Kastlunger , P. Lindgren , A. A. Peterson , J. Phys. Chem. C. 2018, 122, 12771–12781.

[22] M. M. Melander , M. J. Kuisma , T. E. K. Christensen , K. Honkala , J. Chem. Phys. 2019, 150, 041706. 30709274 10.1063/1.5047829

[23] K. R. Chan , J. K. Norskov , J. Phys. Chem. Lett. 2015, 6, 2663–2668. 26266844 10.1021/acs.jpclett.5b01043

[24] C. S. Chen , J. H. Wan , B. S. Yeo , J. Phys. Chem. C 2015, 119, 26875–26882.

[25] I. V. Yudanov , R. Sahnoun , K. M. Neyman , N. Rösch , J. Hoffmann , S. Schauermann , V. Johánek , H. Unterhalt , G. Rupprechter , J. Libuda , H.‐J. Freund , J. Phys. Chem. B 2002, 107, 255–264.

[26] G. Kresse , J. Furthmuller , Phys. Rev. B: Condens. Matter 1996, 54, 11169–11186. 9984901 10.1103/physrevb.54.11169

[27] G. Kresse , J. Hafner , Phys. Rev. B: Condens. Matter 1993, 47, 558–561. 10004490 10.1103/physrevb.47.558

[28] S. Grimme , J. Antony , S. Ehrlich , H. Krieg , J. Chem. Phys. 2010, 132, 154104. 20423165 10.1063/1.3382344

[29] Z. P. Liu , P. Hu , J. Am. Chem. Soc. 2003, 125, 1958–1967. 12580623 10.1021/ja0207551

[30] J. K. Norskov , J. Rossmeisl , A. Logadottir , L. Lindqvist , J. R. Kitchin , T. Bligaard , H. Jonsson , J. Phys. Chem. B 2004, 108, 17886–17892. 39682080 10.1021/jp047349j

[31] J. Chen , M. Jia , P. Hu , H. Wang , J. Comput. Chem. 2021, 42, 379–391. 33315262 10.1002/jcc.26464

[32] J. Chen , M. Jia , Z. Lai , P. Hu , H. Wang , J. Chem. Phys. 2021, 154, 024108. 33445900 10.1063/5.0032228

[33] J.‐F. Chen , Y. Mao , H.‐F. Wang , P. Hu , ACS Catal. 2016, 6, 7078–7087.

[34] L. Wang , S. A. Nitopi , E. Bertheussen , M. Orazov , C. G. Morales‐Guio , X. Liu , D. C. Higgins , K. Chan , J. K. Nørskov , C. Hahn , T. F. Jaramillo , ACS Catal. 2018, 8, 7445–7454.

[35] J. P. Perdew , K. Burke , M. Ernzerhof , Phys. Rev. Lett. 1996, 77, 3865–3868. 10062328 10.1103/PhysRevLett.77.3865

[36] G. Kresse , D. Joubert , Phys. Rev. B: Condens. Matter 1999, 59, 1758–1775.

[37] L. Bengtsson , Phys. Rev. B: Condens. Matter 1999, 59, 12301–12304.

[38] H. J. Monkhorst , J. D. Pack , Phys. Rev. B: Condens. Matter 1976, 13, 5188–5192.

[39] K. Mathew , R. Sundararaman , K. Letchworth‐Weaver , T. A. Arias , R. G. Hennig , J. Chem. Phys. 2014, 140, 084106. 24588147 10.1063/1.4865107

[40] K. Mathew , V. S. C. Kolluru , S. Mula , S. N. Steinmann , R. G. Hennig , J. Chem. Phys. 2019, 151, 234101. 31864239 10.1063/1.5132354

[41] H. Ogasawara , B. Brena , D. Nordlund , M. Nyberg , A. Pelmenschikov , L. G. Pettersson , A. Nilsson , Phys. Rev. Lett. 2002, 89, 276102. 12513221 10.1103/PhysRevLett.89.276102

[42] S. Goedecker , J. Chem. Phys. 2004, 120, 9911–9917. 15268009 10.1063/1.1724816

[43] J. Rossmeisl , E. Skúlason , M. E. Björketun , V. Tripkovic , J. K. Nørskov , Chem. Phys. Lett. 2008, 466, 68–71.

[44] S. Ringe , C. G. Morales‐Guio , L. D. Chen , M. Fields , T. F. Jaramillo , C. Hahn , K. Chan , Nat. Commun. 2020, 11, 33. 31911585 10.1038/s41467-019-13777-z PMC6946669

[45] W. L. Holstein , M. Boudart , J. Phys. Chem. B 1997, 101, 9991–9994.

[46] C. T. Campbell , ACS Catal. 2017, 7, 2770–2779.

[47] A. Michaelides , Z. P. Liu , C. J. Zhang , A. Alavi , D. A. King , P. Hu , J. Am. Chem. Soc. 2003, 125, 3704–3705. 12656593 10.1021/ja027366r

[48] A. Alavi , P. Hu , T. Deutsch , P. L. Silvestrelli , J. Hutter , Phys. Rev. Lett. 1998, 80, 3650–3653.

[49] R. Sundararaman , K. Letchworth‐Weaver , K. A. Schwarz , D. Gunceler , Y. Ozhabes , T. A. Arias , SoftwareX 2017, 6, 278–284. 29892692 10.1016/j.softx.2017.10.006 PMC5992620

[50] R. Sundararaman , W. A. Goddard, 3rd , J. Chem. Phys. 2015, 142, 064107. 25681887 10.1063/1.4907731

[51] Y. Wang , Z. Wang , C.‐T. Dinh , J. Li , A. Ozden , M. Golam Kibria , A. Seifitokaldani , C.‐S. Tan , C. M. Gabardo , M. Luo , H. Zhou , F. Li , Y. Lum , C. McCallum , Y. Xu , M. Liu , A. Proppe , A. Johnston , P. Todorovic , T.‐T. Zhuang , D. Sinton , S. O. Kelley , E. H. Sargent , Catal 2019, 3, 98–106.

