# Supplementary material for: Tuning Eley–Rideal Mechanism in Electrochemical Acetylene Semi‐Hydrogenation
Source: Angew Chem Int Ed Engl. 2025 Jul 4;64(35):e202512218. doi: 10.1002/anie.202512218 (PMC12377425; doi:10.1002/anie.202512218)
Supplement: Supplementary file 1 — Supporting Information [file ANIE-64-e202512218-s001.docx]

**Supporting Information**

**Tuning Eley-Rideal Mechanism in Electrochemical Acetylene Semi-hydrogenation**

Chengyi Zhang^[a],+^, Jiguang Zhang^[b],+^, Zihao Jiao^[a]^, Yanwei Lum^[b]^*, and Ziyun Wang^[a]^*

[a] School of Chemical Sciences, University of Auckland, Auckland, New Zealand

[b] Department of Chemical and Biomolecular Engineering, National University of Singapore, Singapore, Republic of Singapore

^+^These authors contributed equally to this work

Correspondence: [lumyw@nus.edu.sg](mailto:lumyw@nus.edu.sg)

Correspondence: [ziyun.wang@auckland.ac.nz](mailto:ziyun.wang@auckland.ac.nz)

**Contents**

[1. Theoretical Sections 3](#_Toc199410105)

[1.1 Density Functional Theory Calculations 3](#_Toc199410106)

[1.2 Electrochemical Barrier Calculations 5](#_Toc199410107)

[1.3 Microkinetic modeling 6](#_Toc199410108)

[2. Experiment Methods 8](#_Toc199410109)

[2.1 Chemicals 8](#_Toc199410110)

[2.2 Preparation of Electrodes 8](#_Toc199410111)

[2.3 Materials Characterization 8](#_Toc199410112)

[2.4 Electrochemical Measurements 8](#_Toc199410113)

[3. Supporting Tables and Figures 10](#_Toc199410114)

[4. Supporting References 53](#_Toc199410115)

# 1. Theoretical Sections

## 1.1 Density Functional Theory Calculations

All density functional theory (DFT) calculations were performed using the Vienna Ab-initio Simulation Package (VASP).^[1-2]^ The plane-wave basis set was employed with a kinetic energy cutoff of 450 eV, ensuring convergence of total energies. The exchange-correlation interactions were described using the Perdew–Burke–Ernzerhof (PBE) functional within the generalized gradient approximation (GGA).^[3]^ All symmetry operations were explicitly turned off to allow full structural relaxation without constraints. For the electronic smearing, Methfessel-Paxton first-order smearing was adopted with a width of 0.2 eV, suitable for metallic systems. The electronic self-consistency loop was considered converged when the total energy change between iterations fell below 10⁻^7^ eV*,* with at least 5 and at most 60 SCF iterations performed per ionic step. The electronic minimization was handled using the fast Davidson iteration scheme, and real-space projection operators were automatically selected to improve computational efficiency. Medium precision was used for the FFT grid, and Grimme’s DFT-D3 dispersion corrections were included to account for van der Waals interactions.^[4]^ The Brillouin zone was sampled using a Γ-centered Monkhorst-Pack grid of 3 × 3 × 1, which was sufficient to converge the total energy for the chosen surface model.^[5]^ Our calculations have taken the spin-polarized effect into account. Ni, and Fe magma were tested individually in primitive cells to obtain a stable structure. In our study, we intentionally selected a series of metals (Cu, Fe, Ni, Ag, Au) and their Cu-based alloys to span a broad range of metal electronegativities, from low (Fe: 1.83) to high (Au: 2.54), with Cu (1.90) as the central element. Besides, all these metals all adopt a face-centered cubic (fcc) structure, with the (111) facet being the most stable. This consistent crystallographic orientation helps minimize structural variability, allowing us to focus on the role of electronegativity in tuning the ER mechanism. This design allows us to systematically investigate how electronic properties, especially electronegativity, influence the binding strength of C_2_H_2_ and the associated catalytic behaviour under the Eley–Rideal mechanism. Specifically, Fe and Ni were chosen not to replicate traditional Pt/Pd-based catalysts, but to extend the range of metal–adsorbate interaction strength, enabling us to construct a more comprehensive descriptor–activity relationship, including both the strong-binding and weak-binding regimes. Pt and Pd (with electronegativities of 2.28 and 2.20, respectively) fall within a relatively narrow range similar to Cu/Ag/Au, and including them would not significantly increase the spread of electronic descriptors in our model. The hydrogenation of acetylene on various metal surfaces typically proceeds via a PCET mechanism, in which the proton and electron are transferred simultaneously. This type of reaction is a prototypical case where the computational hydrogen electrode (CHE) model can be reliably applied. In PCET steps, the free energy of the transition state and final state varies approximately linearly with the applied electrode potential, allowing the CHE approach to capture potential-dependent energy profiles without the need for explicit charge control.^[6-9]^ Therefore, the inclusion of Fe and Ni is critical for testing the robustness and generality of our theoretical model across a wider electronegativity and adsorption energy spectrum, which is essential for understanding and predicting selective hydrogenation behaviours beyond the well-established noble-metal systems. To account for spin polarization, we first performed spin-polarized calculations on Ni and Fe in their respective primitive cells, yielding magnetic moments of 1.2 μB for Ni and 2.2 μB for Fe. These values were subsequently used as initial spin configurations for constructing the adsorption structures involving the two metals.

All the adsorption geometries were optimized using a force-based conjugate gradient algorithm, while transition states (TSs) were located with a constrained minimization technique.^[10-12]^ To mimic realistic surface conditions while minimizing computational cost, the bottom two atomic layers were fixed, and only the top two layers were allowed to relax. This partial relaxation approach is commonly employed in surface calculations to reduce spurious slab polarization and simulate the semi-infinite bulk beneath the surface. The vacuum layer was set sufficiently large (typically >15 Å) to prevent spurious interactions along the normal surface between periodic images. Initial charge densities were generated by atomic superposition*.*

Geometry optimizations were performed using a conjugated gradient algorithm with a time step of 0.2 fs. The force convergence criterion was set to 0.05 eV/Å. The stress tensor was computed, but only atomic positions (not cell shape or volume) were allowed to relax. For the geometry optimization, implicit solvation effects were included using the VASPSOL model, with a surface tension-related cavity energy term set to 80 to express the aqueous environment.^[13-14]^ For the explicit solvation structure, water densities were chosen to be close to that of the Pt(111) bilayer structure found in UHV experiments within the unit cell sizes considered.^[15]^ Water layer structures were determined using a minima-hopping algorithm that alternates between [Molecular Dynamics](https://www.sciencedirect.com/topics/chemistry/molecular-dynamics) and [geometry optimization](https://www.sciencedirect.com/topics/engineering/geometry-optimization) steps to construct a series of [local minima](https://www.sciencedirect.com/topics/engineering/local-minimum).^[16]^ To simulate a charged double layer at the electrochemical interface, a single [hydrogen atom](https://www.sciencedirect.com/topics/earth-and-planetary-sciences/hydrogen-atom) was placed in the water layer. The ground-state electronic structure redistributes the charge from this atom's one electron to the metal, creating a charge-separated double layer.^[17]^ We obtained the initial complete solvent–water layer structure from our previous work and applied the minimal hopping algorithms to find the stable structures.^[18]^ All reaction-free energies were calculated based on DFT electronic energies corrected with zero-point energy (ZPE) and entropy contributions at 298.15 K. For each intermediate and transition state, vibrational frequency calculations were performed within the harmonic approximation using finite displacements of adsorbed atoms. The ZPE for each species was obtained by summing the zero-point contributions from all vibrational modes:

$$ZPE=\frac{1}{2}\sum_{i} hv_{i}$$

where $v_{i}$​ is the frequency of the i-th normal mode. Imaginary frequencies (if any) associated with transition states were excluded from this summation. The GC-DFT calculations were performed in the JDFTx,^[19]^ we adopted the optimized structure from the VASP package and calculated the single-point energy under different potentials to test the reliability of our solvation model. The Brillouin zone was sampled using a uniform 3×3×1 Gamma-centered k-point mesh, suitable for surface slab geometries. Ultrasoft pseudopotentials from the GBRV library were employed for all elements. The Perdew-Burke-Ernzerhof (PBE) generalized gradient approximation was used for exchange-correlation interactions. A plane-wave kinetic energy cutoff of 20 Hartrees was applied. Electronic minimization was performed with a convergence threshold of 1×10⁻5 Hartree and up to 1000 iterations. Finite-temperature Fermi smearing of 0.1 eV was used. Implicit solvation was modeled using the CANDLE variant of the LinearPCM method with water as the solvent, and ionic concentrations of 0.1 M K⁺ and F⁻ were included.^[20]^13 A target electron chemical potential was set, and van der Waals interactions were included. All calculations were spin-polarized along the z-axis.

## 1.2 Electrochemical Barrier Calculations

We utilized the computational hydrogen electrode model to calculate reaction energies as a function of potential. At a potential of U=0 V versus RHE, protons and electrons are in equilibrium with hydrogen gas (H_2_) under standard conditions applicable across all pH levels:^[21]^

$$H^{+}+e^{-}\to H_{2(g)}$$

At a given U≠ 0V versus RHE,

$$\mu\left( H^{+} \right)+\mu\left( e^{-} \right)=\frac{1}{2}\mu\left( H_{2} \right)-eU$$

for calculating the Gibbs energy change of the energy barrier*.* To evaluate the effect of applied potential on proton-coupled electron transfer (PCET) steps, we employed a symmetry factor (β) of 0.5.^[22]^ This value reflects the assumption that the transition state involves the transfer of half an electron, which is commonly adopted in electrochemical kinetics when the charge transfer is concerted but the exact charge distribution at the transition state is unknown. Accordingly, the potential-dependent correction to the reaction barrier ($\Delta G^{\ddagger}(U)$) was introduced as:

$$\Delta G^{\ddagger}(U)=\Delta G^{\ddagger}(0)-\beta eU$$

where $\Delta G^{\ddagger}(0)$ denotes the activation barrier at the reference potential (typically 0 V vs. RHE), e is the elementary charge, and U is the applied electrode potential. This correction effectively accounts for the electrostatic stabilization of the transition state in response to increasing overpotential. The use of β = 0.5 ensures a balanced treatment of the potential influence, consistent with prior theoretical treatments of PCET processes in electrocatalysis. To further confirm the reliability of the CHE model in our system, we adopted the hydrogenation of *HCCH on Cu on different potential vs. SHE in Figure S85. Our results confirm the linear change of the hydrogenation barrier with potential, indicating the reliability of our model. In addition, in microkinetic modeling, we directly represent the influence of pH on the overall reaction kinetics through the concentration of proton donors or acceptors (e.g., H⁺), which are modulated in accordance with the pH value.

## 1.3 Microkinetic modeling

In microkinetic modeling, the reaction network is constructed from a series of elementary steps, and fundamental thermodynamic and kinetic principles govern the net rate of each step. According to the De Donder relation, the net rate $r_{i}$​ for an elementary step i can be expressed as a product of the forward rate constant $k_{i}$​. The surface coverages $\theta_{j}$​ of the participating reactants, their stoichiometric coefficients $v_{ij}$​, and a reversibility factor (1−$Z_{i}$), where $Z_{i}$​ represents the thermodynamic driving force for reversibility.^[23]^ Mathematically, this is written as:

$$r_{i}=k_{i}\prod_{j} \theta(j)^{\nu_{ij}}\left( 1-Z_{i} \right)$$

Here, $Z_{i}=\prod_{j} \theta(j)^{\nu_{ij}}/K_{\text{eq },i}$, where $K_{\text{eq },i}$is the equilibrium constant of step i, given by

$$K_{\text{eq },i}=exp(-\Delta G_{i}/RT)$$

This term reflects the extent to which the step is reversible:$Z_{i}$→0 for irreversible steps and $Z_{i}$→1 for steps approaching equilibrium. The stoichiometric coefficient $v_{ij}$​ indicates the number of molecules of species j involved in step i.

To determine which elementary step most strongly influences the overall reaction rate, we employ the **degree of rate control (DRC)**, a sensitivity metric introduced by Campbell and co-workers.^[24]^ The DRC for a transition state i quantifies the relative change in the overall reaction rate r concerning a perturbation in the Gibbs free energy of that transition state, while holding the energies of all other species constant. It is formally defined as:

$$\mathrm{DRC}_{i}=\frac{\partial\ln r}{\partial\left( -G_{i}^{\ddagger}/RT \right)}$$

This metric captures how much the rate-limiting character of a particular transition state contributes to the net rate, allowing mechanistic insight beyond conventional energy barrier comparisons. A larger DRC value indicates greater control over the overall reaction rate by that particular transition state.

Similarly, the importance of the thermodynamic stability of an intermediate species n can be evaluated through a corresponding DRC definition:

$$\mathrm{DRC}_{n}=\frac{\partial\ln r}{\partial\left( -G_{n}/RT \right)}$$

This formulation identifies how changes in an intermediate's standard Gibbs free energy affect the overall reaction rate, assuming all other species remain unchanged. These DRC values provide a rigorous way to dissect the interplay between kinetics and thermodynamics across complex catalytic networks. We adopted the CATKINAS for microkinetic modeling solver.^[25-27]^

In terms of input conditions, the model operates under room temperature. The products were set to be 10^-20^ bars, to describe the reaction condition at the beginning. Mass transport limitations were not included in the modeling. The proton concentration was limited by setting the maximal proton concentration near the surface to 10^-pH^.

# 2. Experiment Methods

## 2.1 Chemicals

Potassium hydroxide (≥95%) was sourced from Sigma-Aldrich. Hydrophobic porous polytetrafluoroethylene (PTFE) substrates with a pore size of 0.45 μm were supplied by Beijing Zhongxingweiye Instrument Co., Ltd. Deionized water (18.2 MΩ) was produced using an OmniaPure UltraPure Water System (Stakpure GmbH). High-purity targets of Cu (99.999%), Ni (99.999%), Ag (99.999%), and Au (99.999%) were procured from the Kurt J. Lesker Company. Acetylene gas (99.9%) and calibration gas mixtures for gas chromatography were obtained from Air Liquide Singapore Pte. Ltd. The Nafion 117 proton exchange membrane, Fumasep FAA-3-PK-130 anion exchange membrane, and titanium screen mesh were purchased from FuelCellStore. The electrochemical flow cell and Hg/HgO reference electrodes were provided by Tianjin Aida Hengsheng Technology Development Co. All chemicals used in the study were of analytical grade and utilized without additional purification.

## 2.2 Preparation of Electrodes

The working electrodes were prepared using magnetron sputtering. The sputtering power was controlled to fix the total deposition rate at 2 Å/s. The sputtering power was set as shown in Table 2. The IrO_x_-coated Ti mesh electrode was prepared using a thermal decomposition method, following the methodology described by Luc et al.^[28]^

## 2.3 Materials Characterization

SEM images and EDX measurements were obtained using a JSM-7610FPlus.

## 2.4 Electrochemical Measurements

All electrochemical measurements in this work were conducted using an Autolab PGSTAT204 potentiostat. C_2_H_2_ reduction experiments were carried out in a custom-designed electrochemical cell. In this setup, the working and counter electrodes were restricted to an area of 1 cm² and positioned parallel. When using alkaline or neutral electrolytes, anion exchange membrane was employed to separate the cathode and anode chambers. Bulk electrolysis of C_2_H_2_ was performed using chronopotentiometry for a minimum of 0.5 hours per sample. C_2_H_2_ gas was continuously fed into the gas chamber of the electrochemical cell at a flow rate of 20 sccm. Gas products were analyzed using a gas chromatograph with a thermal conductivity detector and a flame ionization detector (Agilent 8860 GC). An Hg/HgO electrode served as the reference electrode, and solution resistance was measured using potentiostatic electrochemical impedance spectroscopy over a frequency range of 100,000 Hz to 0.1 Hz.

# 3. Supporting Tables and Figures


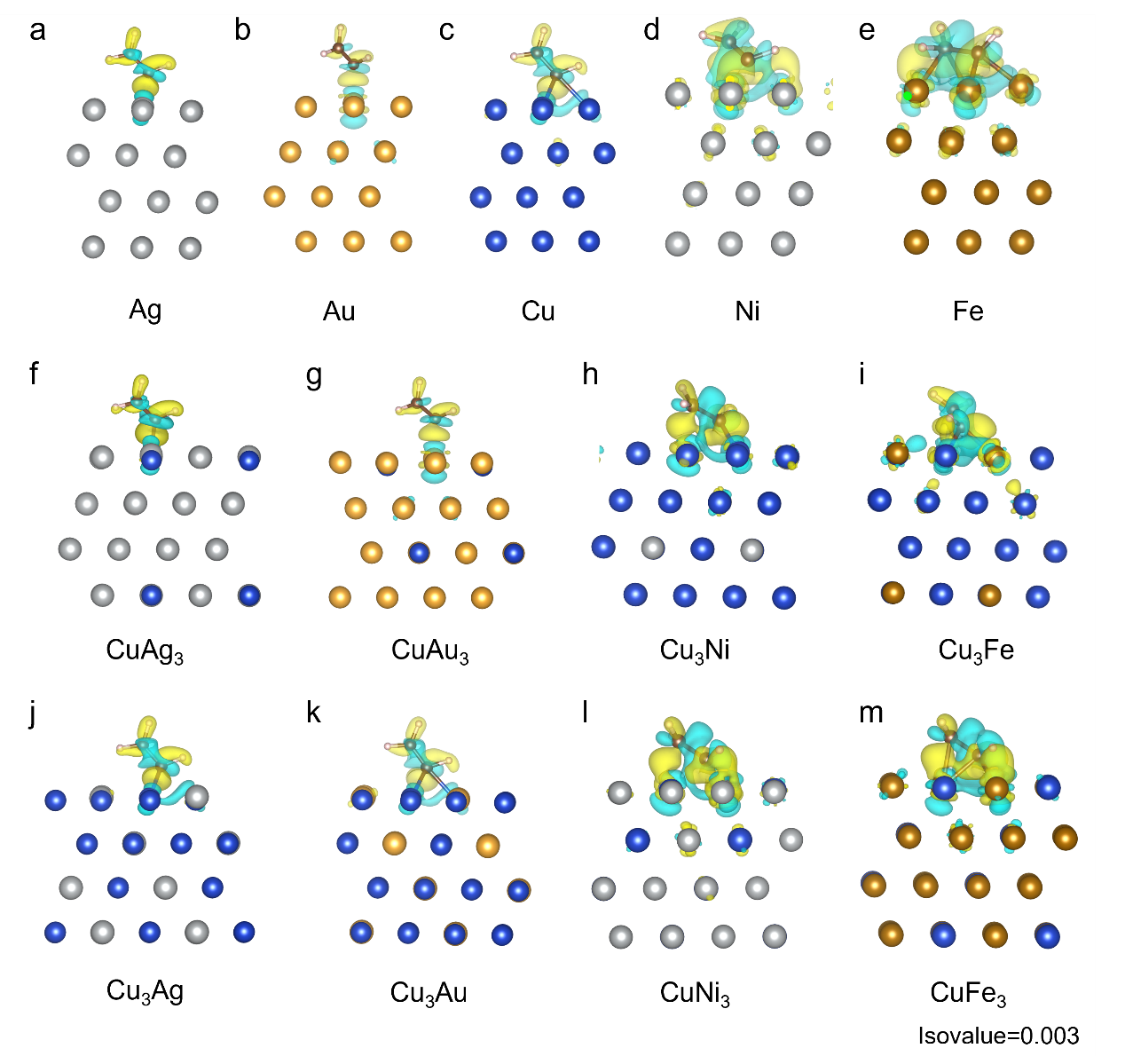


**Figure S1.** The charge density difference of *HCCH_2_ on the different slabs.


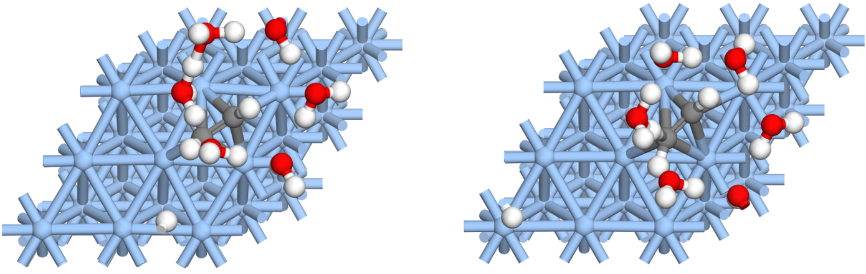


**Figure S2.** The molecular configuration of the initial and transition state of the hydrogenation on *HCCH on Ag in ER mechanism.


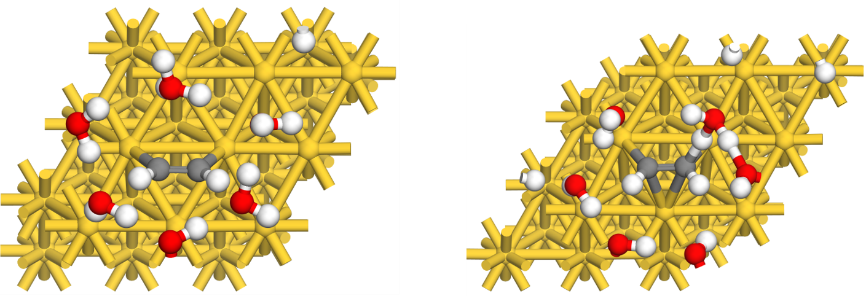


**Figure S3.** The molecular configuration of the initial and transition state of the hydrogenation on *HCCH on Au in ER mechanism.


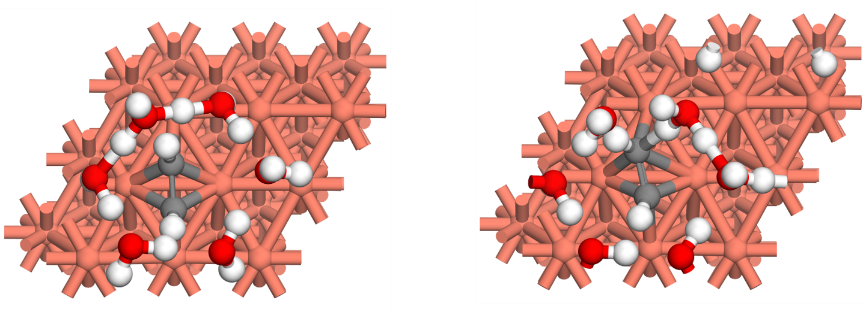


**Figure S4.** The molecular configuration of the initial and transition state of the hydrogenation on *HCCH on Cu in ER mechanism.


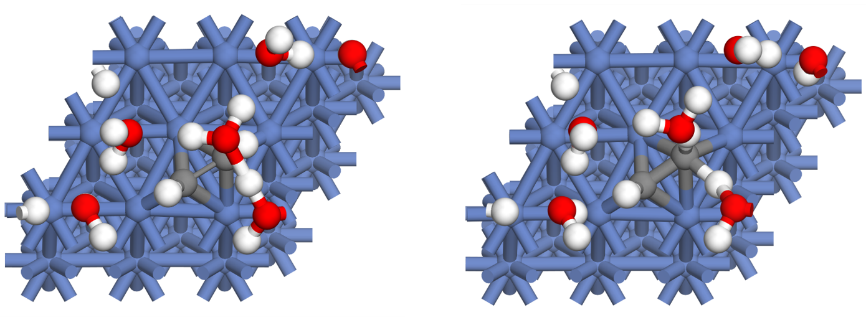


**Figure S5.** The molecular configuration of the initial and transition state of the hydrogenation on *HCCH on Ni in ER mechanism.


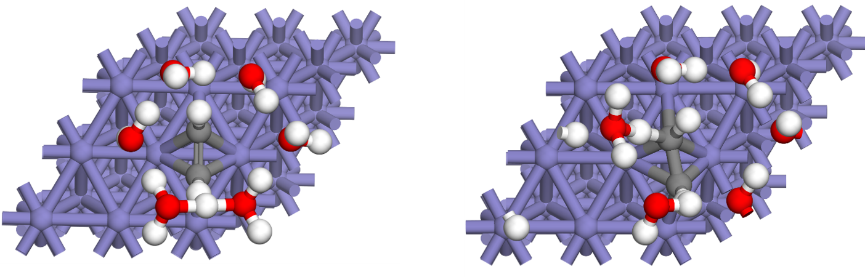


**Figure S6.** The molecular configuration of the initial and transition state of the hydrogenation on *HCCH on Fe in ER mechanism.


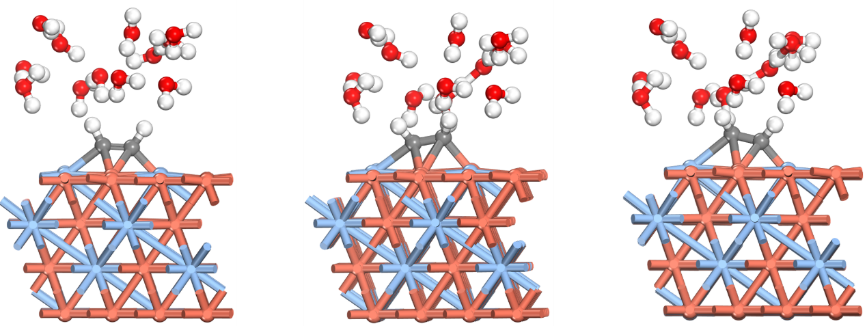


**Figure S7.** The molecular configuration of the initial and transition state of the hydrogenation on *HCCH on different carbon on Cu_3_Ag in ER mechanism.


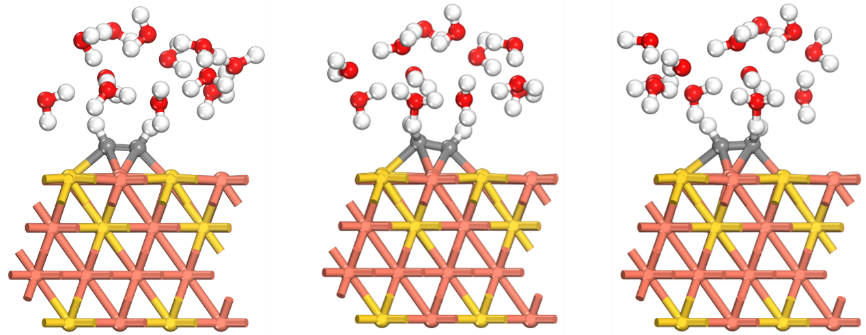


**Figure S8.** The molecular configuration of the initial and transition state of the hydrogenation on *HCCH on different carbon on Cu_3_Au in ER mechanism.


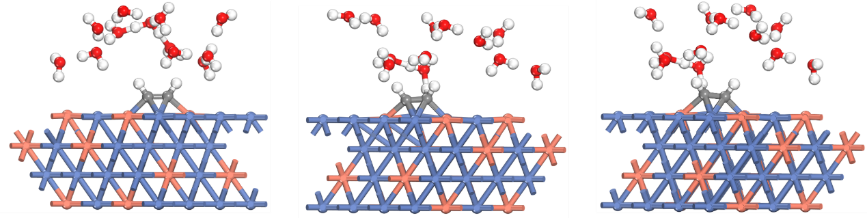


**Figure S9.** The molecular configuration of the initial and transition state of the hydrogenation on *HCCH on different carbon on CuNi_3_ in ER mechanism.


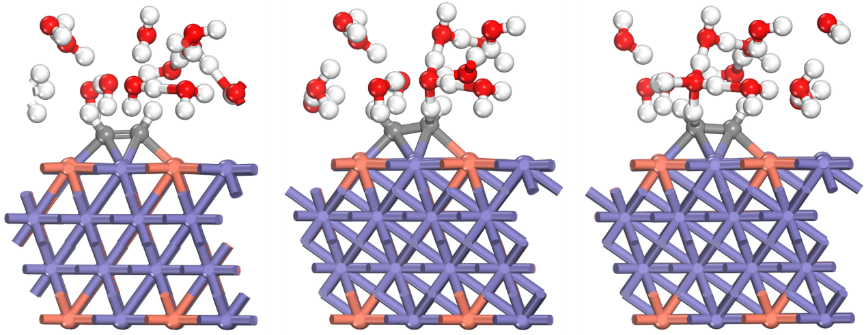


**Figure S10.** The molecular configuration of the initial and transition state of the hydrogenation on *HCCH on different carbon on CuFe_3_ in ER mechanism.


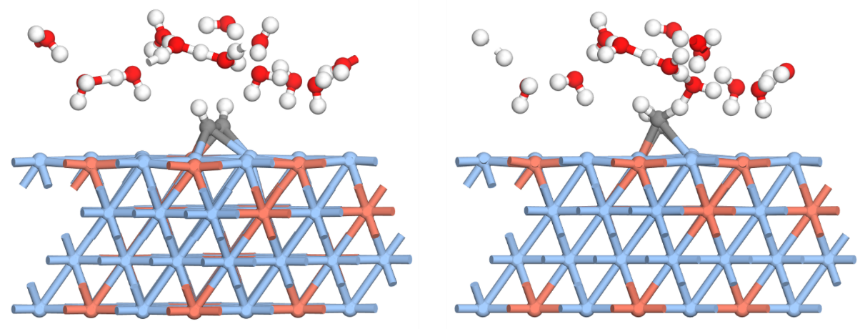


**Figure S11.** The molecular configuration of the initial and transition state of the hydrogenation on *HCCH on CuAg_3_ in ER mechanism.


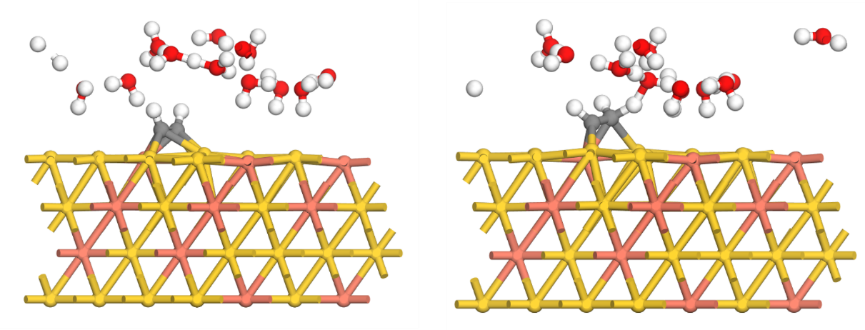


**Figure S12.** The molecular configuration of the initial and transition state of the hydrogenation on *HCCH on CuAu_3_ in ER mechanism.


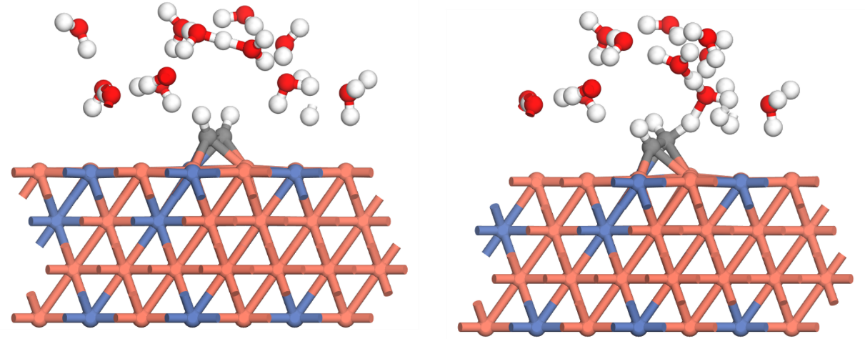


**Figure S13.** The molecular configuration of the initial and transition state of the hydrogenation on *HCCH on Cu_3_Ni in ER mechanism.


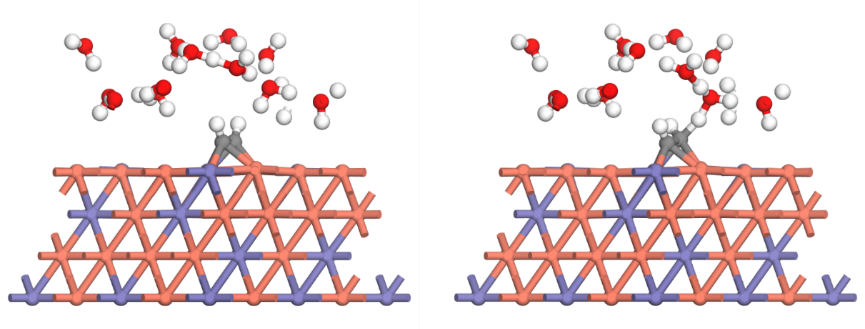


**Figure S14.** The molecular configuration of the initial and transition state of the hydrogenation on *HCCH on Cu_3_Fe in ER mechanism.


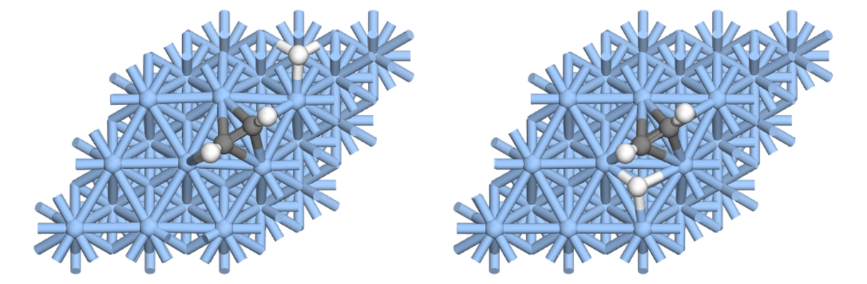


**Figure S15.** The molecular configuration of the initial and transition state of the hydrogenation on *HCCH on Ag in the LH mechanism, we hide the water molecules to make the configuration more straightforward.


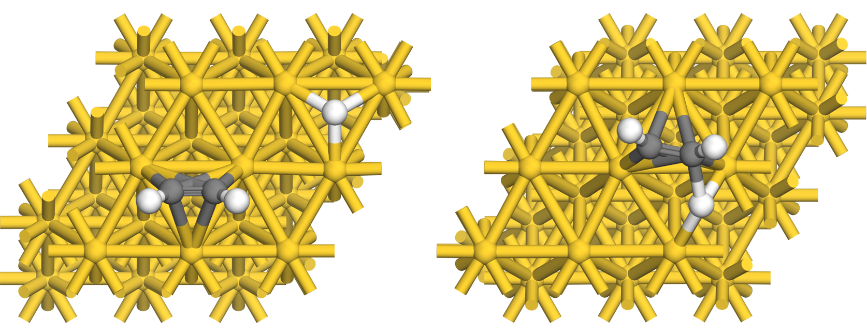


**Figure S16.** The molecular configuration of the initial and transition state of the hydrogenation on *HCCH on Au in the LH mechanism, we hide the water molecules to make the configuration more straightforward.


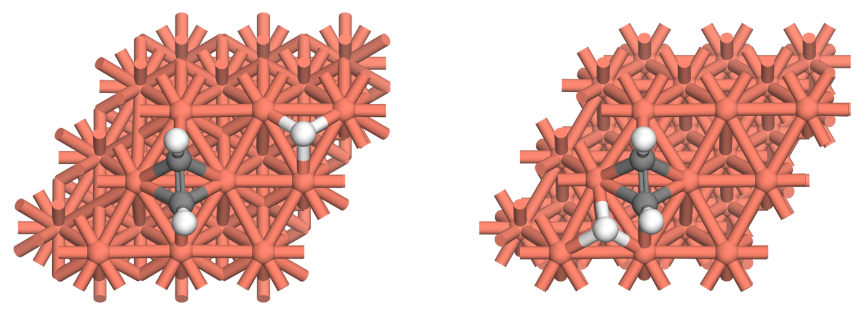


**Figure S17.** The molecular configuration of the initial and transition state of the hydrogenation on *HCCH on Cu in the LH mechanism, we hide the water molecules to make the configuration more straightforward.


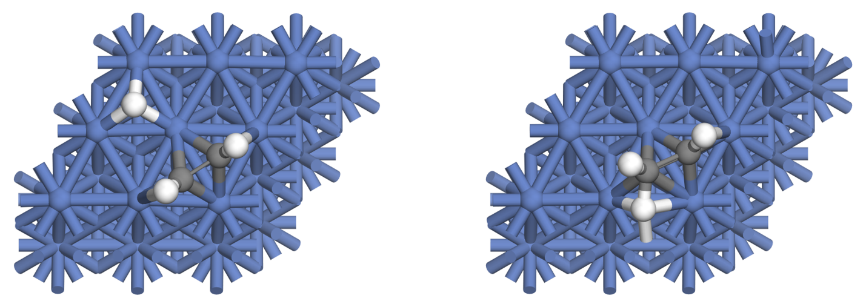


**Figure S18.** The molecular configuration of the initial and transition state of the hydrogenation on *HCCH on Ni in the LH mechanism, we hide the water molecules to make the configuration more straightforward.


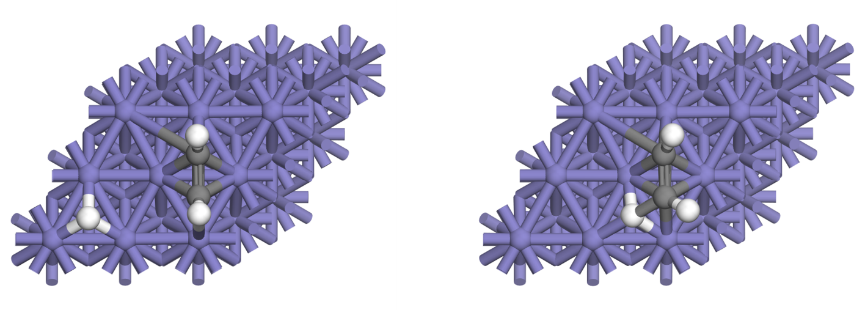


**Figure S19.** The molecular configuration of the initial state and transition state of the hydrogenation on *HCCH on Fe in the LH mechanism, we hide the water molecules to make the configuration more straightforward.


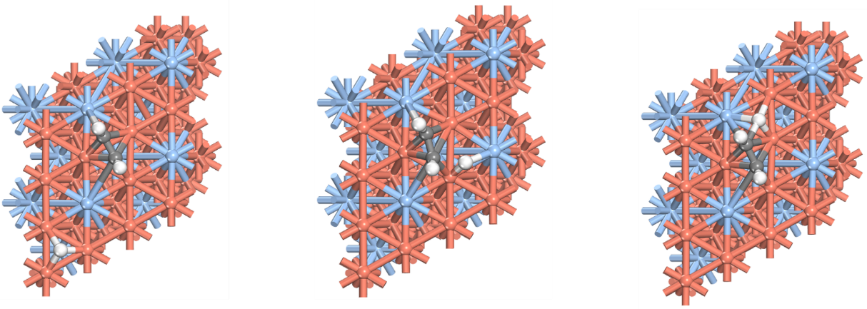


**Figure S20.** The molecular configuration of the initial and transition state of the hydrogenation on *HCCH on different carbon on Cu_3_Ag in the LH mechanism, we hide the water molecules to make the configuration more straightforward.


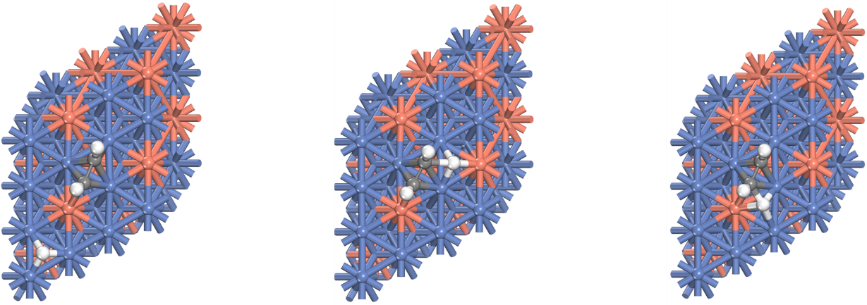


**Figure S21.** The molecular configuration of the initial and transition state of the hydrogenation on *HCCH on different carbon on CuNi_3_ in the LH mechanism, we hide the water molecules to make the configuration more straightforward.


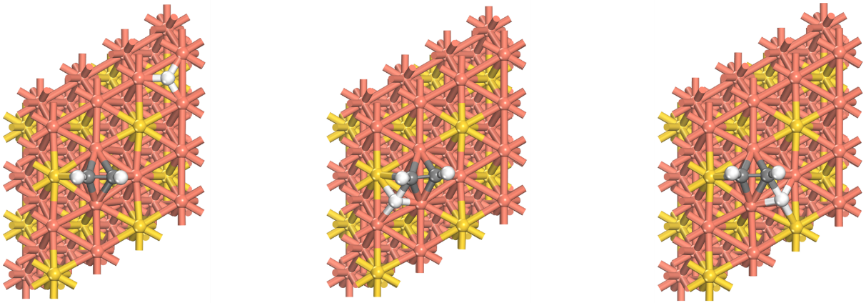


**Figure S22.** The molecular configuration of the initial and transition state of the hydrogenation on *HCCH on different carbon on Cu_3_Au in the LH mechanism, we hide the water molecules to make the configuration more straightforward.


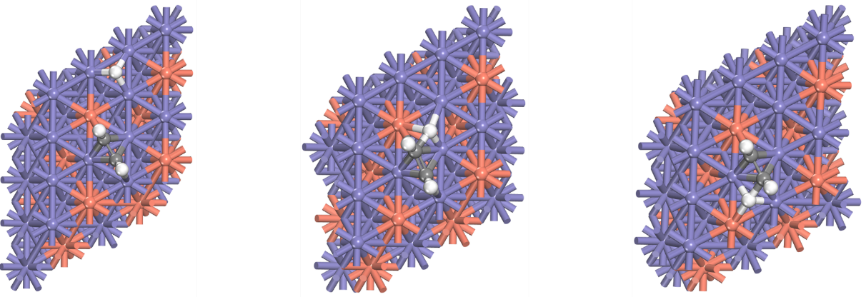


**Figure S23.** The molecular configuration of the initial and transition state of the hydrogenation on *HCCH on different carbon on CuFe_3_ in the LH mechanism, we hide the water molecules to make the configuration more straightforward.


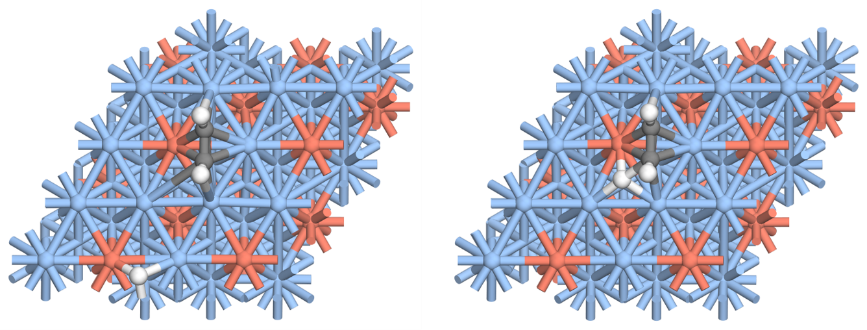


**Figure S24.** The molecular configuration of the initial and transition state of the hydrogenation on *HCCH on CuAg_3_ in LH mechanism, we hide the water molecules to make the configuration clearer.


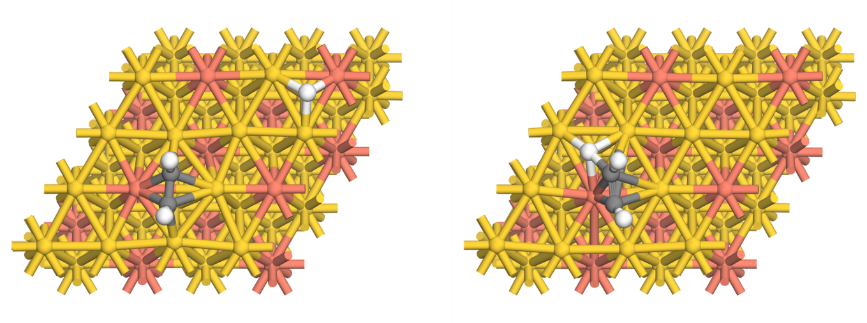


**Figure S25.** The molecular configuration of the initial and transition state of the hydrogenation on *HCCH on CuAu_3_ in the LH mechanism, we hide the water molecules to make the configuration more straightforward.


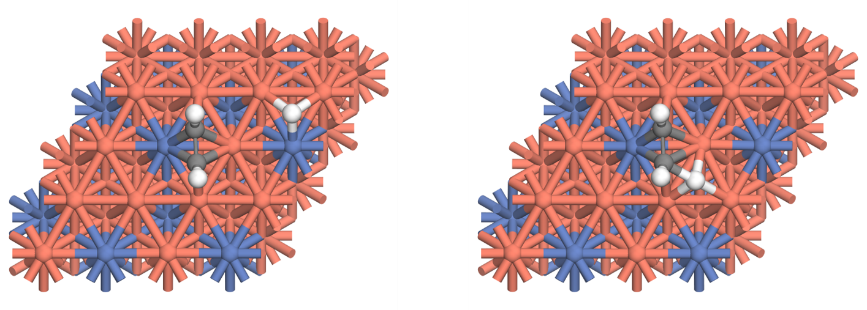


**Figure S26.** The molecular configuration of the initial and transition state of the hydrogenation on *HCCH on Cu_3_Ni in the LH mechanism, we hide the water molecules to make the configuration more straightforward.


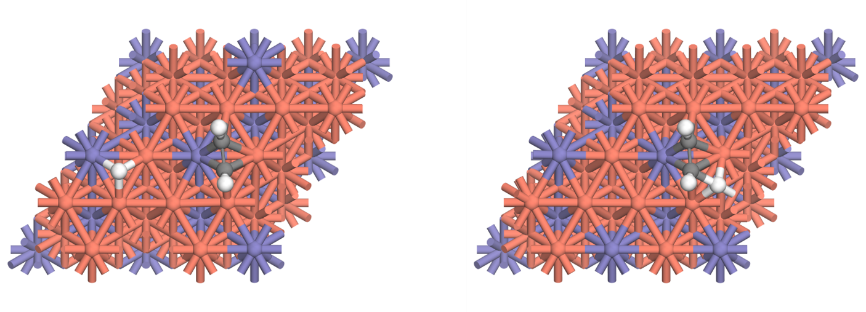


**Figure S27.** The molecular configuration of the initial and transition state of the hydrogenation on *HCCH on Cu_3_Fe in the LH mechanism, we hide the water molecules to make the configuration more straightforward.


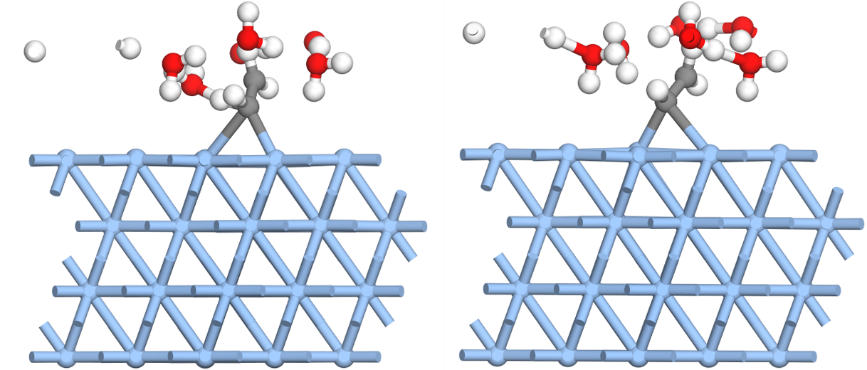


**Figure S28.** The molecular configuration of the initial and transition state of the hydrogenation on *HCCH_2_ on Ag in ER mechanism.


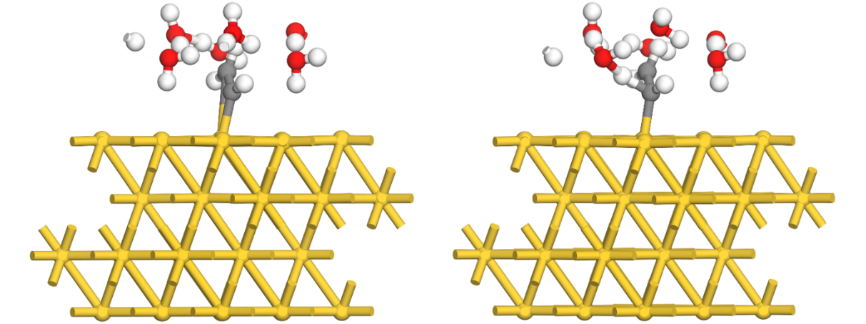


**Figure S29.** The molecular configuration of the initial and transition state of the hydrogenation on *HCCH_2_ on Au in ER mechanism.


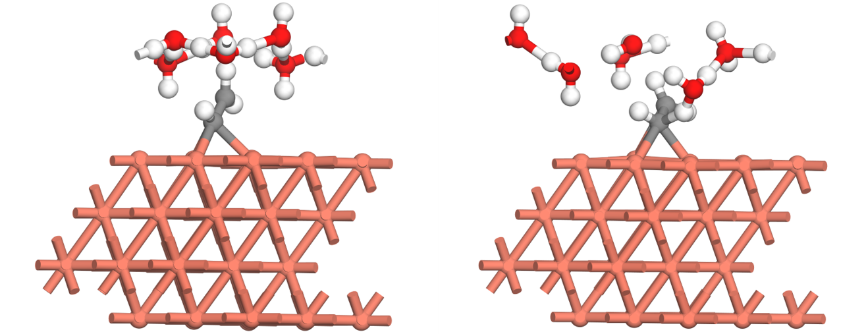


**Figure S30.** The molecular configuration of the initial and transition states of the hydrogenation on *HCCH_2_ on Cu in ER mechanism.


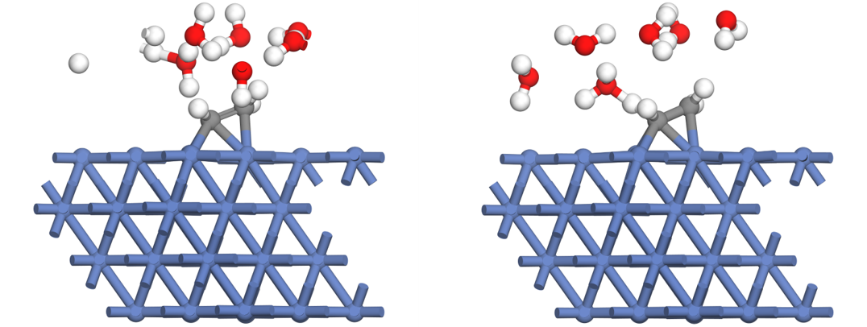


**Figure S31.** The molecular configuration of the initial and transition state of the hydrogenation on *HCCH_2_ on different carbon on Ni in ER mechanism.


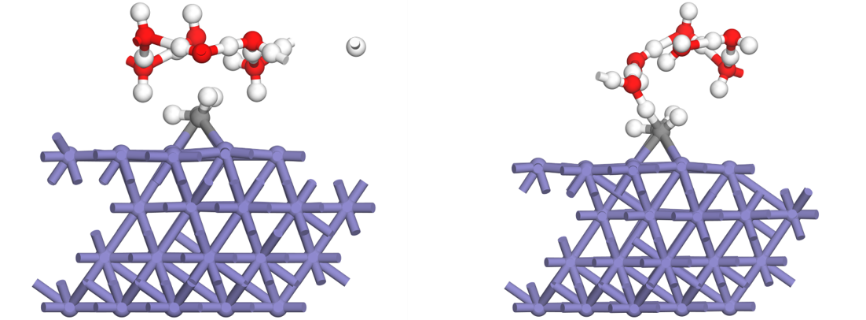


**Figure S32.** The molecular configuration of the initial and transition state of the hydrogenation on *HCCH_2_ on Fe in ER mechanism.


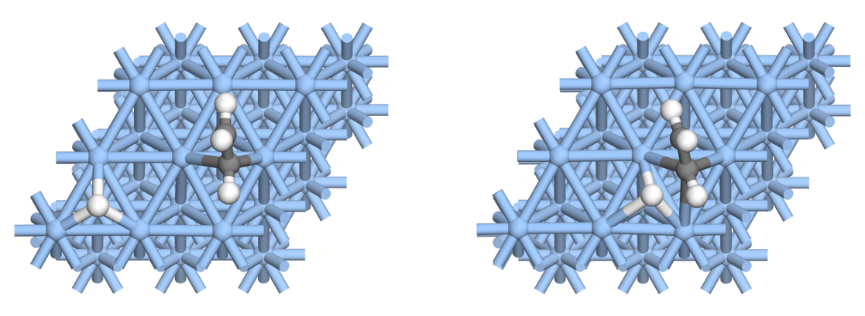


**Figure S33.** The molecular configuration of the initial state and transition state of the hydrogenation on *HCCH_2_ on different carbon on Ag in the LH mechanism, we hide the water molecules to make the configuration more straightforward.


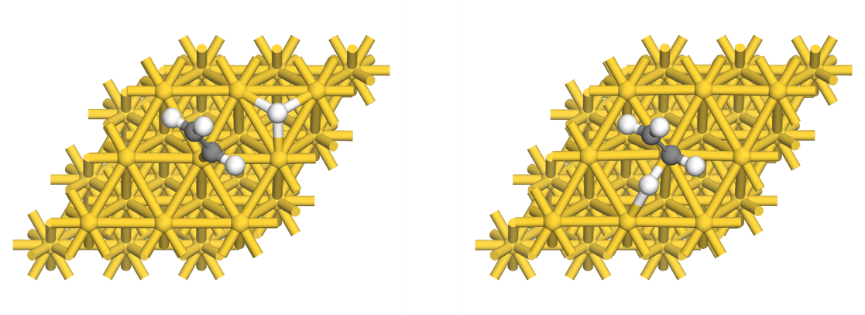


**Figure S34.** The molecular configuration of the initial state and transition state of the hydrogenation on *HCCH_2_ on different carbon on Au in the LH mechanism, we hide the water molecules to make the configuration more straightforward.


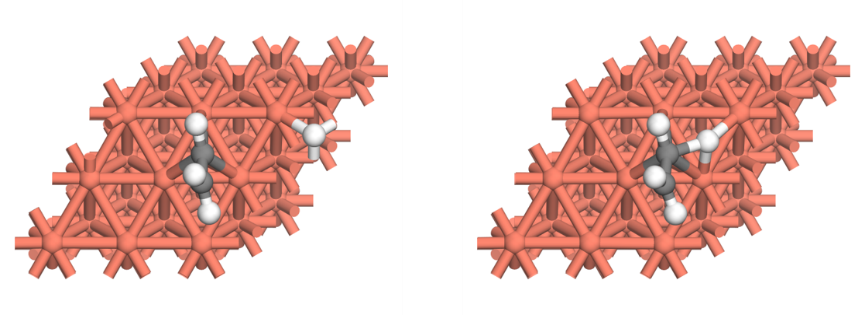


**Figure S35.** The molecular configuration of the initial state and transition state of the hydrogenation on *HCCH_2_ on different carbon on Cu in the LH mechanism, we hide the water molecules to make the configuration more straightforward.


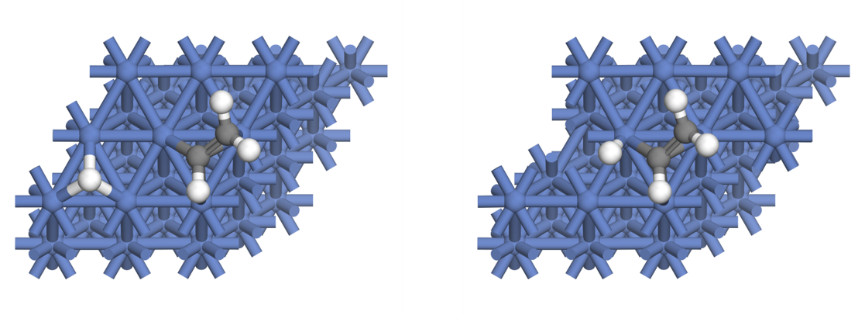


**Figure S36.** The molecular configuration of the initial state and transition state of the hydrogenation on *HCCH_2_ on different carbon on Ni in the LH mechanism, we hide the water molecules to make the configuration more straightforward.


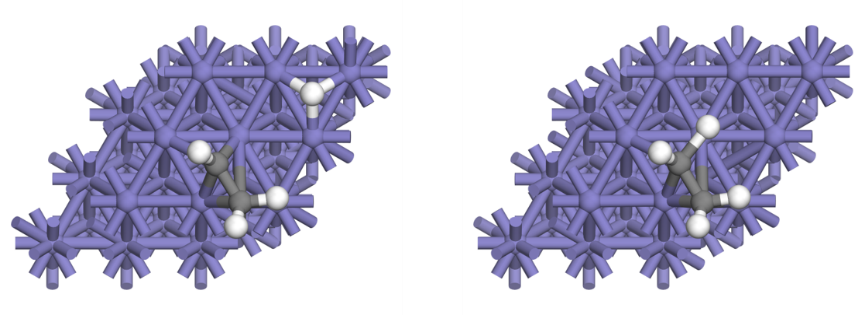


**Figure S37.** The molecular configuration of the initial state and transition state of the hydrogenation on *HCCH_2_ on Fe in the LH mechanism, we hide the water molecules to make the configuration more straightforward.


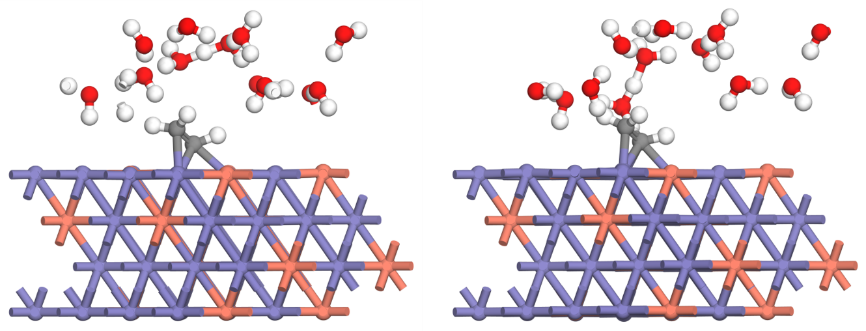


**Figure S38.** The molecular configuration of the initial and transition state of hydrogenation on *HCCH_2_ on CuFe_3_ in ER mechanism.


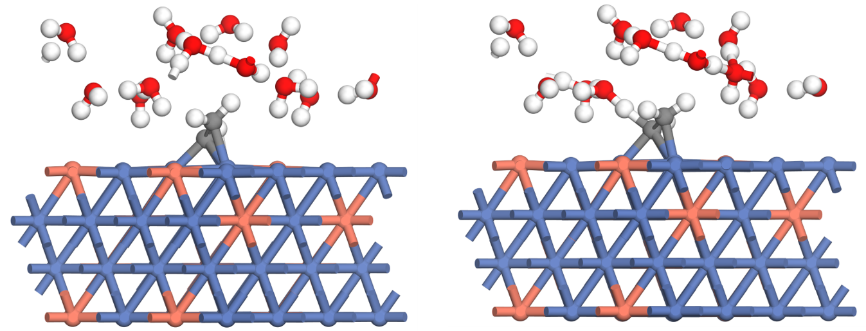


**Figure S39.** The molecular configuration of the initial and transition state of the hydrogenation on *HCCH_2_ on CuNi_3_ in ER mechanism.


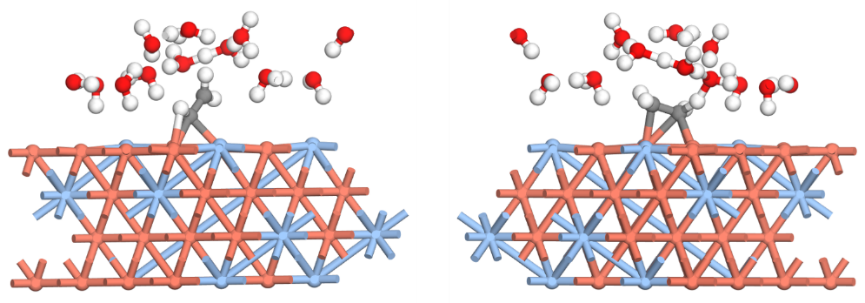


**Figure S40.** The molecular configuration of the initial and transition state of the hydrogenation on *HCCH_2_ on Cu_3_Ag in ER mechanism.


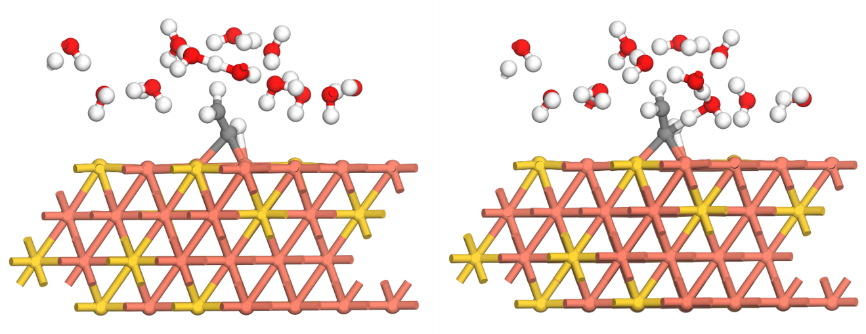


**Figure S41.** The molecular configuration of the initial and transition state of the hydrogenation on *HCCH_2_ on Cu_3_Au in ER mechanism.


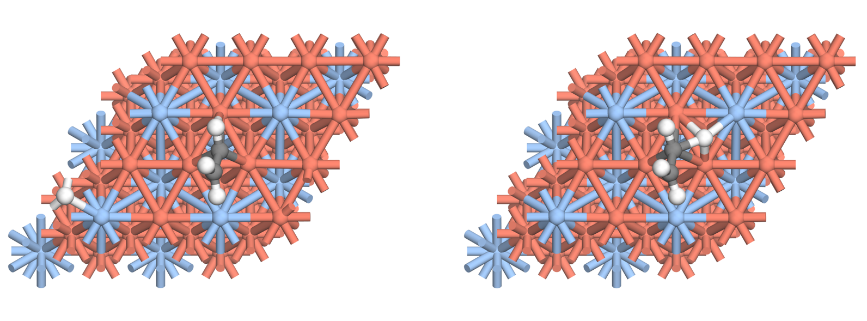


**Figure S42.** The molecular configuration of the initial and transition state of the hydrogenation on *HCCH_2_ on Cu_3_Ag in the LH mechanism, we hide the water molecules to make the configuration more straightforward.


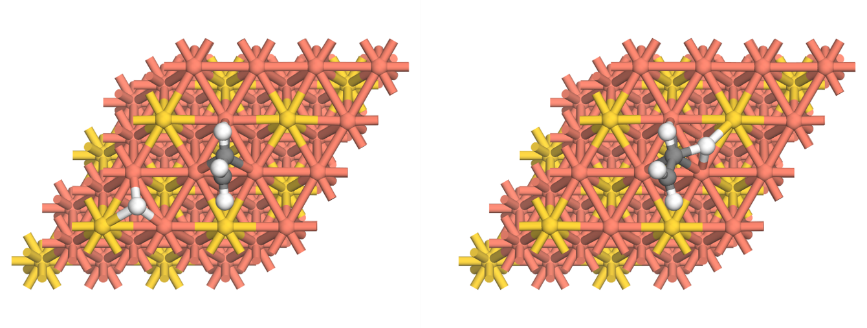


**Figure S43.** The molecular configuration of the initial and transition state of the hydrogenation on *HCCH_2_ on Cu_3_Au in the LH mechanism, we hide the water molecules to make the configuration more straightforward.


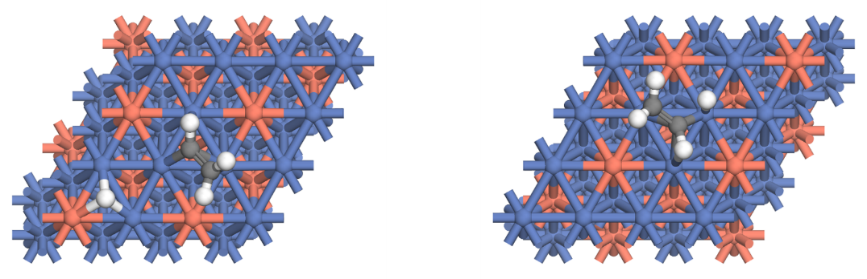


**Figure S44.** The molecular configuration of the initial and transition state of the hydrogenation on *HCCH_2_ on CuNi_3_ in the LH mechanism, we hide the water molecules to make the configuration more straightforward.


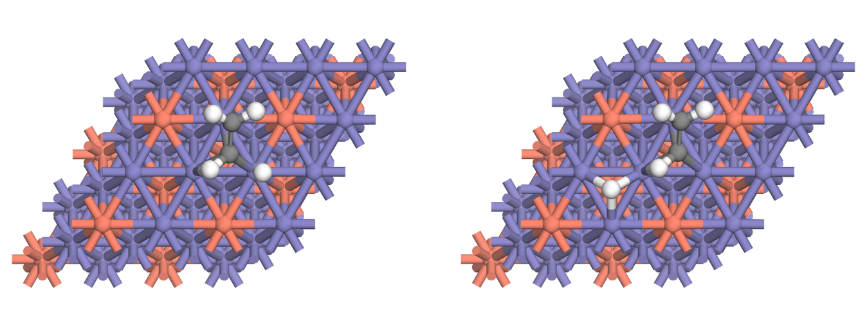


**Figure S45.** The molecular configuration of the initial and transition state of the hydrogenation on *HCCH_2_ on CuFe_3_ in the LH mechanism, we hide the water molecules to make the configuration more straightforward.


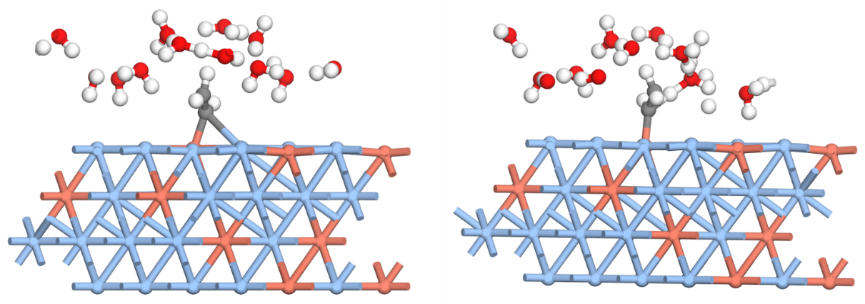


**Figure S46.** The molecular configuration of the initial and transition state of the hydrogenation on *HCCH_2_ on CuAg_3_ in ER mechanism.


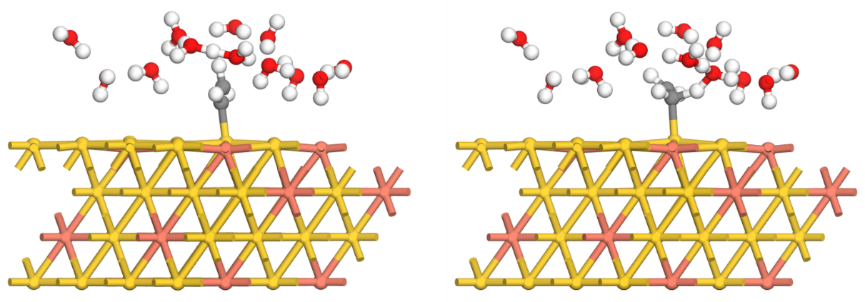


**Figure S47.** The molecular configuration of the initial and transition states of the hydrogenation on *HCCH_2_ on CuAu_3_ in ER mechanism.


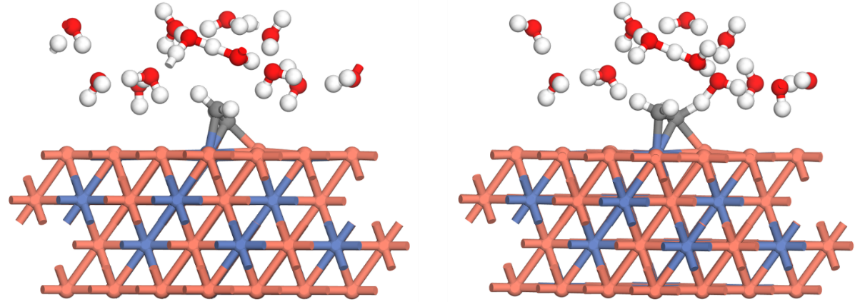


**Figure S48.** The molecular configuration of the initial and transition state of the hydrogenation on *HCCH_2_ on Cu_3_Ni in ER mechanism.


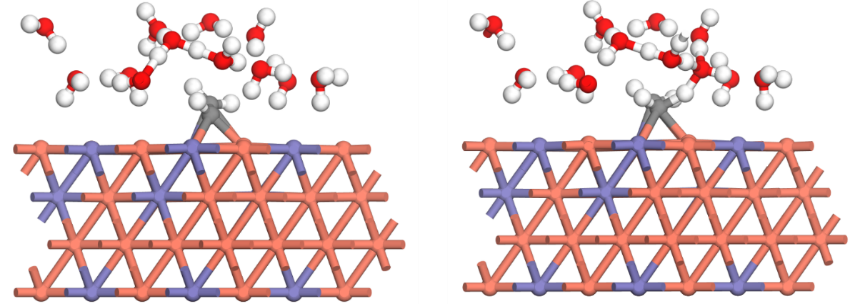


**Figure S49.** The molecular configuration of the initial and transition state of the hydrogenation on *HCCH_2_ on Cu_3_Fe in ER mechanism.


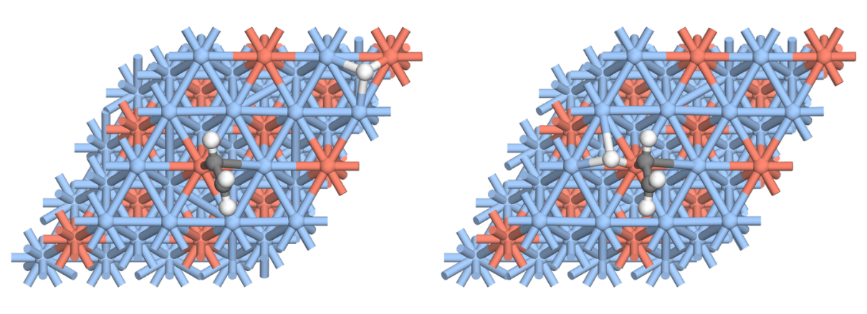


**Figure S50.** The molecular configuration of the initial and transition state of the hydrogenation on *HCCH_2_ on CuAg_3_ in the LH mechanism, we hide the water molecules to make the configuration more straightforward.


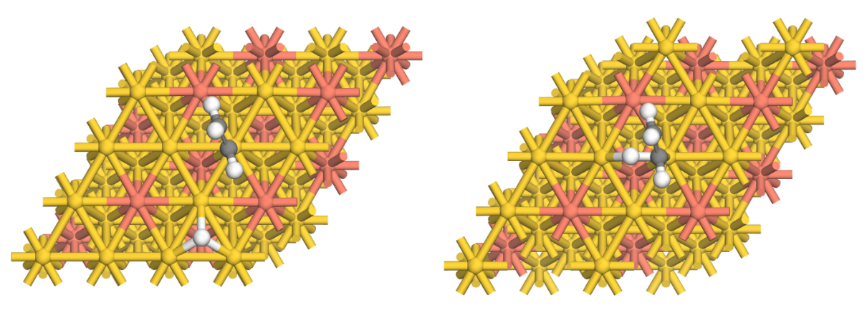


**Figure S51.** The molecular configuration of the initial and transition state of the hydrogenation on *HCCH_2_ on CuAu_3_ in the LH mechanism, we hide the water molecules to make the configuration more straightforward.


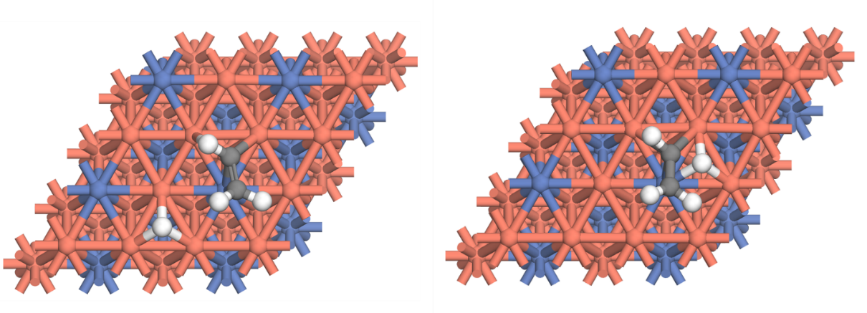


**Figure S52.** The molecular configuration of the initial and transition state of the hydrogenation on *HCCH_2_ on Cu_3_Ni in the LH mechanism, we hide the water molecules to make the configuration more straightforward.


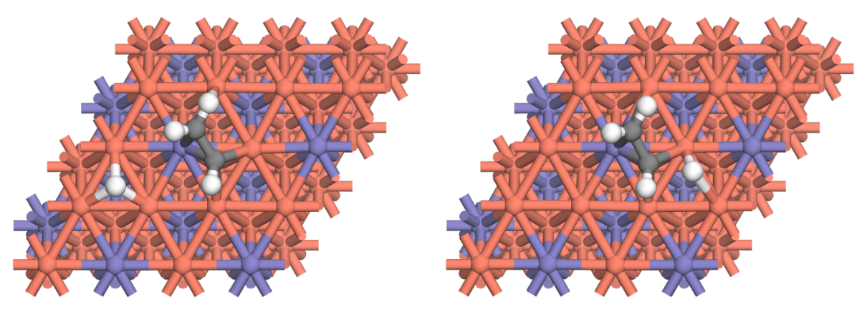


**Figure S53.** The molecular configuration of the initial state and transition state of the hydrogenation on *HCCH_2_ on Cu_3_Fe in the LH mechanism, we hide the water molecules to make the configuration more straightforward.


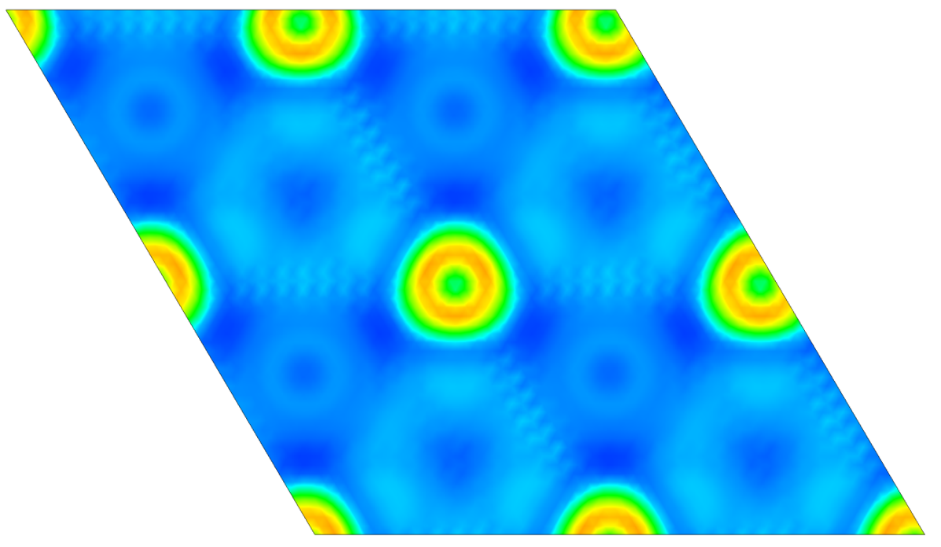


**Figure S54.** The electron localization function of the Cu_2_O.


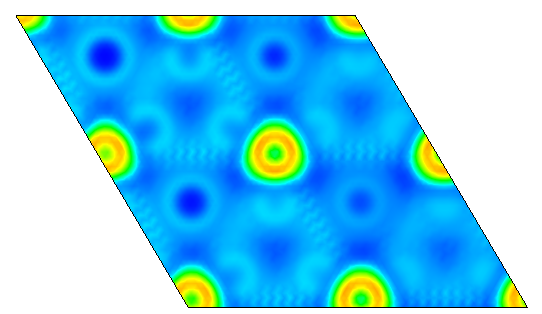


**Figure S55.** The electron localization function of the O_vac_-Cu_2_O.


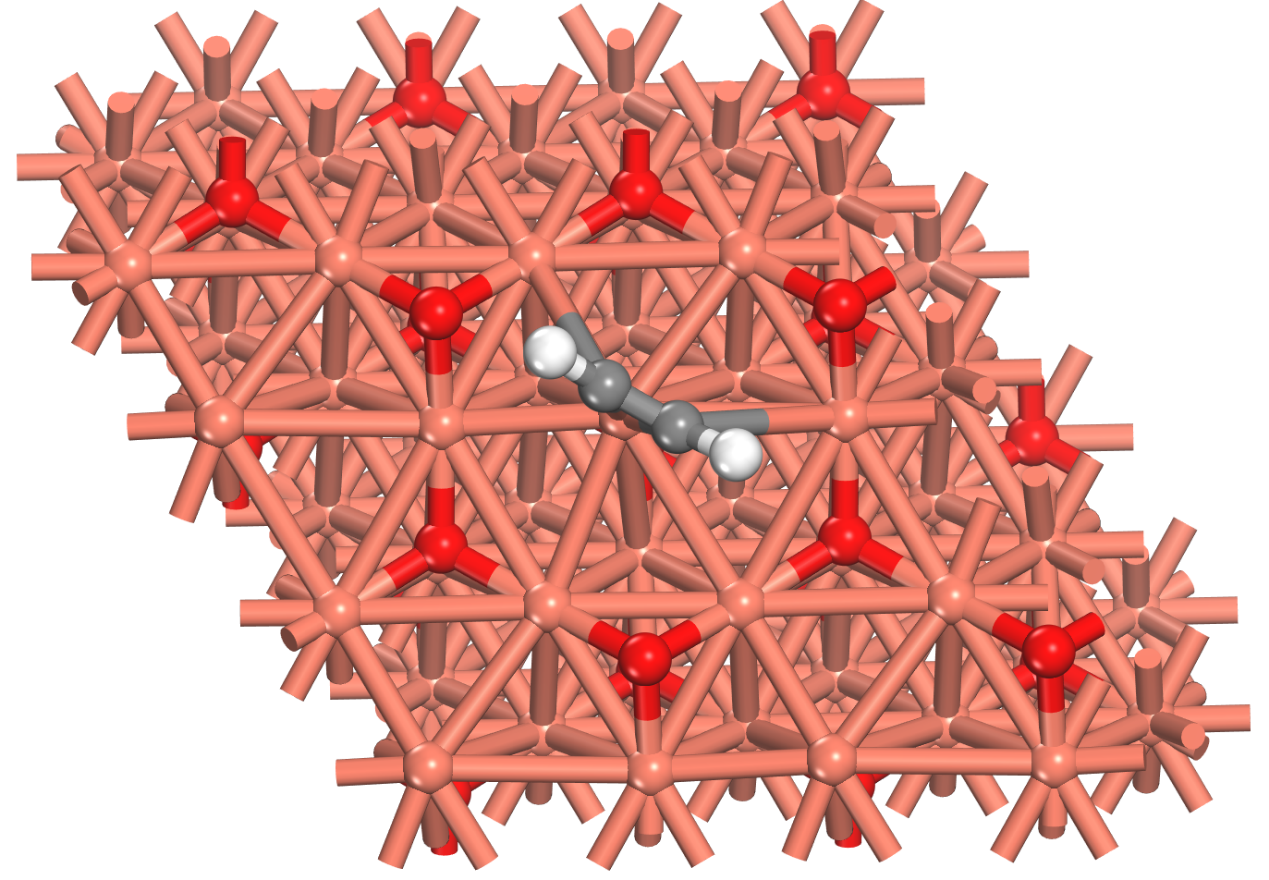
**Figure S56.** The stable configuration when C_2_H_2_ is adsorbed on the Cu_2_O-site1


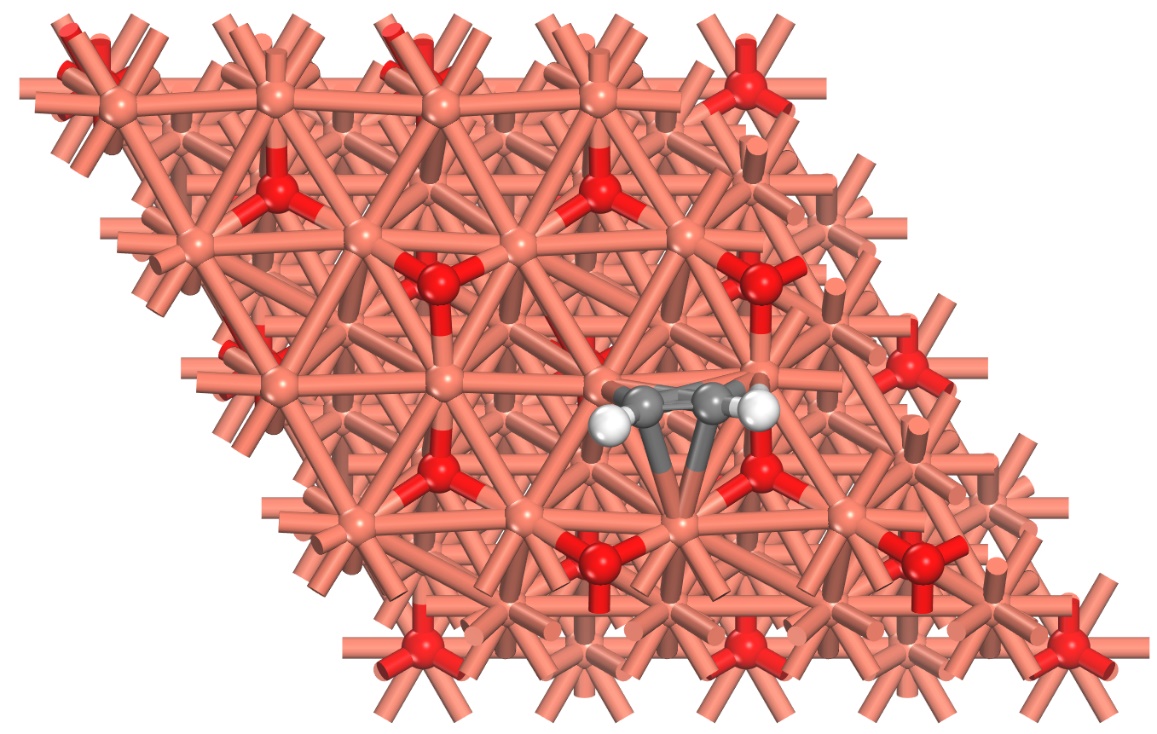


**Figure S57.** The stable configuration when C_2_H_2_ is absorbed on the Cu_2_O-site2.


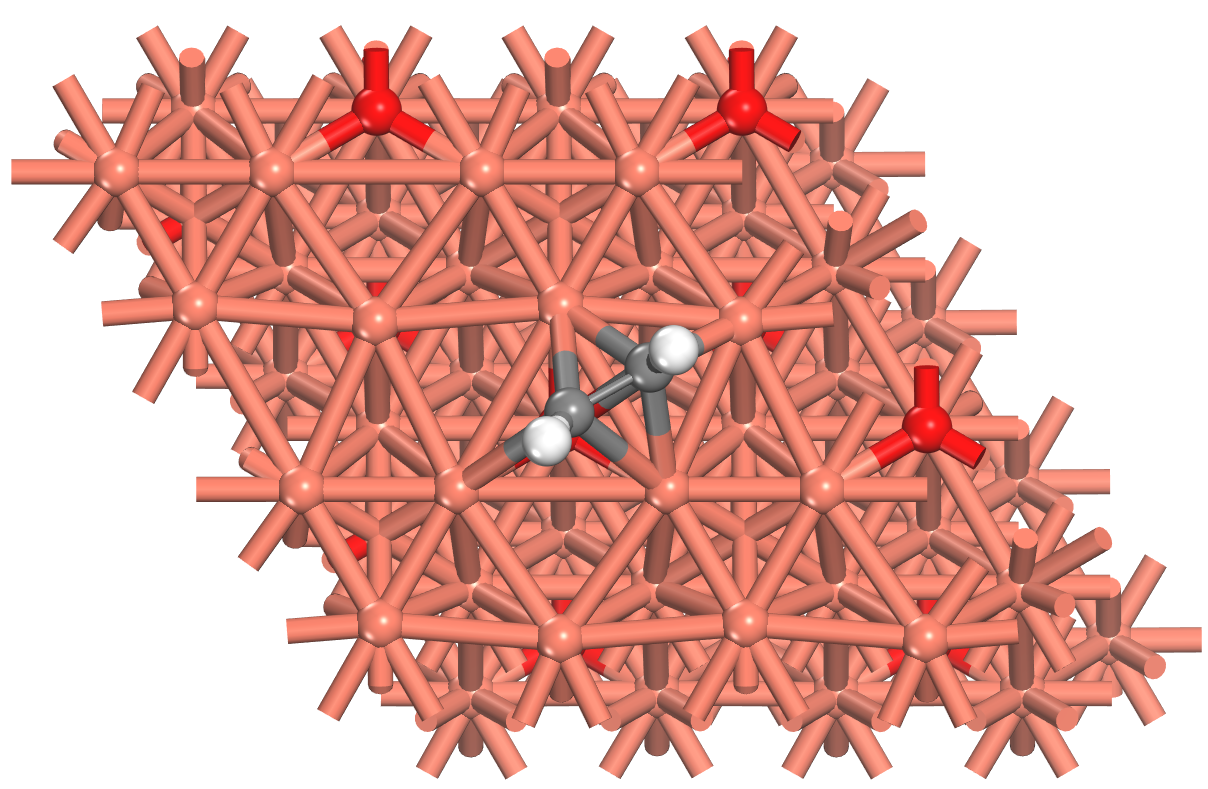


**Figure S58.** The stable configuration when C_2_H_2_ is adsorbed on the O_vac_-Cu_2_O slab


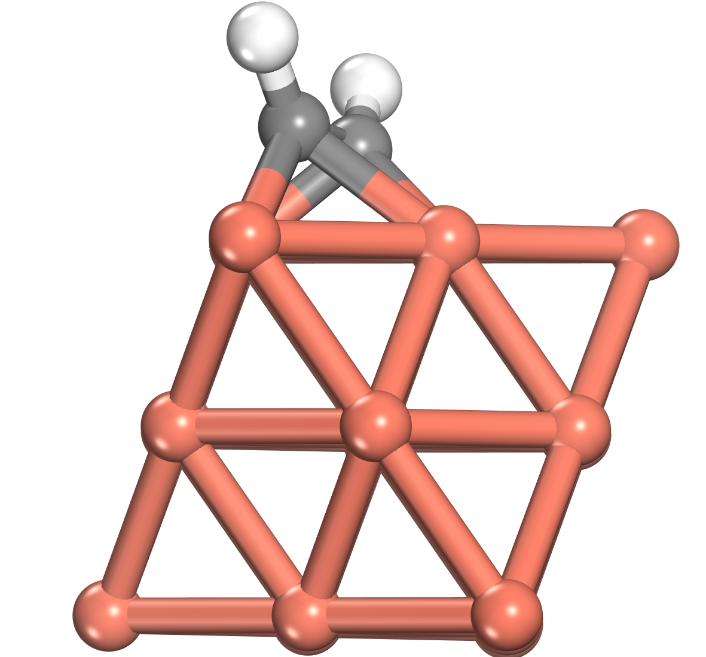


**Figure S59.** C_2_H_2_ absorbed the Cu nanoparticle of Cu_19._


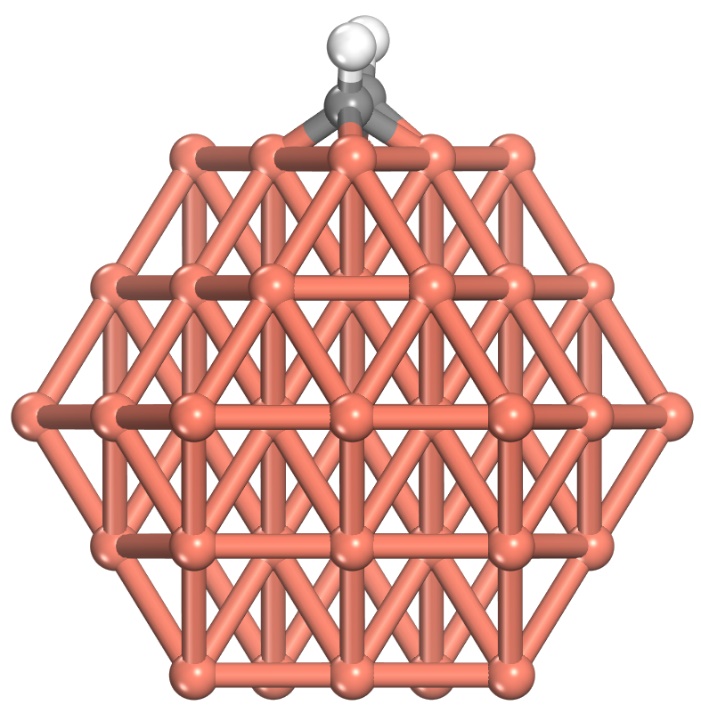


**Figure S60.** C_2_H_2_ absorbed the Cu nanoparticle of Cu_55._


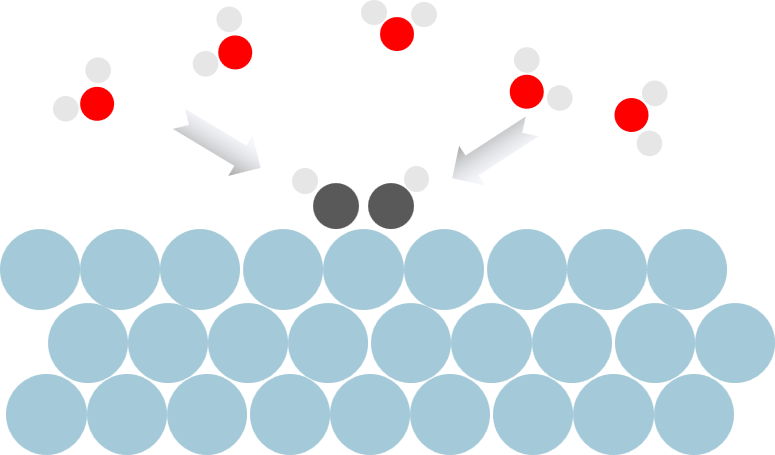


**Figure S61.** The sketch when C_2_H_2_ was absorbed on the slab.


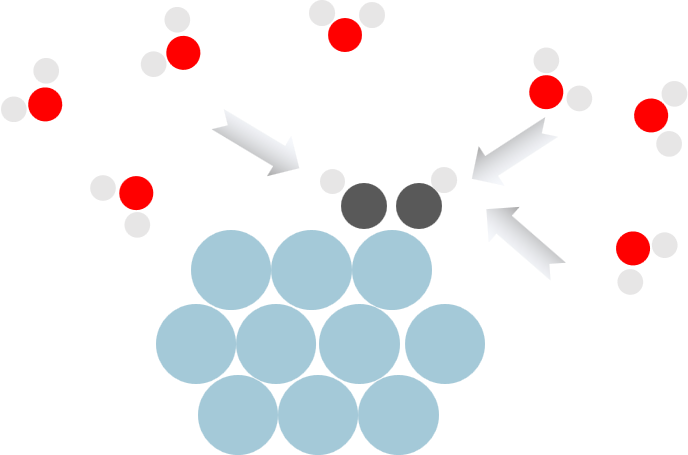


**Figure S62.**The sketch when C_2_H_2_ was absorbed on the nanoparticles.


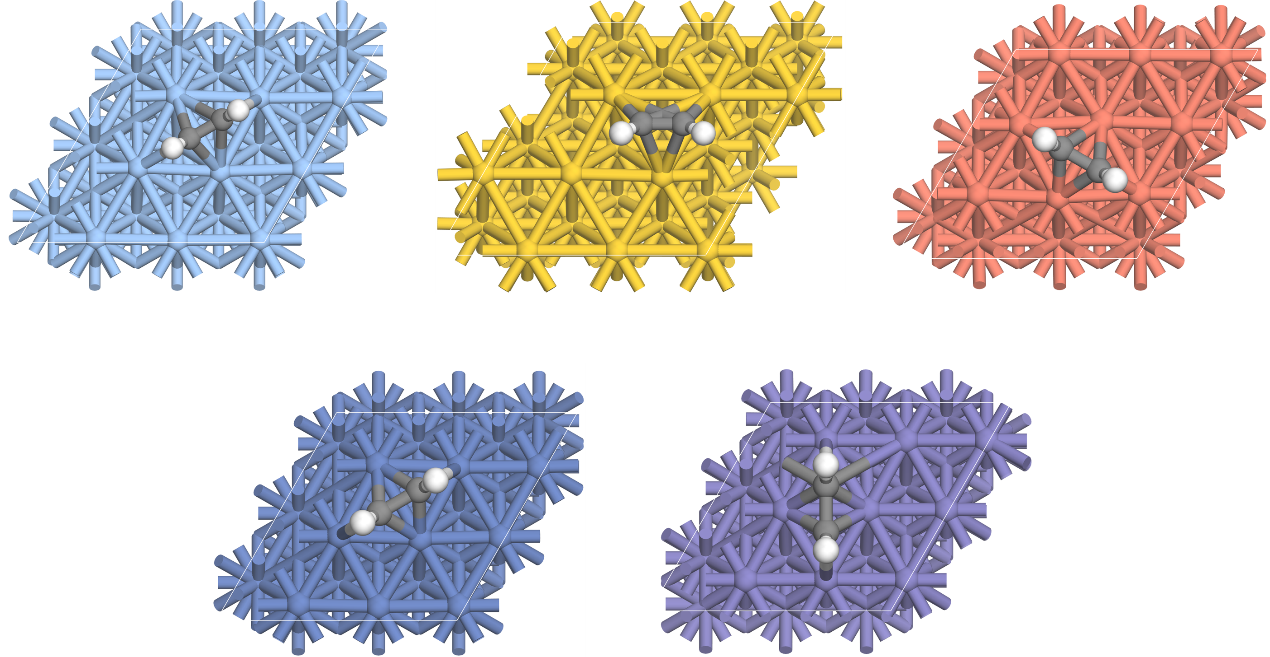


**Figure S63.** The stable configuration when C_2_H_2_ was absorbed on the pure metal (Ag, Au, Cu, Ni, Fe) slab.


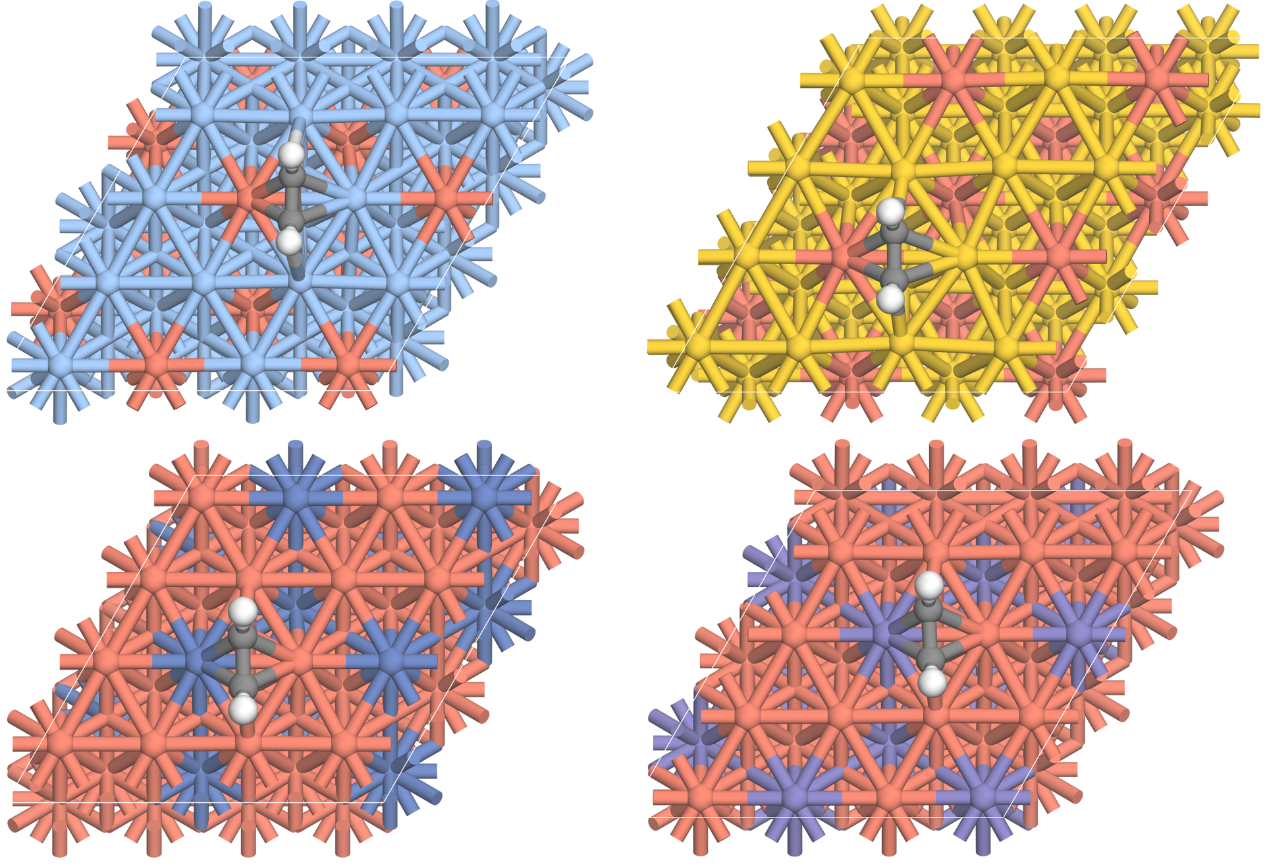


**Figure S64.** The stable configuration when C_2_H_2_ was absorbed on the AB_3_ (CuAg_3_, CuAu_3_, Cu_3_Ni, and Cu_3_Fe) slab.


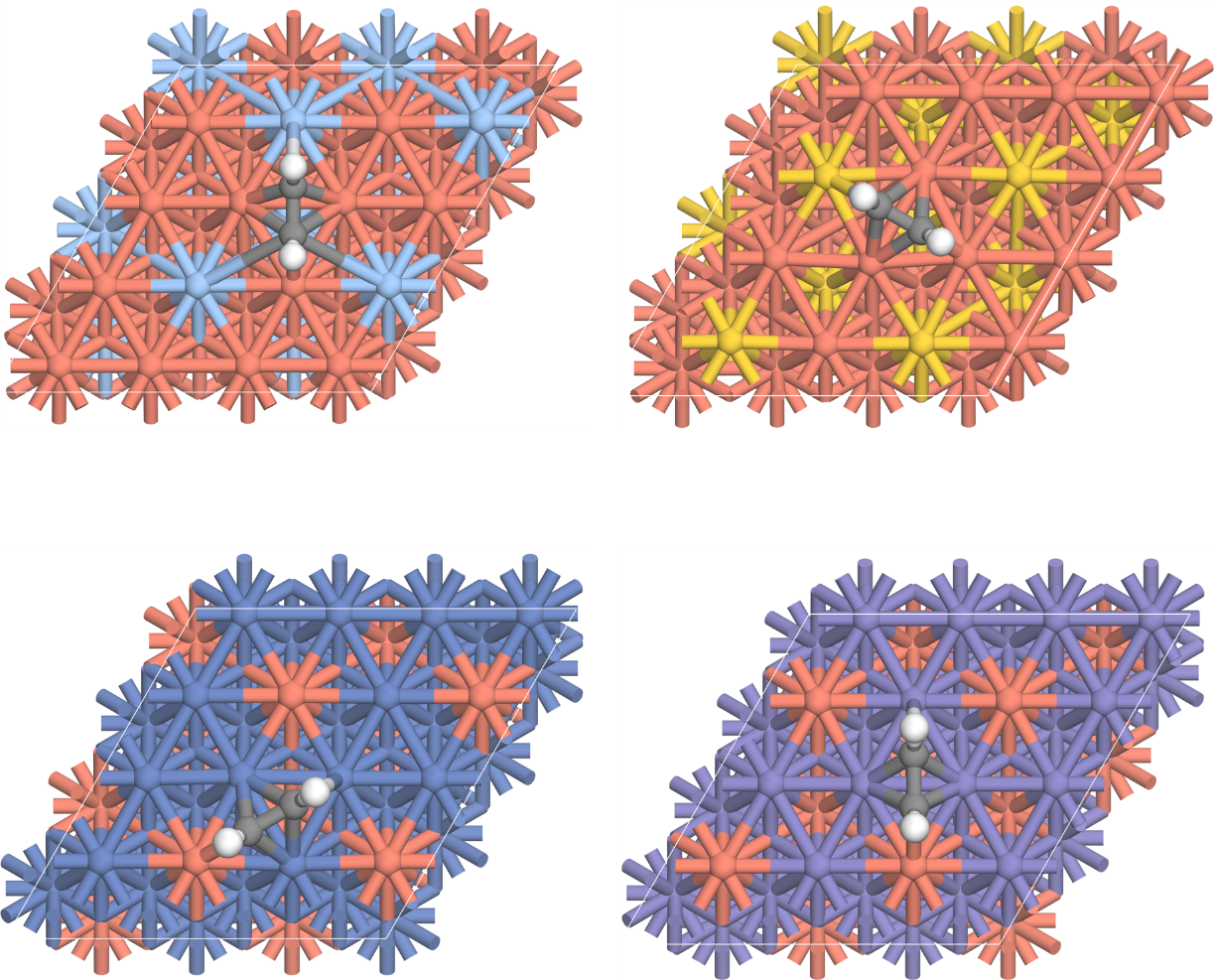
 **Figure S65.** The stable configuration when C_2_H_3_ was absorbed on the A_3_B (Cu_3_Ag, Cu_3_Au, CuNi_3_, and CuFe_3_) slab.


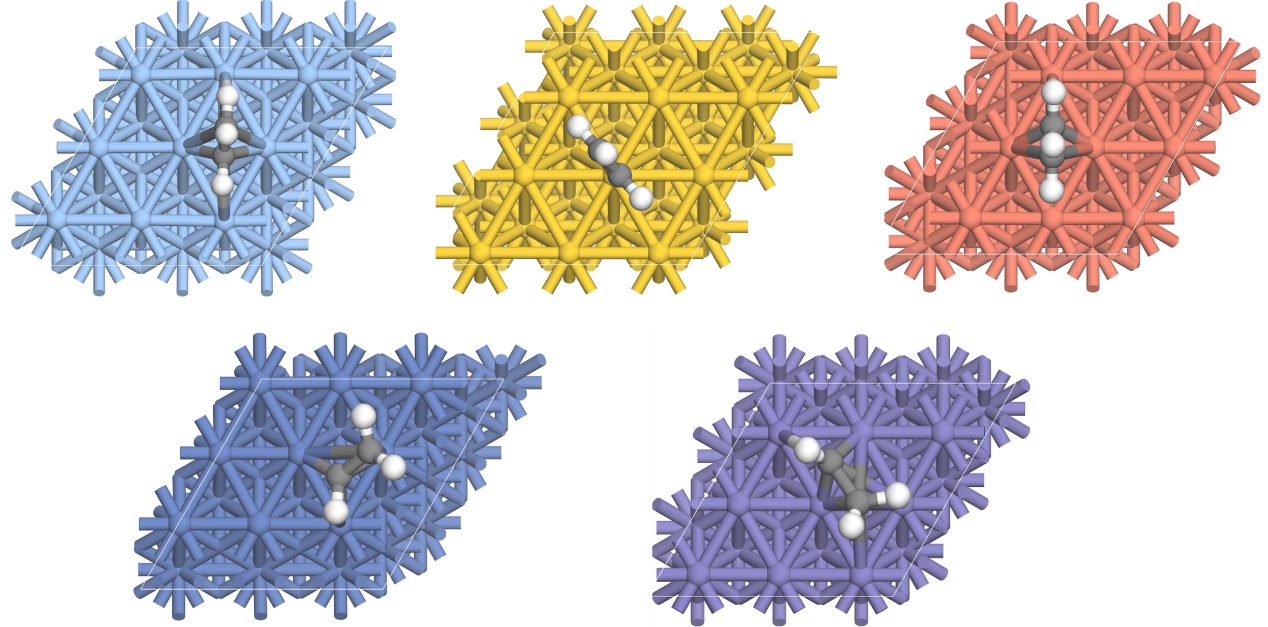


**Figure S66.** The stable configuration when C_2_H_3_ was absorbed on the pure metal (Ag, Au, Cu, Ni, Fe) slab.


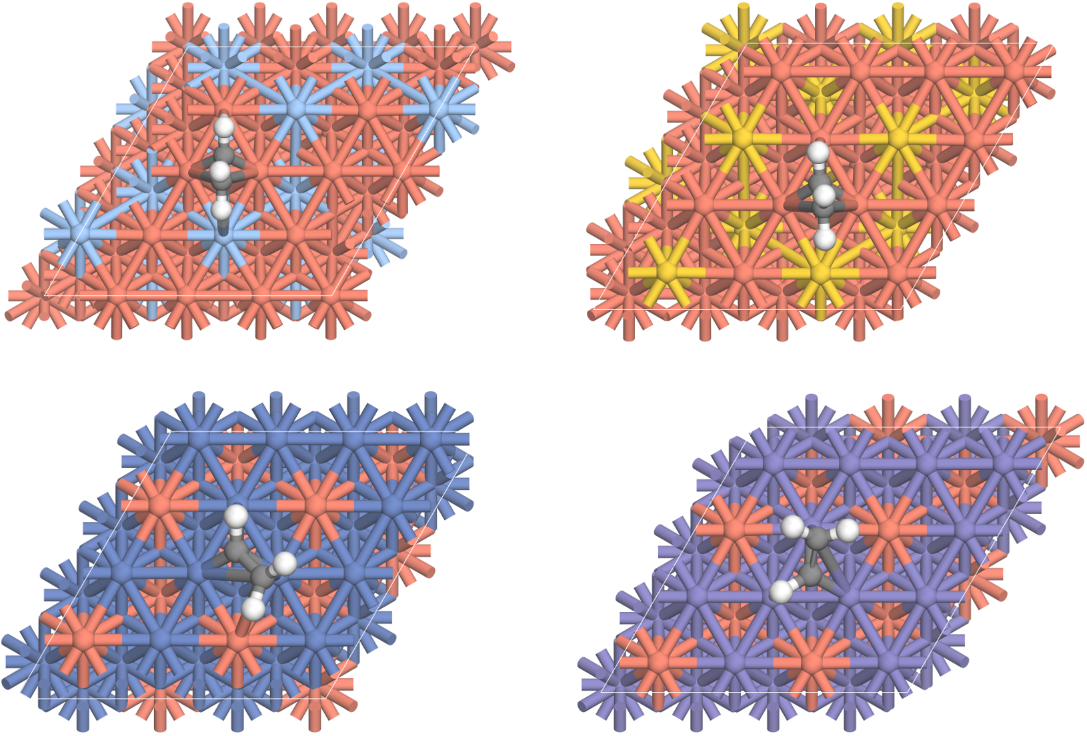


**Figure S67.** The stable configuration when C_2_H_3_ was absorbed on the AB_3_(CuAg_3_, CuAu_3_, Cu_3_Ni, and Cu_3_Fe) slab.


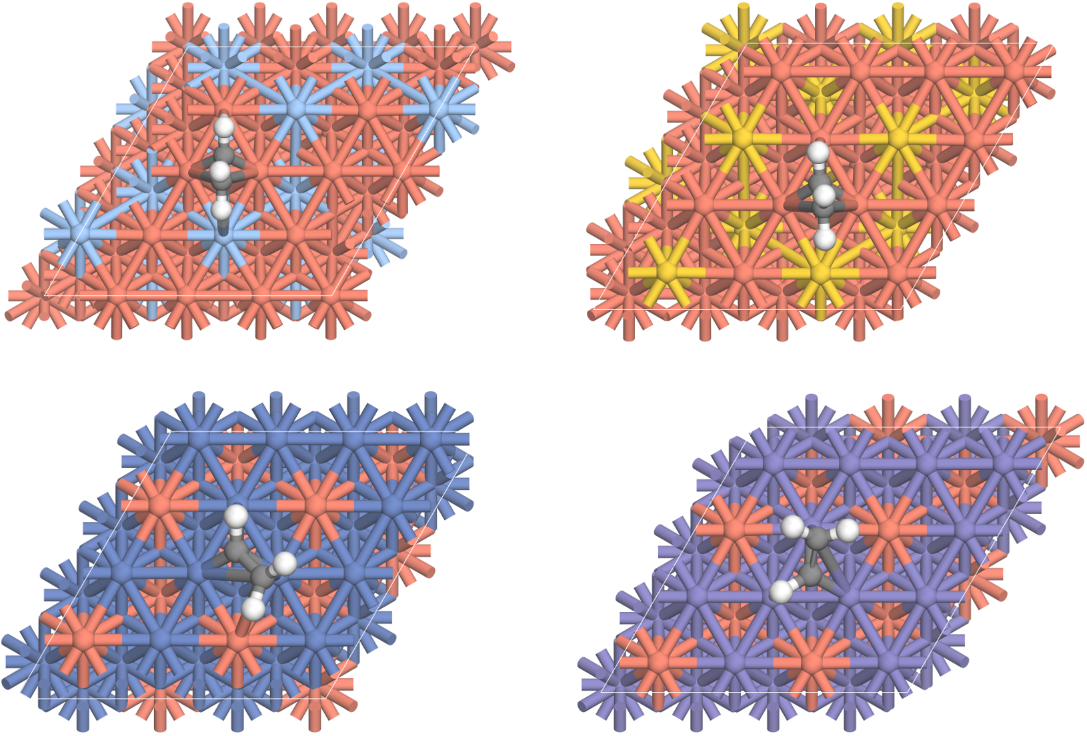


**Figure S68.** The stable configuration when C_2_H_3_ was absorbed on the A_3_B (Cu_3_Ag, Cu_3_Au, CuNi_3_, and CuFe_3_) slab.


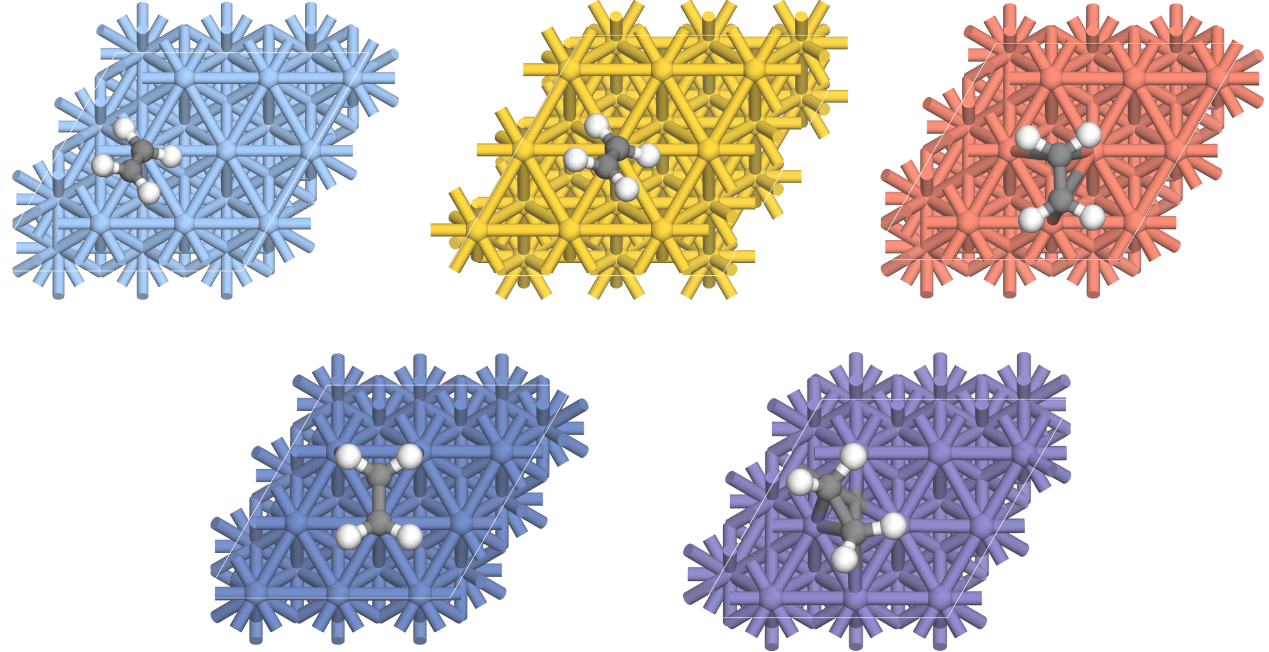


**Figure S69.** The stable configuration when C_2_H_4_ was absorbed on the pure metal (Ag, Au, Cu, Ni, Fe) slab.


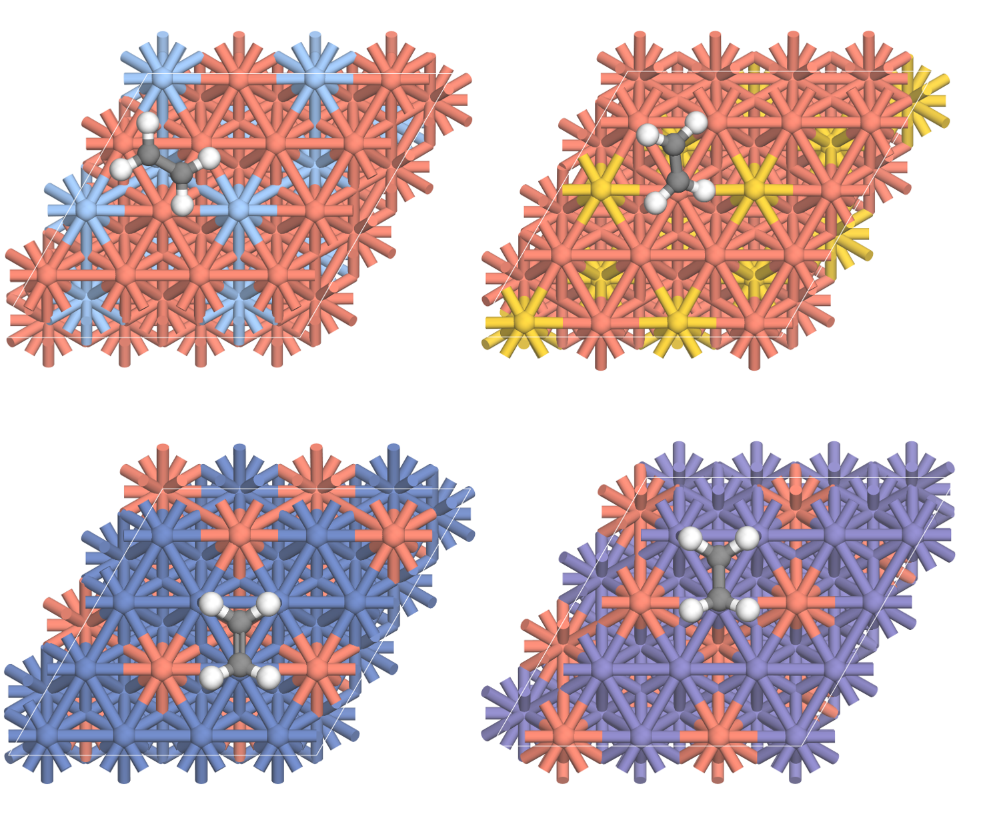


**Figure S70.** The stable configuration when C_2_H_4_ was absorbed on the A_3_B (Cu_3_Ag, Cu_3_Au, CuNi_3_, and CuFe_3_) slab.


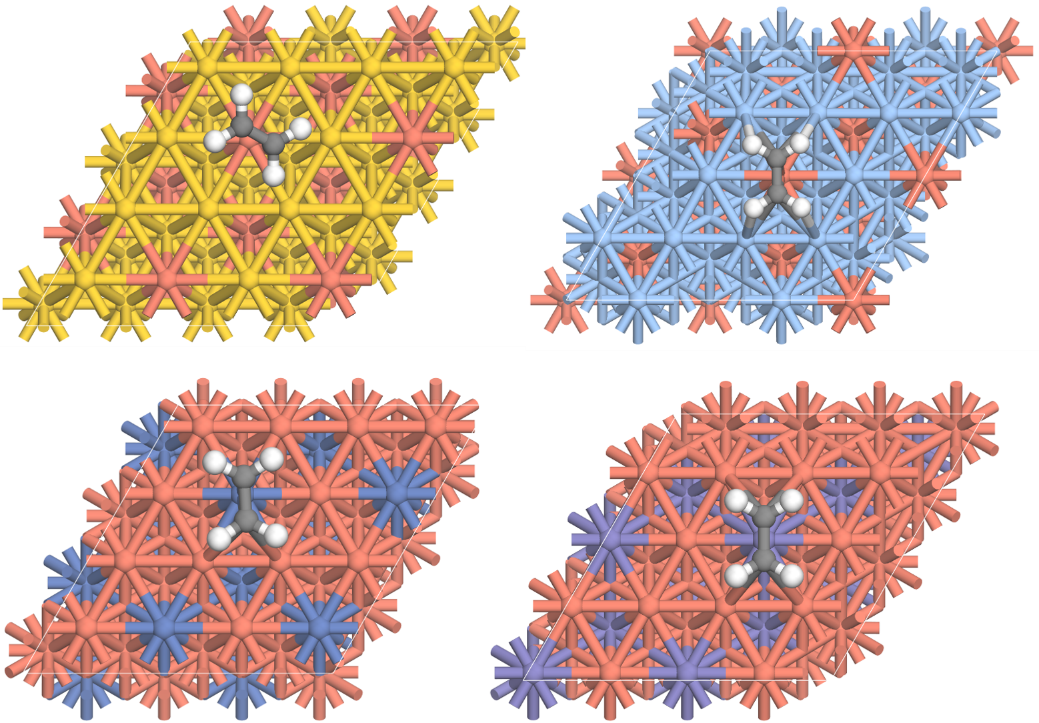


**Figure S71.** The stable configuration when C_2_H_4_ was absorbed on the AB_3_(CuAu_3_, CuAg_3_, Cu_3_Ni, and Cu_3_Fe) slab.


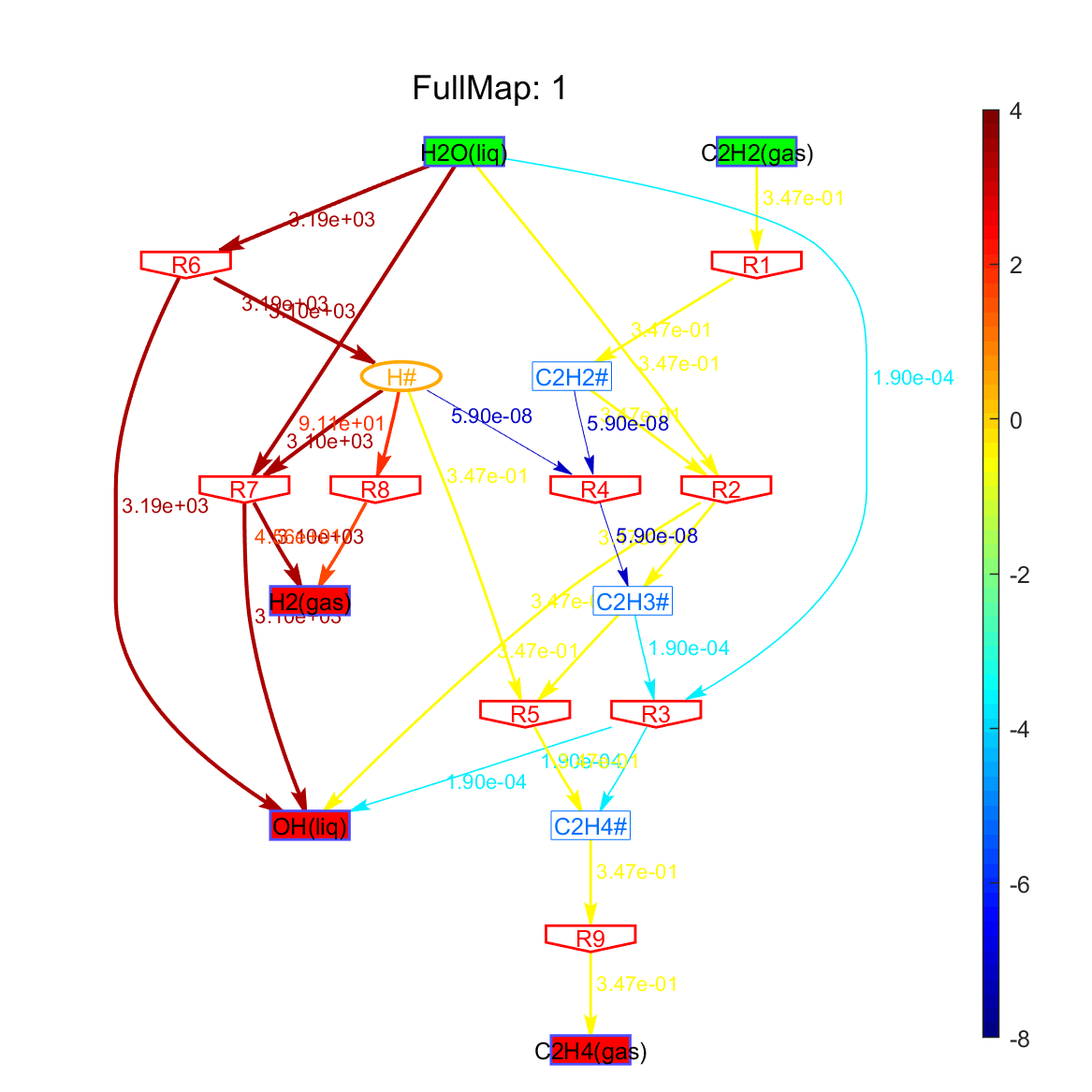


**Figure S72.** The full flow chart of hydrogenation of acetylene on Ag.


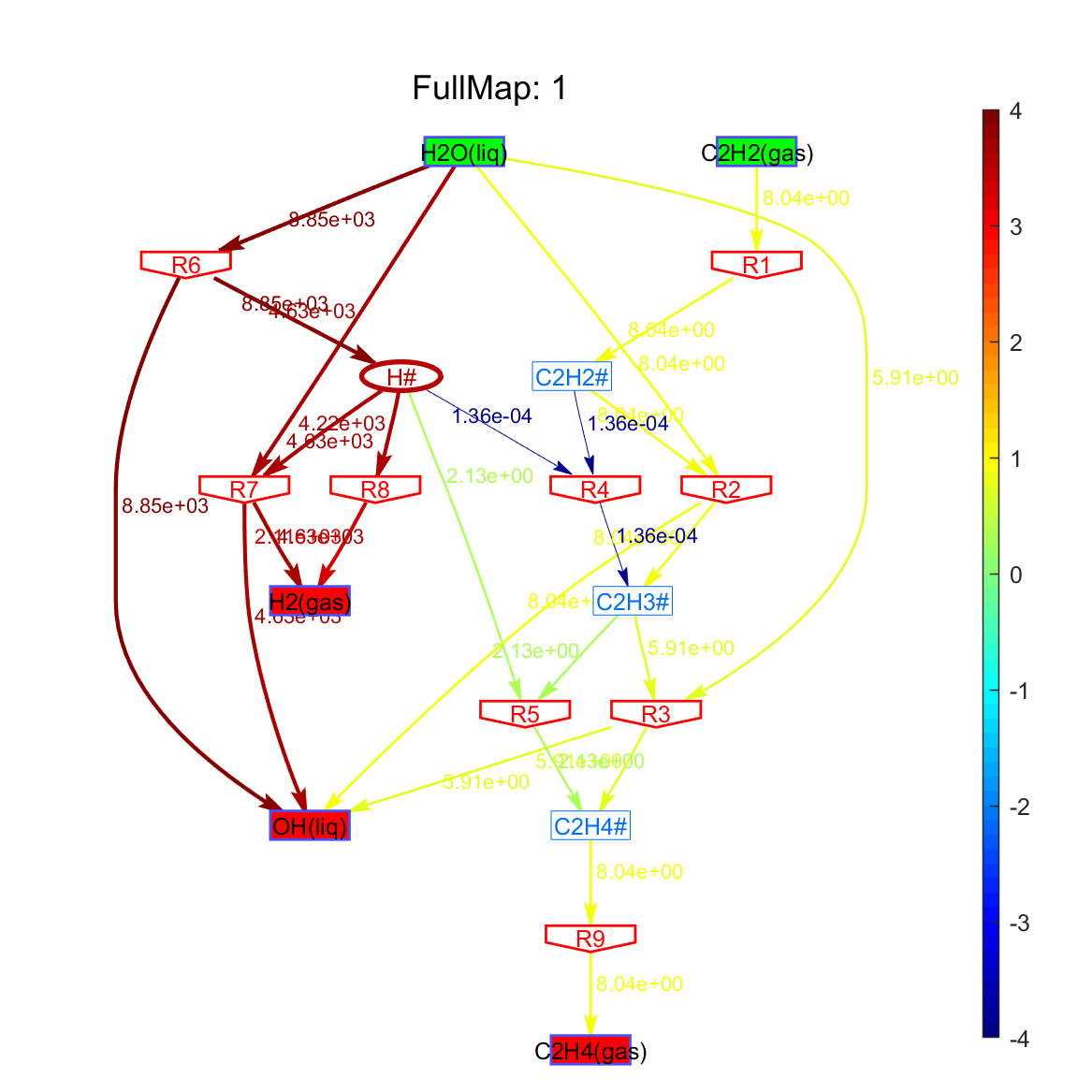


**Figure S73.** The full flow chart of hydrogenation of acetylene on Au.


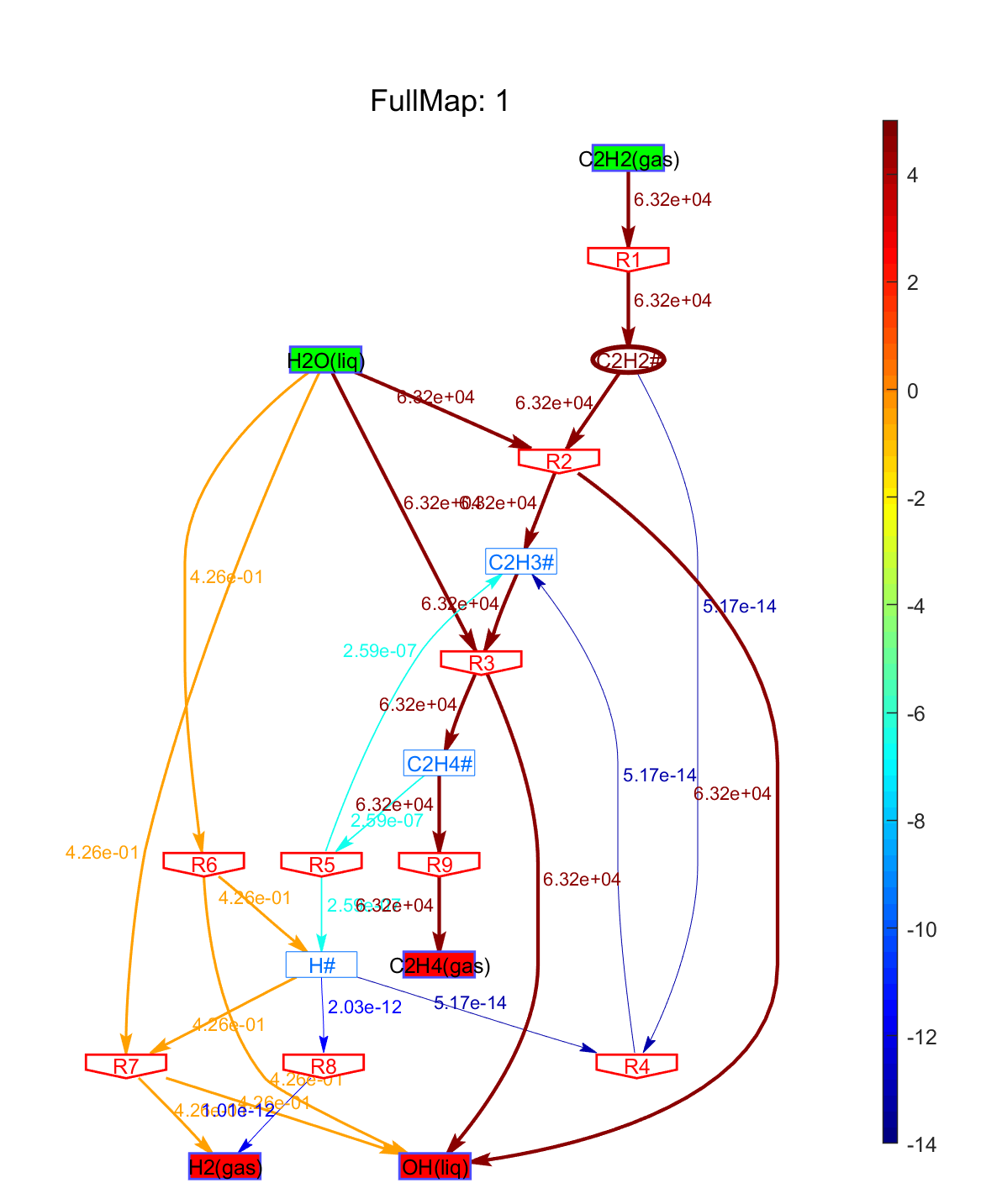


**Figure S74.**  The full flow chart of hydrogenation of acetylene on Cu.


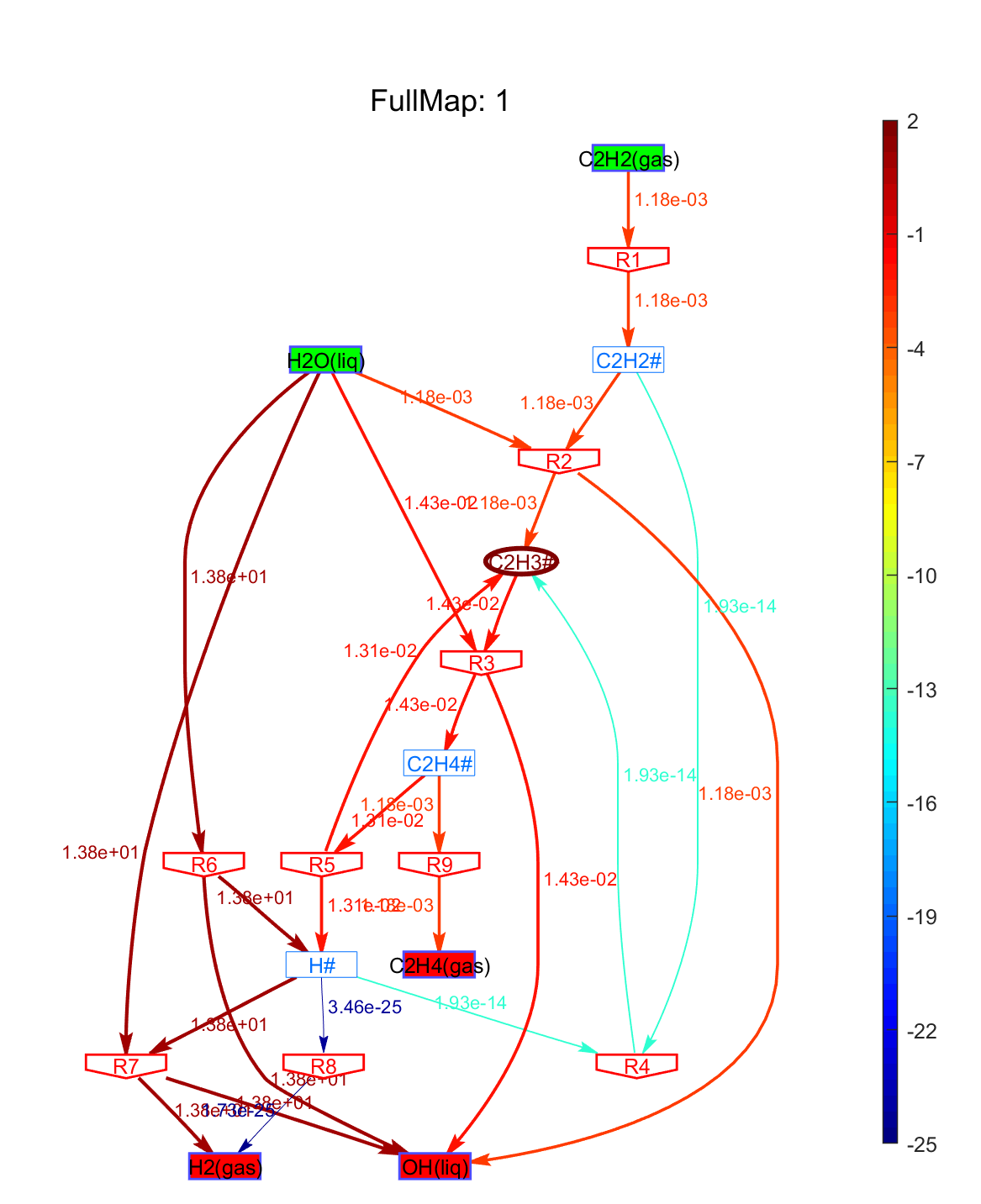


**Figure S75.** The full flow chart of hydrogenation of acetylene on Ni.


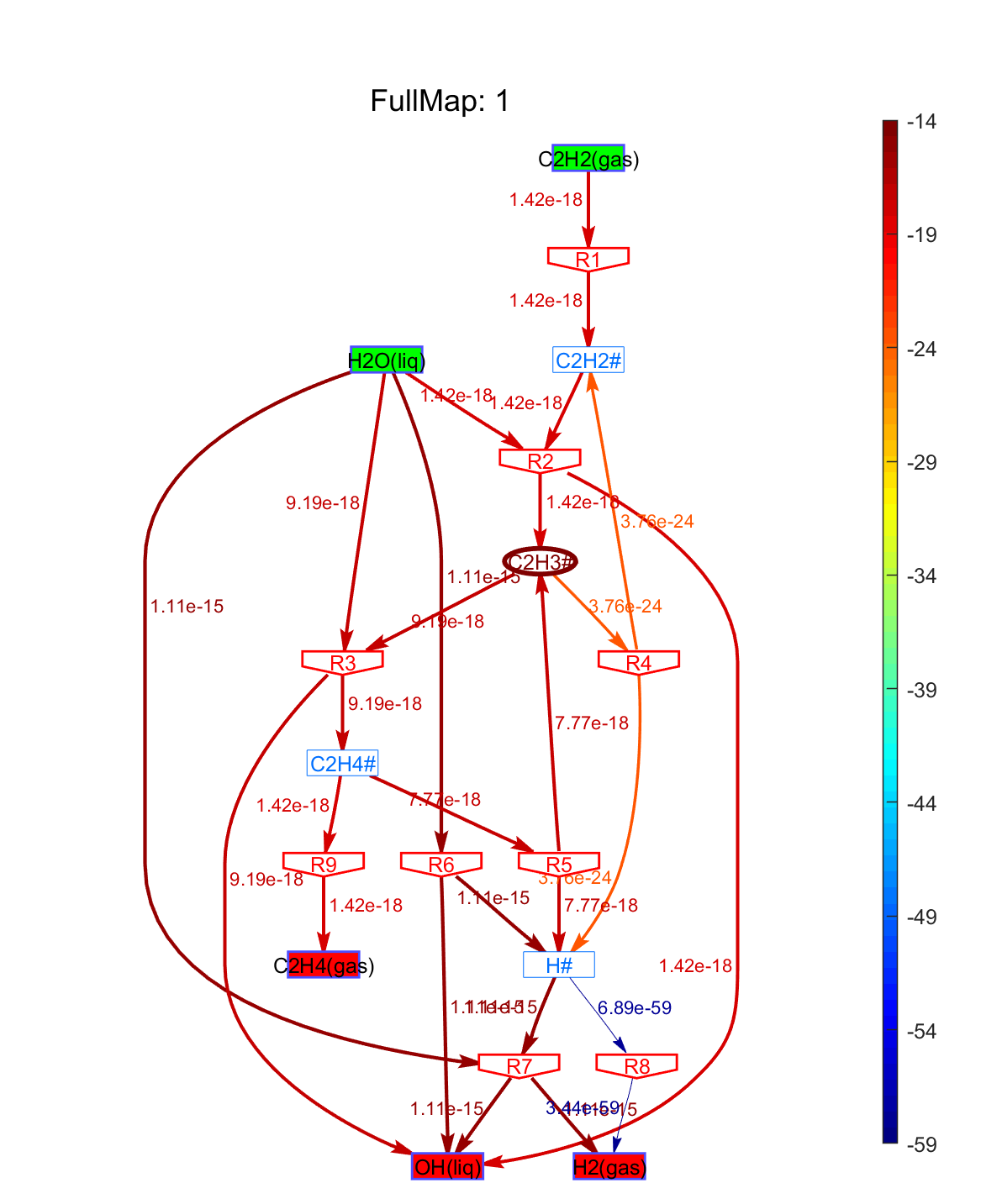


**Figure S76.** The full flow chart of hydrogenation of acetylene on Fe.


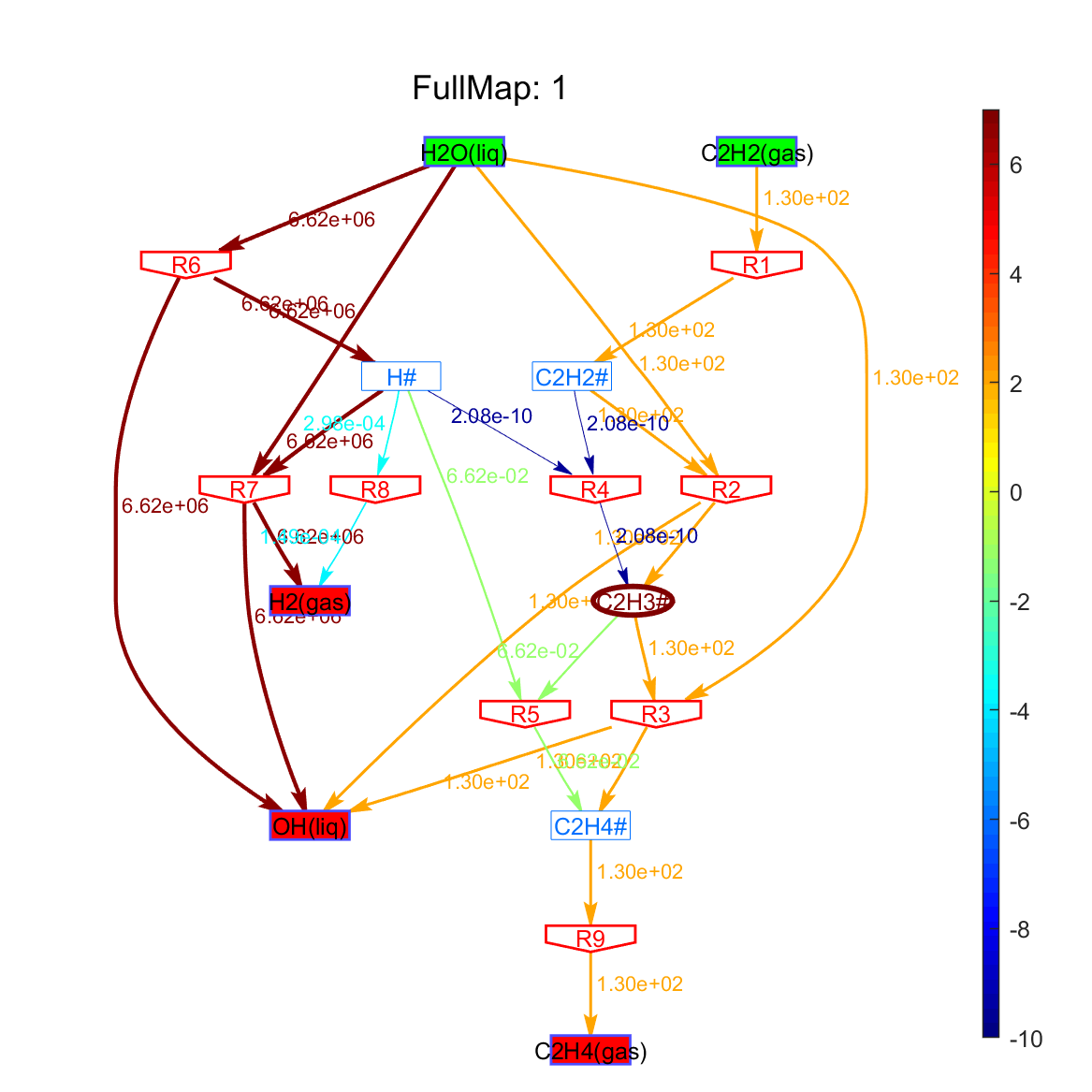
**Figure S77.** The full flow chart of hydrogenation of acetylene on CuAg_3_.


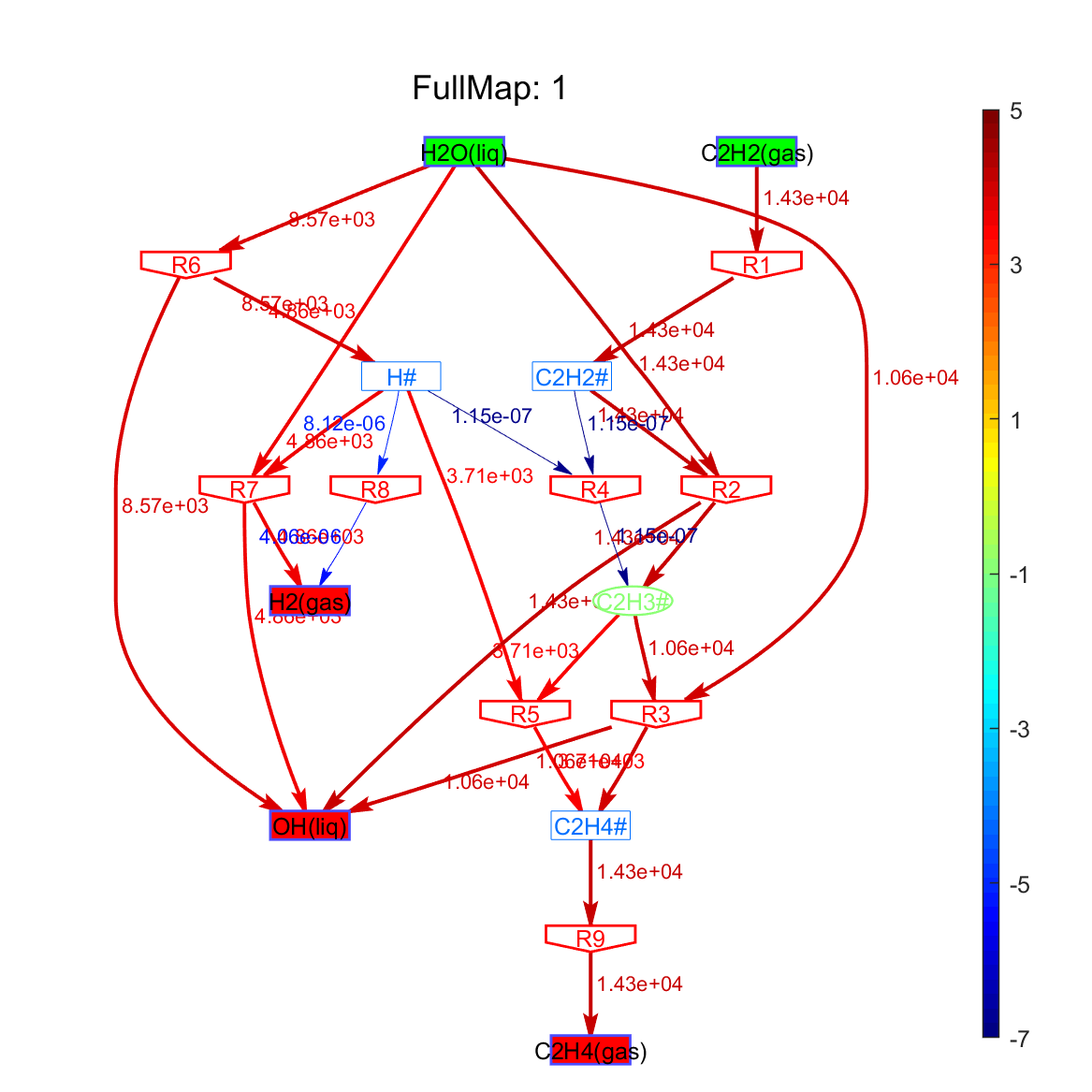


**Figure S78.** The full flow chart of hydrogenation of acetylene on CuAu_3_.


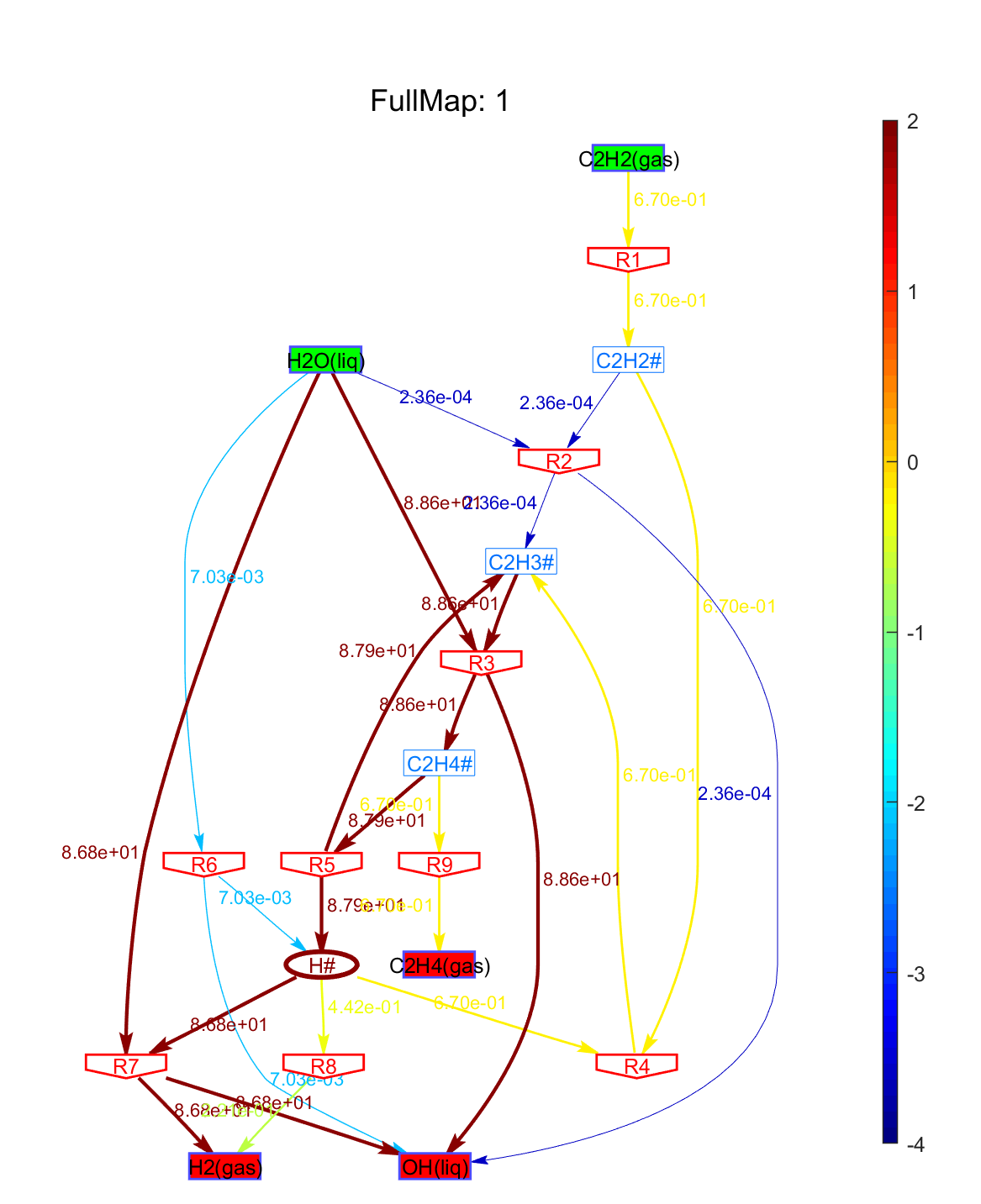


**Figure S79.** The full flow chart of the hydrogenation of acetylene on Cu_3_Ni.


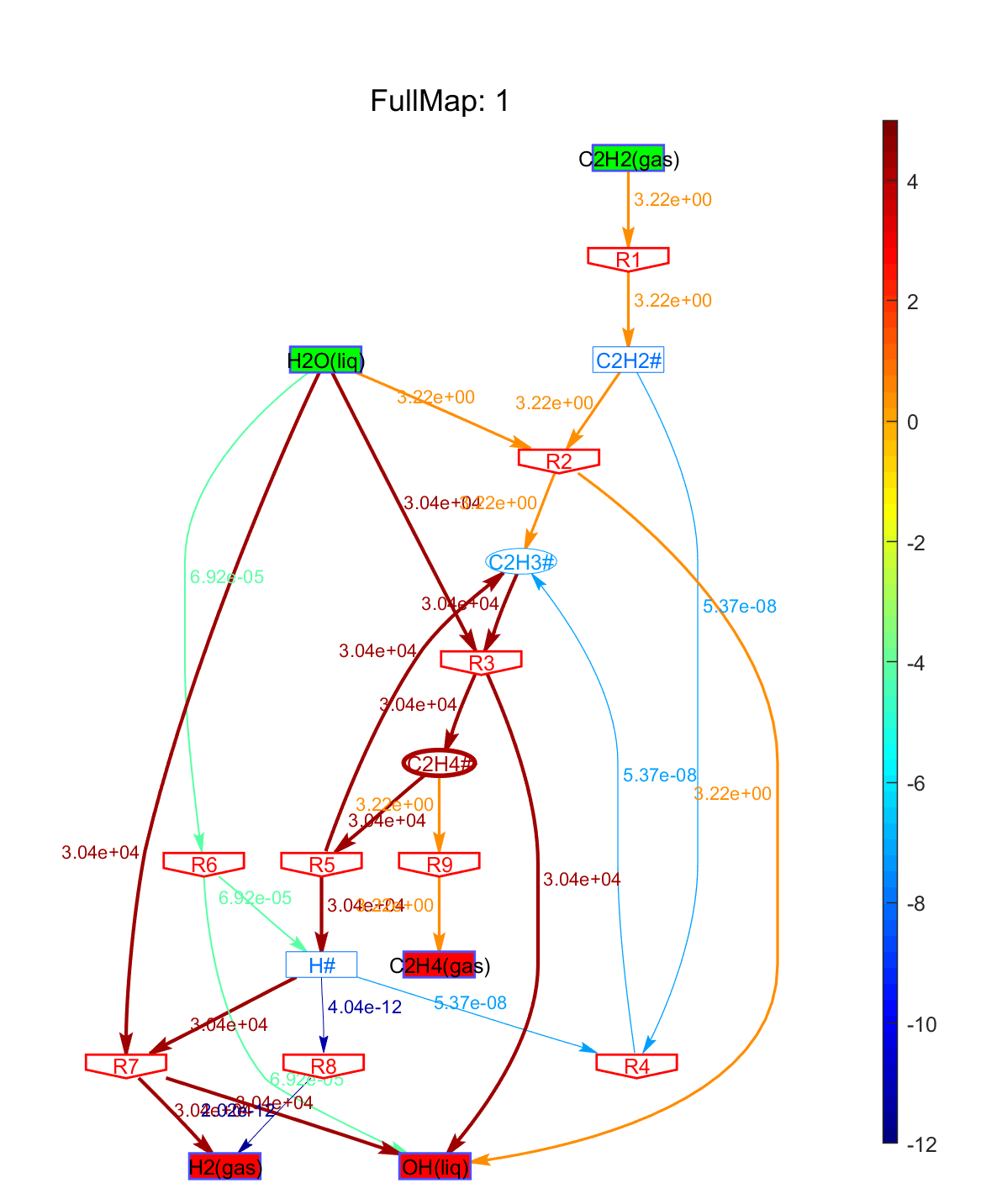


**Figure S80.** The full flow chart of the hydrogenation of acetylene on Cu_3_Fe.


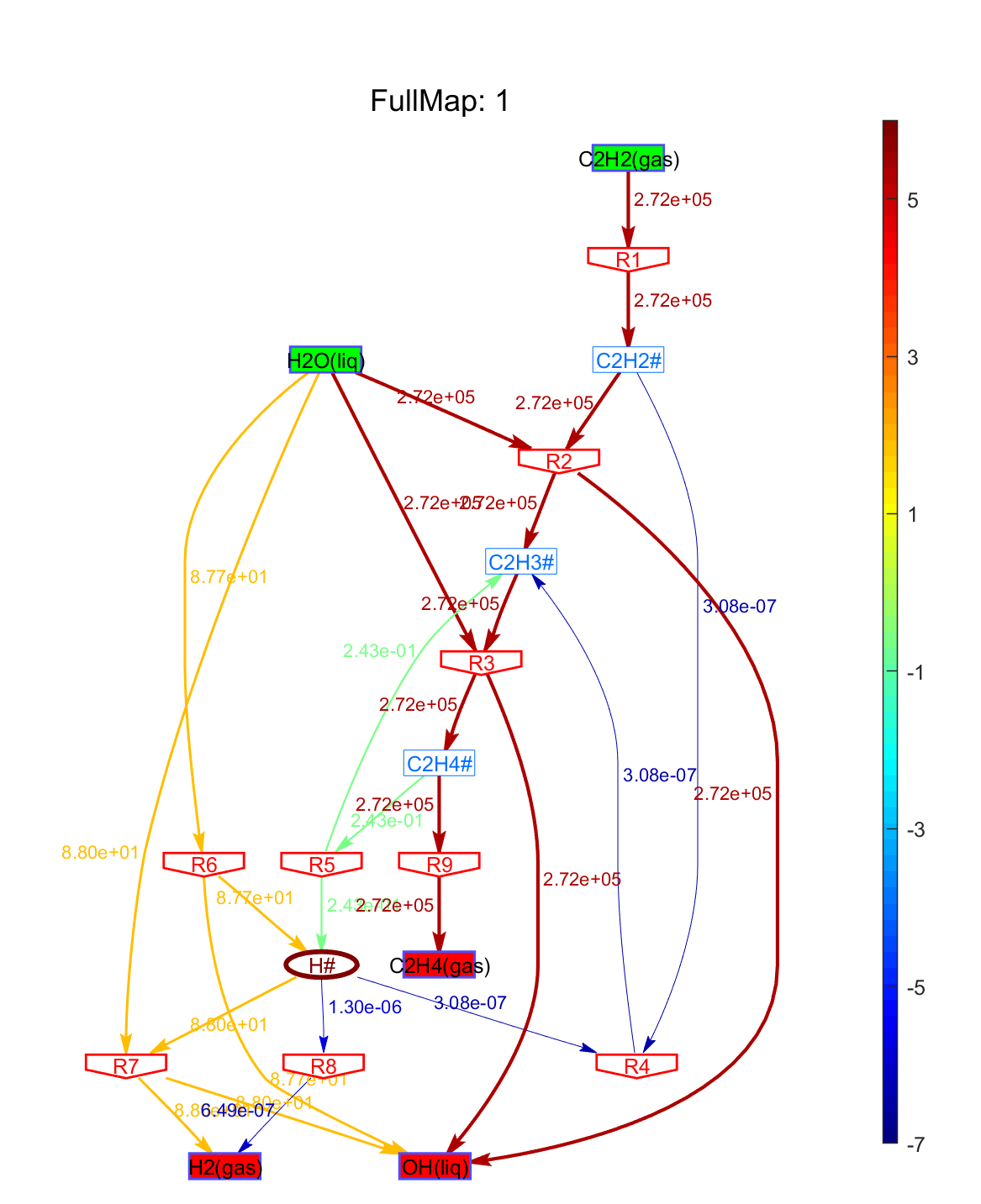


**Figure S81.** The full flow chart of the hydrogenation of acetylene on Cu_3_Ag.


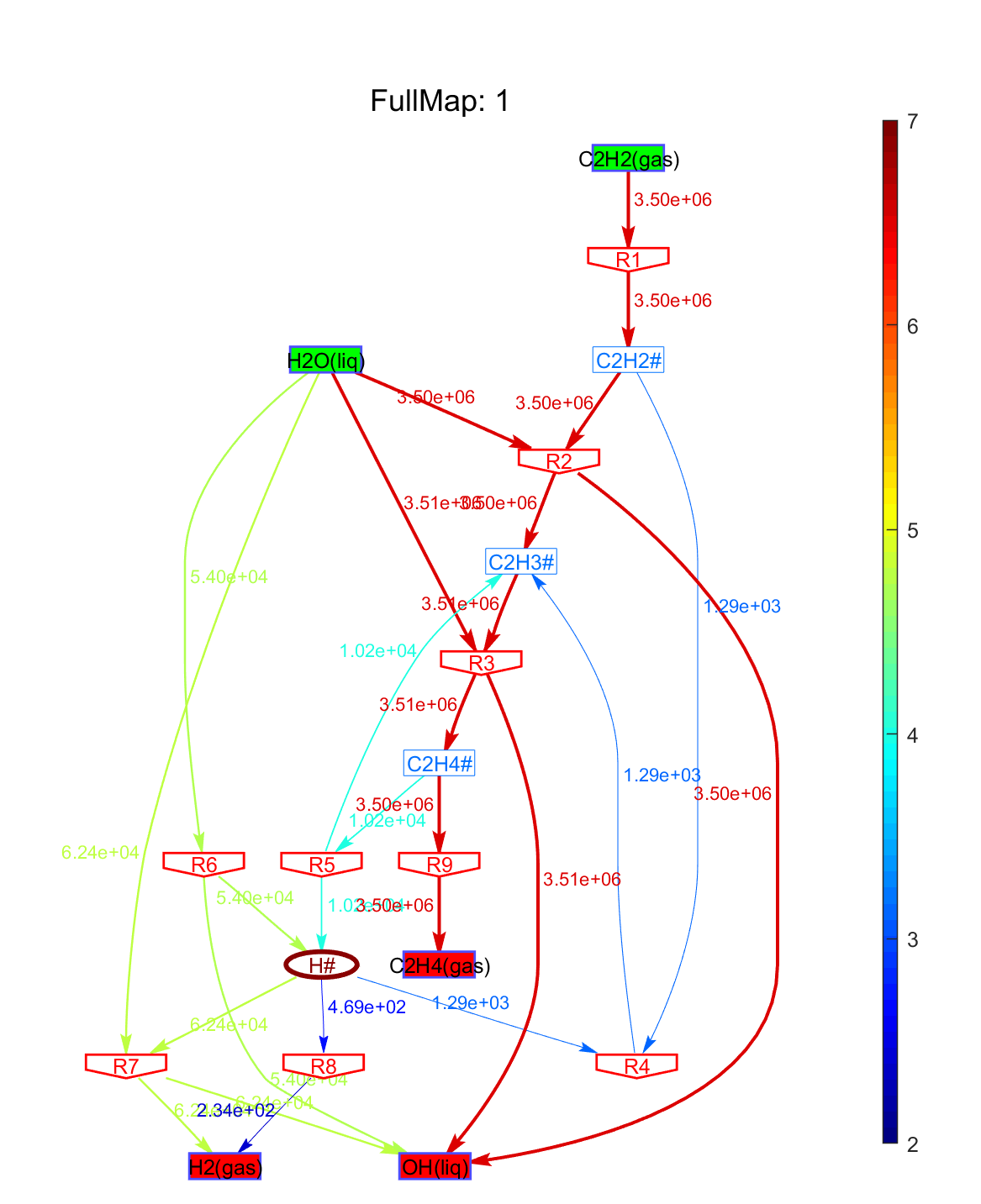


**Figure S82.** The full flow chart of the hydrogenation of acetylene on Cu_3_Au.


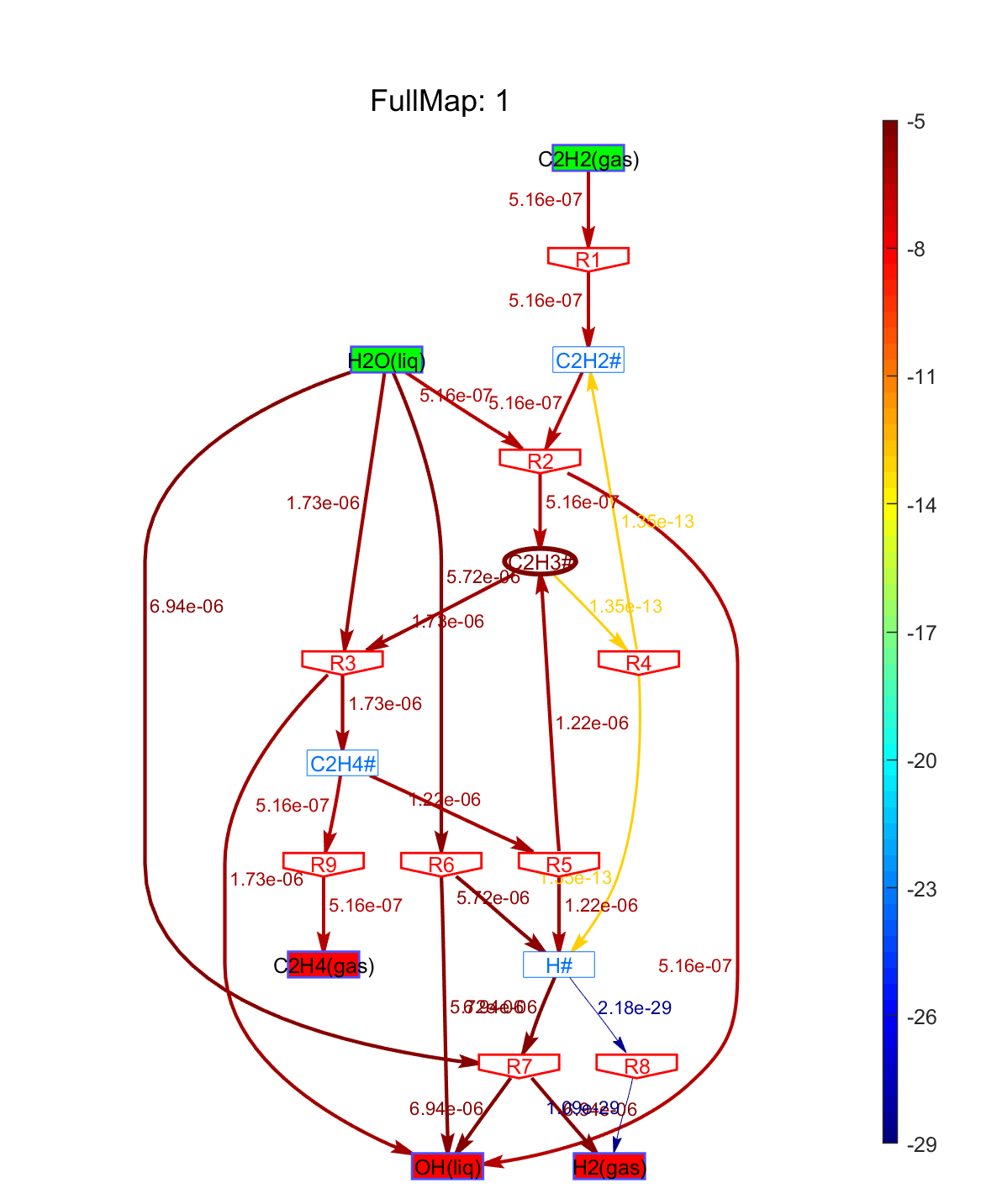


**Figure S83.** The full flow chart of the hydrogenation of acetylene on CuNi_3._

_
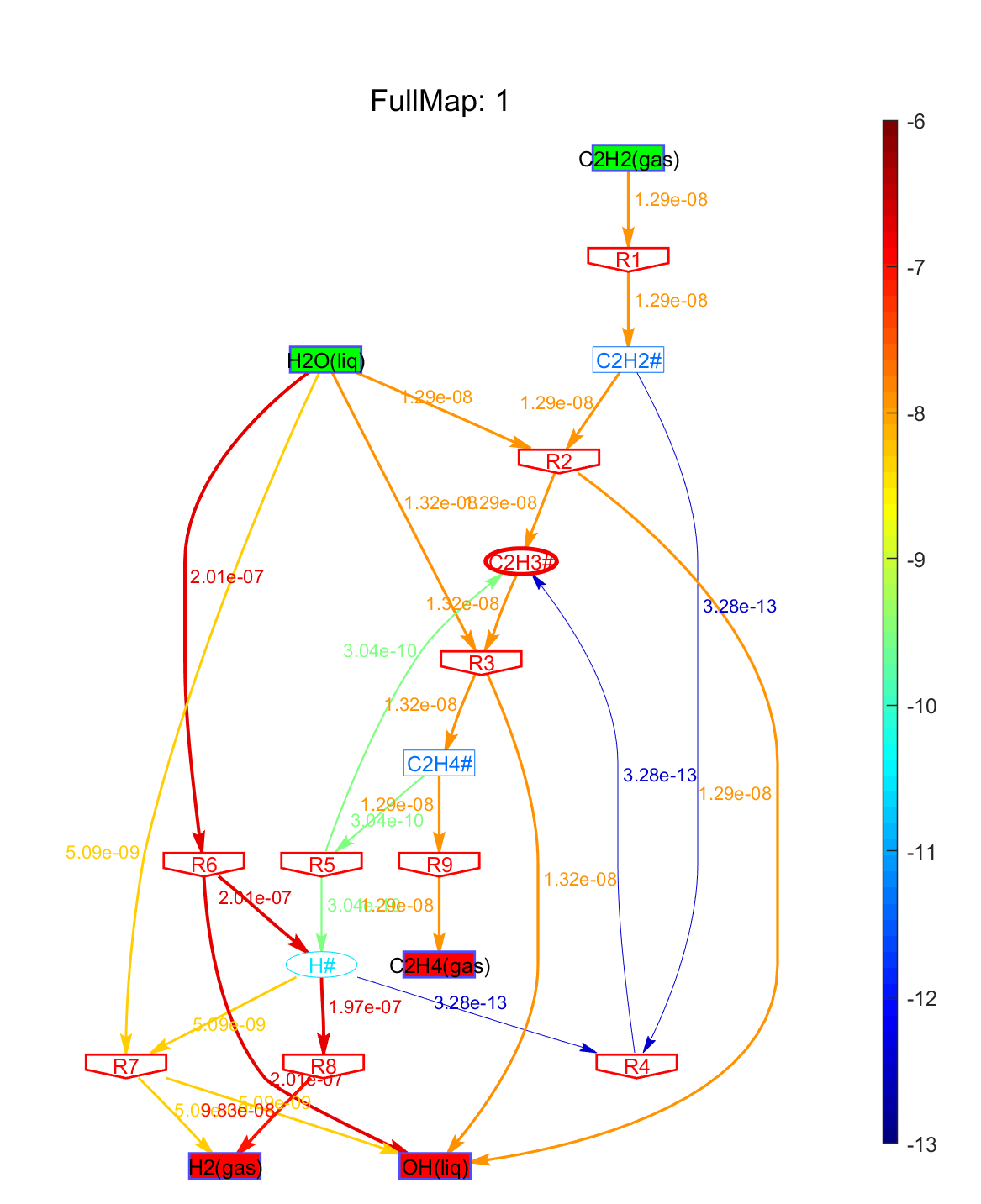
_**Figure S84.** The full flow chart of the hydrogenation of acetylene on CuFe_3._


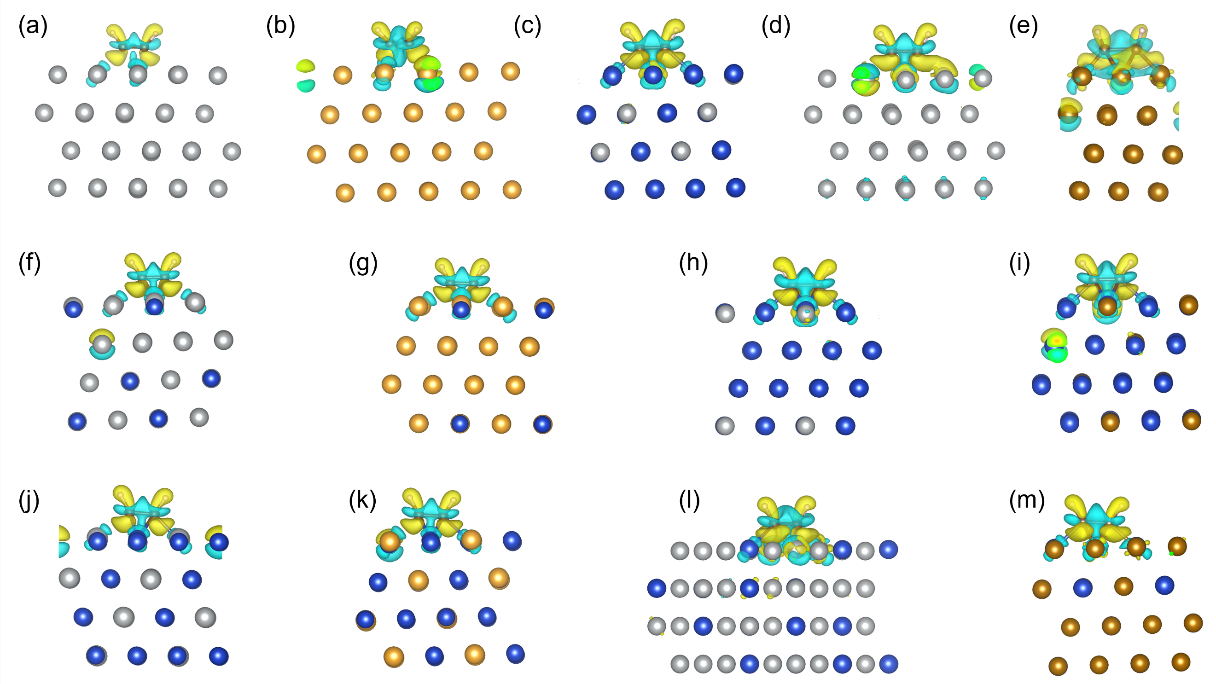


**Figure S85.** The charge density difference when acetylene adsorbed on different metals (a) Ag, (b) Au, (c) Cu, (d) Ni, (e) Fe, (f) CuAg_3_, (g) CuAu_3_, (h) Cu_3_Ni, (i) Cu_3_Fe, (j) Cu_3_Ag, (k) Cu_3_Au, (l) CuNi_3_, (m) CuFe_3_.


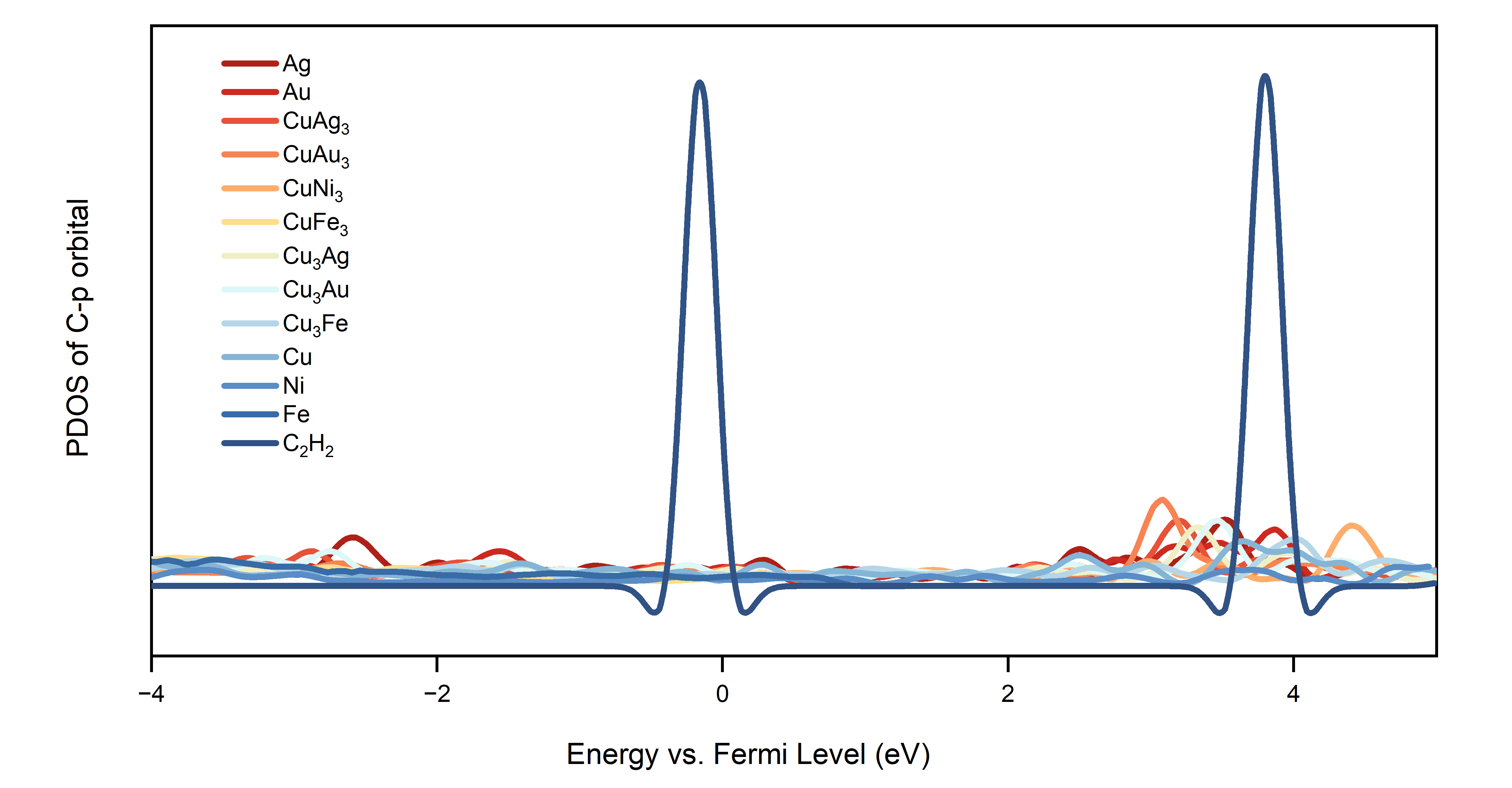


**Figure S86**. Projected density of states (PDOS) of C-2p orbitals of acetylene and when it is adsorbed on different slabs.


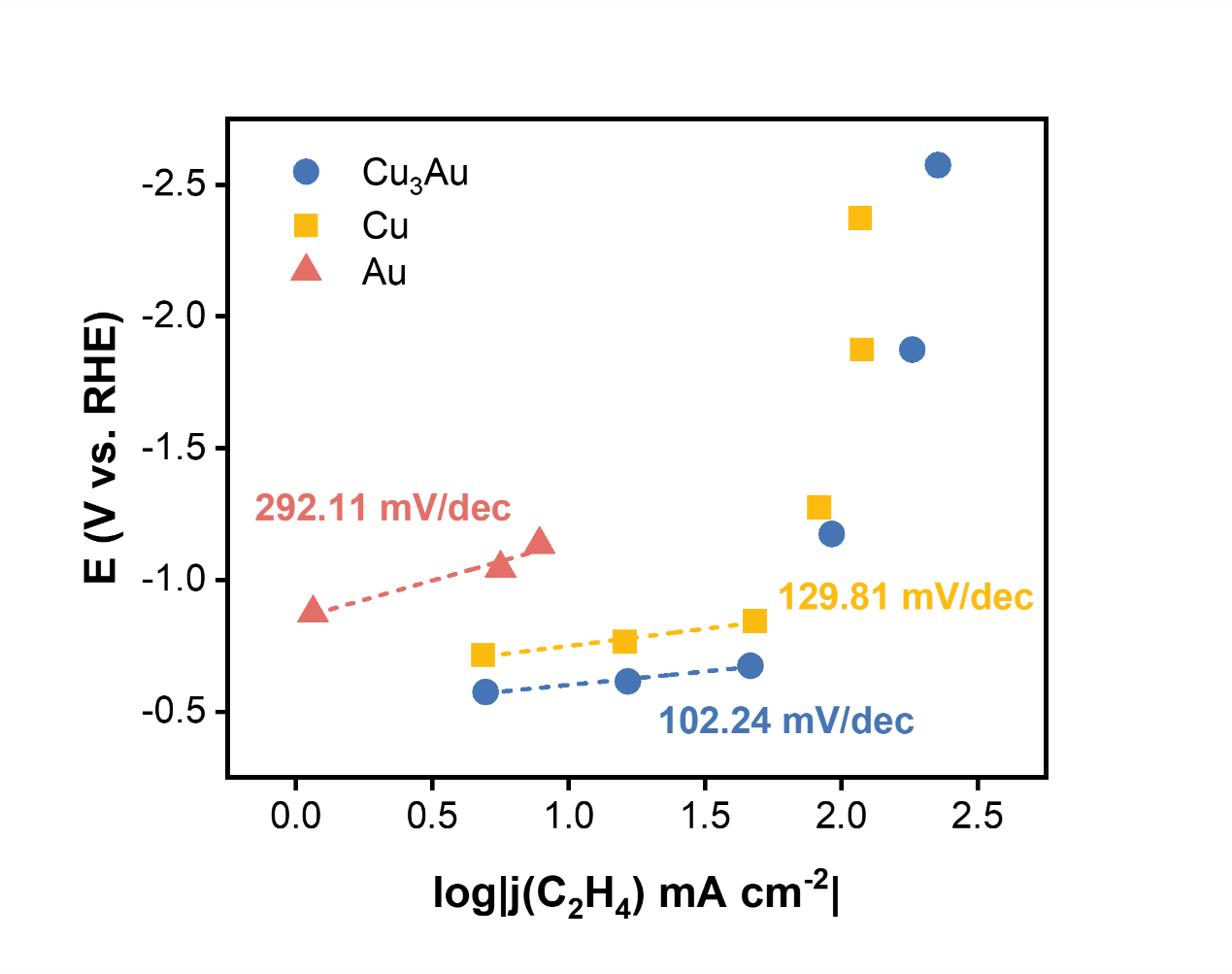


**Figure S87**. Tafel slopes of Cu, Cu_3_Au, and Au. The Tafel slope values are closer to 120 mV/dec, suggesting that the Eley–Rideal (ER) mechanism dominates on the Cu–Au alloy system. This result demonstrates that enhancing the ER pathway can effectively improve the overall reaction rate.


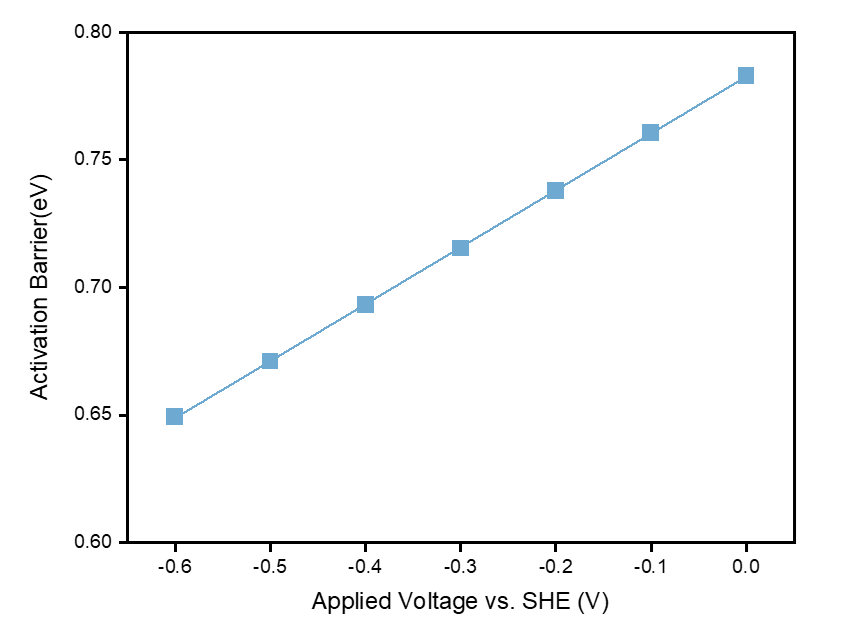


**Figure S88.** The activation barrier obtained by the JDFTx under different potential vs. SHE of the hydrogenation on the *HCCH on Cu.


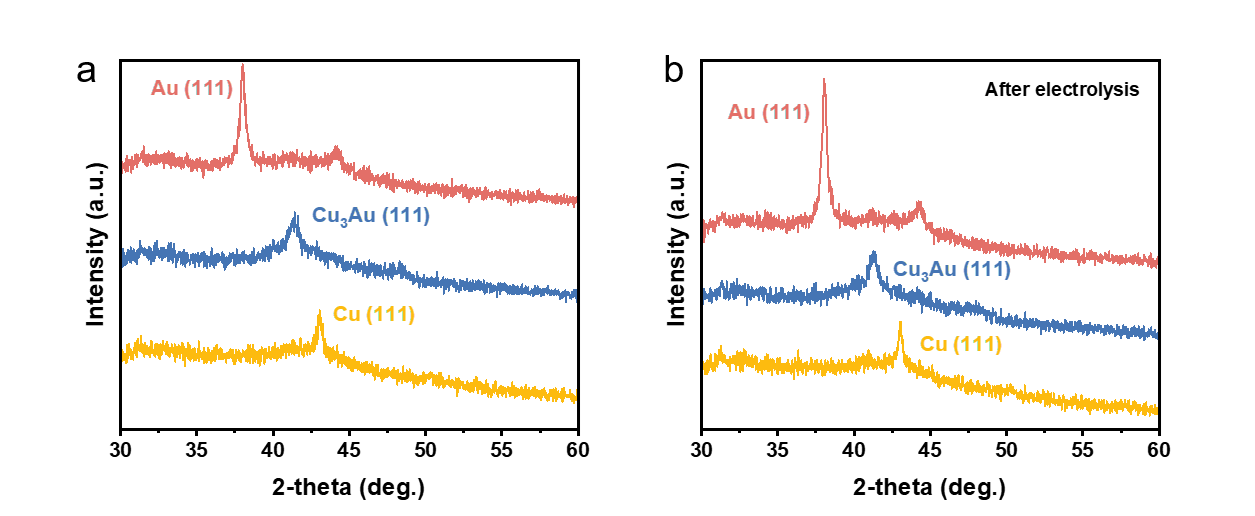


**Figure S89.** The XRD patterns for Cu, Au and Cu_3_Au (a) before and (b) after electrolysis. The structure of Cu_3_Au remains unchanged throughout the reaction, indicating its inherently high catalytic activity. This observation strongly supports both the proposed reaction mechanism and the volcano-type relationship.


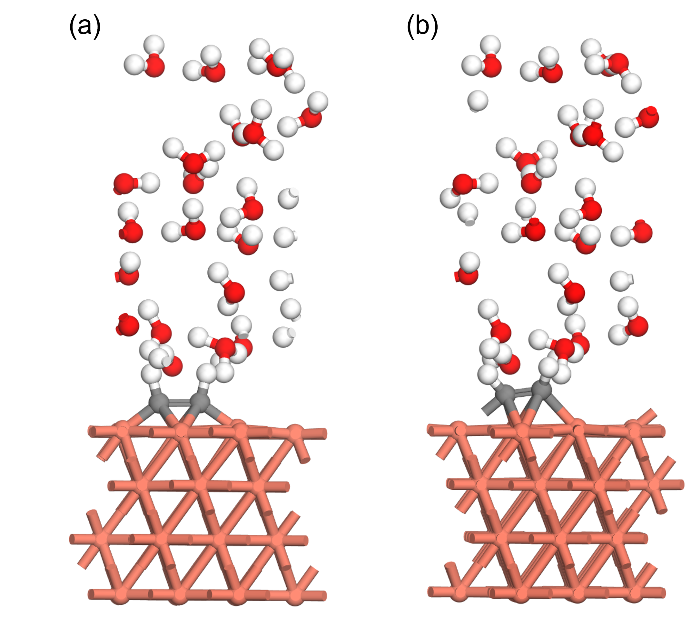


**Figure S90.** Comparison of hydrogenation barriers obtained by the full-solvent model on Cu. The activation barrier calculated with the full-solvent model (0.78 eV) is in close agreement with that from the single-layer water model (0.65 eV), with a difference of only 0.13 eV. Additionally, the reaction coordinate reveals a clear upward displacement of the targeted carbon atom along the Z-axis during the hydrogenation step, consistent with our mechanistic picture. These results confirm that the single-layer water model captures the essential interfacial solvation effects with good accuracy while maintaining computational efficiency, validating its use in the present study.

**Reaction Pathways:**

| R1 | C_2_H_2_(g)+* ↔ *HCCH |
| --- | --- |
| R2 | *HCCH+H_2_O(l) ↔ *HCCH_2_+OH(l) |
| R3 | *HCCH_2_+H_2_O(l) ↔ *H_2_CCH_2_+OH(l) |
| R4 | *HCCH+*H ↔ *HCCH_2_+* |
| R5 | *HCCH_2_+*H ↔ *H_2_CCH_2_+* |
| R6 | H_2_O(l)+ * ↔ *H+OH(l) |
| R7 | *H+H_2_O(l) ↔ H_2_(g)+OH(l)+ * |
| R8 | *H+*H ↔ H_2_(g)+2* |
| R9 | *H_2_CCH_2_ ↔ C_2_H_4_(g)+* |

**Table. S1** The reaction pathway of the whole system. * denotes the adsorbate species, (g) denotes the gas phase, and (l) denotes the liquid states.

|  | 79CuAu | 72CuAg | 82CuNi | 11CuAu | 40CuAg | 6CuAg | Cu |
| --- | --- | --- | --- | --- | --- | --- | --- |
| Cu | 120 W | 120 W | 120 W | 30 W | 50 W | 30 W | 150 W |
| Ni |  |  | 50 W |  |  |  |  |
| Ag |  | 50 W |  |  | 150 W | 150 W |  |
| Au | 50 W |  |  | 200 W |  |  |  |

**Table. S2** The sputtering power for different electrodes.

# 4. Supporting References

[1] G. Kresse, J. Furthmuller, Efficient iterative schemes for ab initio total-energy calculations using a plane-wave basis set *Phys. Rev. B: Condens. Matter* **1996**, *54*, 11169-11186.

[2] G. Kresse, J. Hafner, Ab initio molecular dynamics for liquid metals *Phys. Rev. B: Condens. Matter* **1993**, *47*, 558-561.

[3] J. P. Perdew, K. Burke, M. Ernzerhof, Generalized Gradient Approximation Made Simple *Physical Review Letters* **1996**, *77*, 3865-3868.

[4] S. Grimme, J. Antony, S. Ehrlich, H. Krieg, A consistent and accurate ab initio parametrization of density functional dispersion correction (DFT-D) for the 94 elements H-Pu *Journal of Chemical Physics* **2010**, *132*, 154104.

[5] H. J. Monkhorst, J. D. Pack, Special points for Brillouin-zone integrations *Phys. Rev. B: Condens. Matter* **1976**, *13*, 5188-5192.

[6] G. Kastlunger, P. Lindgren, A. A. Peterson, Controlled-Potential Simulation of Elementary Electrochemical Reactions: Proton Discharge on Metal Surfaces *J. Phys. Chem. C.* **2018**, *122*, 12771-12781.

[7] M. M. Melander, M. J. Kuisma, T. E. K. Christensen, K. Honkala, Grand-canonical approach to density functional theory of electrocatalytic systems: Thermodynamics of solid-liquid interfaces at constant ion and electrode potentials *J. Chem. Phys.* **2019**, *150*, 041706.

[8] K. R. Chan, J. K. Norskov, Electrochemical Barriers Made Simple *J. Phys. Chem. Lett.* **2015**, *6*, 2663-2668.

[9] J. K. Norskov, J. Rossmeisl, A. Logadottir, L. Lindqvist, J. R. Kitchin, T. Bligaard, H. Jonsson, Origin of the Overpotential for Oxygen Reduction at a Fuel-Cell Cathode *J. Phys. Chem. B* **2004**, *108*, 17886-17892.

[10] A. Alavi, P. Hu, T. Deutsch, P. L. Silvestrelli, J. Hutter, CO Oxidation on Pt(111): An Ab Initio Density Functional Theory Study *Phys. Rev. Lett.* **1998**, *80*, 3650-3653.

[11] Z. P. Liu, P. Hu, General rules for predicting where a catalytic reaction should occur on metal surfaces: a density functional theory study of C-H and C-O bond breaking/making on flat, stepped, and kinked metal surfaces *J. Am. Chem. Soc.* **2003**, *125*, 1958-1967.

[12] A. Michaelides, Z. P. Liu, C. J. Zhang, A. Alavi, D. A. King, P. Hu, Identification of general linear relationships between activation energies and enthalpy changes for dissociation reactions at surfaces *J. Am. Chem. Soc.* **2003**, *125*, 3704-3705.

[13] K. Mathew, R. Sundararaman, K. Letchworth-Weaver, T. A. Arias, R. G. Hennig, Implicit solvation model for density-functional study of nanocrystal surfaces and reaction pathways *J. Chem. Phys.* **2014**, *140*, 084106.

[14] K. Mathew, V. S. C. Kolluru, S. Mula, S. N. Steinmann, R. G. Hennig, Implicit self-consistent electrolyte model in plane-wave density-functional theory *J. Chem. Phys.* **2019**, *151*, 234101.

[15] H. Ogasawara, B. Brena, D. Nordlund, M. Nyberg, A. Pelmenschikov, L. G. Pettersson, A. Nilsson, Structure and bonding of water on Pt(111) *Phys. Rev. Lett.* **2002**, *89*, 276102.

[16] S. Goedecker, Minima hopping: an efficient search method for the global minimum of the potential energy surface of complex molecular systems *J. Chem. Phys.* **2004**, *120*, 9911-9917.

[17] J. Rossmeisl, E. Skúlason, M. E. Björketun, V. Tripkovic, J. K. Nørskov, Modeling the electrified solid–liquid interface *Chem. Phys. Lett.* **2008**, *466*, 68-71.

[18] Y. Wang, Z. Wang, C.-T. Dinh, J. Li, A. Ozden, M. Golam Kibria, A. Seifitokaldani, C.-S. Tan, C. M. Gabardo, M. Luo, H. Zhou, F. Li, Y. Lum, C. McCallum, Y. Xu, M. Liu, A. Proppe, A. Johnston, P. Todorovic, T.-T. Zhuang, D. Sinton, S. O. Kelley, E. H. Sargent, Catalyst synthesis under CO_2_ electroreduction favours faceting and promotes renewable fuels electrosynthesis *Nat. Catal.* **2019**, *3*, 98-106.

[19] R. Sundararaman, K. Letchworth-Weaver, K. A. Schwarz, D. Gunceler, Y. Ozhabes, T. A. Arias, JDFTx: software for joint density-functional theory *SoftwareX* **2017**, *6*, 278-284.

[20] R. Sundararaman, W. A. Goddard, 3rd, The charge-asymmetric nonlocally determined local-electric (CANDLE) solvation model *J. Chem. Phys.* **2015**, *142*, 064107.

[21] J. K. Norskov, J. Rossmeisl, A. Logadottir, L. Lindqvist, J. R. Kitchin, T. Bligaard, H. Jonsson, Origin of the Overpotential for Oxygen Reduction at a Fuel-Cell Cathode *J. Phys. Chem. B* **2004**, *108*, 17886-17892.

[22] S. Ringe, C. G. Morales-Guio, L. D. Chen, M. Fields, T. F. Jaramillo, C. Hahn, K. Chan, Double layer charging driven carbon dioxide adsorption limits the rate of electrochemical carbon dioxide reduction on Gold *Nat. Commun.* **2020**, *11*, 33.

[23] W. L. Holstein, M. Boudart, Application of the De Donder Relation to the Mechanism of Catalytic Reactions *J. Phys. Chem. B* **1997**, *101*, 9991-9994.

[24] C. T. Campbell, The Degree of Rate Control: A Powerful Tool for Catalysis Research *ACS Catal.* **2017**, *7*, 2770-2779.

[25] J. Chen, M. Jia, P. Hu, H. Wang, CATKINAS: A large-scale catalytic microkinetic analysis software for mechanism auto-analysis and catalyst screening *J. Comput. Chem.* **2021**, *42*, 379-391.

[26] J. Chen, M. Jia, Z. Lai, P. Hu, H. Wang, SSIA: A sensitivity-supervised interlock algorithm for high-performance microkinetic solving *J. Chem. Phys.* **2021**, *154*, 024108.

[27] J.-F. Chen, Y. Mao, H.-F. Wang, P. Hu, Reversibility Iteration Method for Understanding Reaction Networks and for Solving Microkinetics in Heterogeneous Catalysis *ACS Catal.* **2016**, *6*, 7078-7087.

[28] W. Luc, J. Rosen, F. Jiao, An Ir-based anode for a practical CO_2_ electrolyzer *Catal. Today* **2017**, *288*, 79-84.
